# Dative Bonding in Quasimetallatranes Containing Group 15 Donors (Y = N, P, and As) and Group 14 Acceptors (M = Si, Ge, Sn, and Pb)

**DOI:** 10.1021/acs.inorgchem.4c02532

**Published:** 2024-09-20

**Authors:** Aamy A. Bakry, Matthew G. Fanelli, Martel Zeldin, Kelling J. Donald, Carol A. Parish

**Affiliations:** Department of Chemistry, Gottwald Center for the Sciences, University of Richmond, Richmond, Virginia 23173, United States

## Abstract

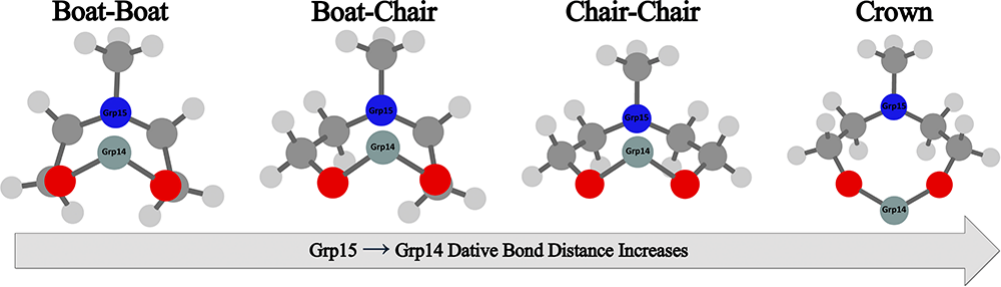

Metallatranes and their analogous fused ring [3.3.0] bicyclic compounds, quasimetallatranes, have emerged as fascinating molecular systems with intriguing structural, bonding, and conformational properties. We present a comprehensive investigation aimed at unraveling the nature of dative bonding and exploring the conformational flexibility of these compounds. We extensively characterize the dative bond between the metal center and the electron pair donor, using a range of modeling techniques. Our analyses involve structural optimizations, molecular orbital examinations, and covalency ratio calculations, which provide a thorough understanding of the bonding interactions responsible for the stability of these systems. The results confirmed the presence of dative bonds, supported by the close proximity between the metal and the electron-donating group, and the observation of overlapping electron density. Our studies reveal a correlation between the size of the electron-donor and the coordinating metal atom, and the strength of the dative interaction, as indicated by the bond length and the Wiberg bond indices. This bond strength, in turn, influences the conformational preferences adopted by these compounds. This investigation sheds light on the fundamental aspects of the fused ring [3.3.0] bicyclic quasimetallatrane compounds and offers valuable insights into their unique properties.

## Abstract 2

Bicyclic metallatrane, or quasi-metallatranes, exists as four different conformations, three of which display dative bonding (boat−boat, boat-chair, and chair−chair). Our analyses involve structural optimizations, molecular orbital examinations, and covalency ratio calculations, which provide a thorough understanding of the bonding interactions responsible for the stability of these molecules.

## Introduction

**Figure 1:**
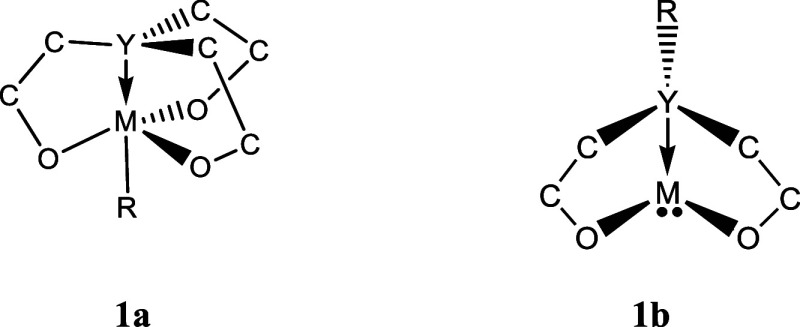
Metallatrane (**1a**) and quasimetallatrane (**1b**) structures. In this work, we studied **1b** with M = Group 14 metalloid or metals with Y = Group 15 donors.

Metallatranes are a class of fused ring [3.3.3.0] tricyclic (Figure 1a) compounds that possess an intramolecular transannular dative bond. Quasimetallatranes are [3.3.0] bicyclic (Figure 1b) compounds that are believed to also contain a dative bond. In both classes of molecules, the dative bond occurs between a main group or transition element (M), which is able to accept electrons (Lewis acid), and a nonmetal (Y), which has at least one lone-pair of electrons (Lewis base) available for coordination. In many instances, Y is an element from group 15 or 16. Since the discovery of the tricyclic metallatranes,^1−5^ there has been considerable interest in these compounds owing to their unusual structural, as well as their physical, biological, and catalytic properties.^6−10^ Researchers have successfully synthesized approximately 300 metallatranes from various ethanolamines, biologically active acids, and essential metals, some of which have displayed promising properties, including antioxidant, immunotropic, and anticancer effects.^11−13^ Spectroscopic (NMR,^14,15^ IR,^16^ PES,^16−20^ diffraction (X-ray,^21−30^ electron,^31−33^) and other physicochemical properties (e.g., dipole moments^6^) strongly support the presence of a dative bond in metallatranes in which the M ← Y bond distance is considerably shorter than the sum of the van der Waals radii (e.g., 3.65 Å for Si ← N)^34^ and somewhat longer than the sum of the covalent radii (e.g., 1.82 Å for Si ← N).^35,36^ There have been some suggestions that shorter distances between Y donors and M acceptors are simply due to crystal packing forces; however, a comparison of boratrane (M = B; Y = N) structural parameters by gas-phase electron diffraction (GPED) and X-ray crystallography, with high-level quantum mechanical calculations on monomeric systems, revealed an agreement that indicates such crystal packing effects are small.^31^

Dative interactions have been studied extensively in tricyclic metallatranes.^8^ Recent reports involving metallatranes and dative bonding include unusual bonding in a disilene complex producing the longest Si=Si bond recorded to date.^37^ In a second recent report, Lo and co-workers showed that solvents with higher polarity stabilize dative bonds. This observation is supported by changes in binding free energies and solvation energies, suggesting the ionic component of the dative bond contributes to solvent-induced stabilization, while solvent polarity affects dative bond strength and interatomic distances, as evidenced by NMR and Raman spectra.^38^ Most recently, the Si ← N dative bond has been subjected to a thorough analysis using the Atoms-In-Molecules (AIM) approach.^39^

Bicyclic fused ring [3.3.0] compounds (Figure 1b) which are analogous to the tricyclic metallatranes, are less well-known but equally fascinating. Following the guidance of Voronkov and Adamovich, we will refer to the bicyclic compounds as quasimetallatrane^11,40,41^; however, it should be noted that “-ocane” is also used in the literature to refer to these bicyclic compounds.^42,43^ These species are structurally similar to metallatranes and capable of a comparable coordinate covalent or dative interaction between the metal and a nearby electron pair donor atom. In such species M(II) should not only serve as a Lewis acid owing to an accessible, vacant valence n*p*, n*d* or hybrid orbital, but also may behave as a Lewis base via an available nonbonding pair of electrons on M. Several members of this class (e.g., M = Ge(II) and Sn(II); Y = N) were first synthesized and characterized by Zeldin and co-workers as early as 1978.^44,45^ At that time, spectroscopic (IR, NMR, MS) evidence supported the presence of the M ← N dative bond and chemical evidence indicated the preservation of the lower oxidation state and the donor capacity of M. In related work, Jurkschat and co-workers reported the crystal structure of a **1b**-like compound where M = Sn(II), Y = N, R = Me with an M ← N bond of 2.447 Å.^46^ The groups of Ignat’ev and Voronkov have reported the molecular structure and vibrational spectra of quasigermatrane^47^ as well as the electronic structure of fluorinated quasisilatrane.^48^

Previous DFT studies on the bicyclic quasimetallatranes have focused on elucidating the structure and vibrational spectra^47^ as well as characterizing the kinetics of the M–O bond cleavage hydrolysis reaction. In the kinetic studies, a symmetrical crown configuration was consistently identified as the favored conformation due to the stabilization provided by transannular interactions between nitrogen and the silicon or germanium atom (M ← N, where M = Si or Ge), alongside intramolecular hydrogen bonding.^49,50^ The energy required for hydrolysis of these compounds depends on factors such as the nature of the M atom and substituent composition, with germanium-containing compounds generally displaying lower enthalpies and Gibbs energies of activation compared to their silicon counterparts.^50^ Additionally, metallatranes exhibit higher activation barriers for hydrolysis compared to acyclic analogs, indicating that the presence of transannular bonds significantly contributes to their structural stability, thus impeding cleavage.^49^

The influence of substituents on the dative bond in both tricyclic and bicyclic metallatranes has been a central focus of research. Substituents bonded to the central element M play a pivotal role in determining the strength and length of the intramolecular M ← Y bond.^42,47,48,51−57^ For instance, in cases where R = F, the M ← N bond strengthens as the number of cycles/rings increases, while in R = H derivatives, it weakens.^48^ The impact of the substituent was more pronounced in silatranes compared to germatranes, although this distinction diminished in tin and lead derivatives. This suggests a potential *nd* orbital participation in M ← N bond formation for heavy Group 14 elements like Sn and Pb, thereby reducing the influence of the substituent.^58^ This sensitivity of the M ← N bond to substituent variations sheds light on the intricate coordination interactions within these compounds.

Given the unusual donor–acceptor nature of M in its low oxidation state and the potential conformational diversity of the rings, we wish to report a comprehensive theoretical investigation of a series of quasimetallatrane compounds of M(II) (e.g., Si(II), Ge(II), Sn(II) and Pb(II)) in fused ring [3.3.0]bicyclic compounds with Y = N, P, and As.

## Computational Methods

All structures were obtained by geometry optimization, without symmetry constraint, using the B3LYP density functional as implemented in the Gaussian 16 program.^59^ For quasimetallatranes containing silicon and germanium atoms, the 6-31G* basis set was used. For quasimetallatranes containing tin and lead, we used the MDF-quality energy-consistent pseudopotentials (ECP) and their corresponding basis sets^60,61^ obtained from the Stuttgart/Cologne group’s Web site.^62^ Specifically, ECP28MDF was applied to tin (Sn), while ECP60MDF was used for lead (Pb).^63^ In the ECP nomenclature, the number following “ECP” indicates the number of core electrons replaced with a pseudopotential; ‘M’ indicates a multielectron fitting process; and ‘DF’ indicates that a fully relativistic Dirac–Fock approach was used in the generation of the ECP. Harmonic frequency analysis was used to confirm all structures as stationary points on their respective potential energy surfaces. To calculate the dative bond vibrational frequencies, we used the same level of theory as the geometry optimization for each congener. Subsequently, the far-infrared (IR) harmonic vibrational frequencies of each molecule were examined for M ← Y vibrational motion. We examined only the far IR region as this is where M ← Y stretches typically occur.^4,44,45,64^ We visualized the motion corresponding to all frequencies. Only frequencies below 1100 cm^–1^ correlated with displacements involving either the metal or the electron donor. To quantify our identification of “dative-like” motion, we focused on the Cartesian coordinates of each normal mode that contained displacements larger than 0.05 Å along the M ← Y axis (corresponding to the *x*-axis). We were also guided by the previous work of Ignatyev and Sundius who reported dative frequencies occurring in the 180–270, 450–500 and above 600 cm^–1^ regions.^65^ Natural Bond Orbital (NBO)^66−69^ and Intrinsic Bond Orbital (IBO)^70^ analyses were performed on each fused ring species using the optimized geometries. IBO calculations were performed with the NWChem software.^71^ All Gaussian generated outputs were visualized using Gaussview6,^72^ while NWChem outputs were visualized with the open source interface Avogadro.^73^

## Results and Discussion

### Conformational Analysis

**Figure 2:**
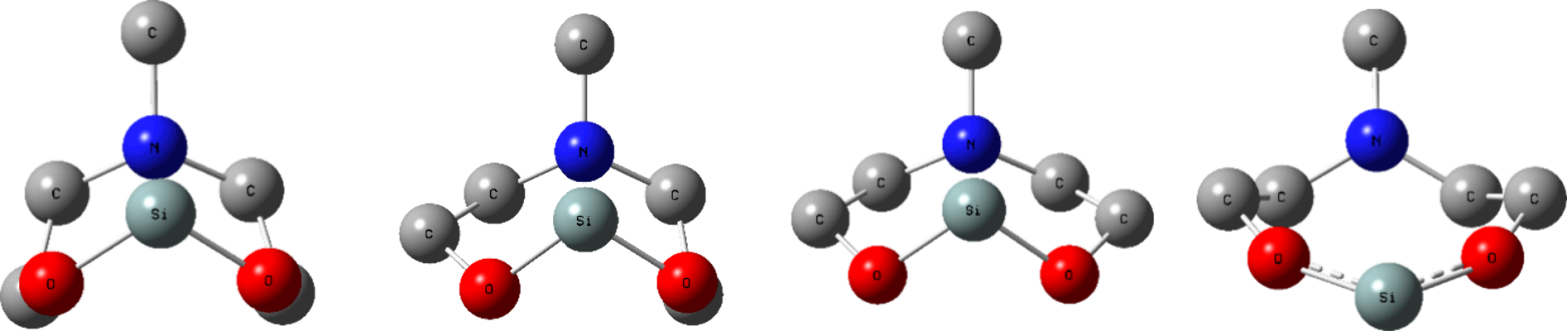
Minimum energy conformations of **1b** obtained using B3LYP/6-31G*. (Si ← N shown) From L ← R: boat–boat, boat-chair, chair–chair, crown. Atom color scheme: red = oxygen, blue = nitrogen, gray = carbon, blue-gray = silicon. Hydrogen atoms are not shown, for clarity. The structures for all other congeners can be found in Table S1. An example of the similarity between congeners of a similar conformation can be seen in the boat–boat optimized geometries shown in Figure S1.

Our data reveal interesting trends in the energetics and conformational preferences of the [3.3.0] bicyclic quasimetallatranes; i.e. the nature of the coordinating metal as well as the donor atom play an important role in determining the favored conformation. Initially, we focused on exploring various ring conformations of **1b** where the R substituent on the electron donor (Y) is a methyl (CH_3_) group. This analysis yielded four different local minima on the potential energy surfaces: boat–boat, boat-chair, chair–chair, and crown conformations (see Figure 2 for M = Si and Y = N; see Table S1 for all other congeners). All of the different congener structures that we studied yield local minima comparable to the structures in Figure 2, with the only difference being the length and angles of each unique congener (Figure S1). In all cases, either the boat–boat or the boat-chair conformations are the global minima (Table 1). Crown and chair–chair conformations lie 2.48 to 17.02 kcal/mol higher in energy. For the heaviest metals (Sn and Pb) the crown conformation could not be found (except for Sn, with P and As) and the energy gap between the lowest-lying boat–boat/boat-chair and chair–chair (and crown) conformations reduces significantly. The nature of the Y donor atom plays a role in conformational behavior. Except in the cases with germanium (Ge), N donors tend to favor boat–boat conformations. Phosphorus (P) donors exhibit more versatile behavior by adopting either boat-chair or boat–boat conformations depending on the coordinating metal atom. Specifically, they favor the boat-chair conformation with smaller metals such as silicon (Si) and germanium (Ge), while preferring the boat–boat conformation with heavier metals such as tin (Sn) and lead (Pb). On the other hand, arsenic (As) donors tend to preferentially stabilize the boat-chair conformation (except for Pb ← P). The relative energies of the boat–boat and boat-chair conformations across the various systems are generally small; differences range from 0.09 to 1.77 kcal/mol. This suggests that both conformations are relatively stable and energetically comparable.

**Table 1:** Comparison of the M ← Y Interaction across the Four Different Conformations Identified in This Study^a^

metal	geometry	M-Y Dist	Rel E + ZPE	dative bond E
Si ← N	**Boat–Boat**	**2.260**	**0.00**	10.12
	Boat-Chair	2.270	0.45	
	Crown	3.605	10.12	
	Chair–Chair	2.240	6.19	
Si ← P	Boat–Boat	3.096	0.73	2.48
	**Boat-Chair**	**2.982**	**0.00**	
	Crown	4.322	2.48	
	Chair–Chair	3.559	3.14	
Si ← As	Boat–Boat	3.185	0.64	4.75
	**Boat-Chair**	**3.109**	**0.00**	
	Crown	4.439	4.75	
	Chair–Chair	3.305	2.91	
Ge ← N	Boat–Boat	2.248	0.19	17.02
	**Boat-Chair**	**2.268**	**0.00**	
	Crown	3.552	17.02	
	Chair–Chair	2.251	6.18	
Ge ← P	Boat–Boat	2.661	1.36	8.38
	**Boat-Chair**	**2.694**	**0.00**	
	Crown	4.479	8.38	
	Chair–Chair	2.663	4.60	
Ge ← As	Boat–Boat	2.755	1.77	10.23
	**Boat-Chair**	**2.789**	**0.00**	
	Crown	4.579	10.23	
	Chair–Chair	2.763	4.26	
Sn ← N	**Boat–Boat**	**2.483**	**0.00**	
	Boat-Chair	2.480	0.75	
	Crown			
	Chair–Chair	2.457	6.28	
Sn ← P	**Boat–Boat**	**2.804**	**0.00**	10.16
	Boat-Chair	2.836	0.23	
	Crown	4.226	10.16	
	Chair–Chair	2.812	4.07	
Sn ← As	Boat–Boat	2.932	0.37	7.92
	**Boat-Chair**	**2.964**	**0.00**	
	Crown	4.121	7.92	
	Chair–Chair	2.943	3.54	
Pb ← N	**Boat–Boat**	**2.583**	**0.00**	
	Boat-Chair	2.578	0.53	
	Crown			
	Chair–Chair	2.565	5.81	
Pb ← P	**Boat–Boat**	**2.862**	**0.00**	
	Boat-Chair	2.892	0.09	
	Crown			
	Chair–Chair	2.866	3.69	
Pb ← As	Boat–Boat	2.963	0.44	-
	**Boat-Chair**	**2.991**	**0.00**	
	Crown			
	Chair–Chair	2.967	3.33	

a Shown are the M ← Y dative bond lengths (Å), the relative energies (kcal/mol) of each conformation for a given M and Y, and the approximate energy of the dative bond. We approximate this energy as the difference between the global minima and the non-dative crown congener. This approximation to the dative bond energy was chosen because crown conformers typically lack a dative interaction and are thus useful as a reference structure comparison. The compounds containing silicon and germanium were calculated without using ECPs, while tin and lead were calculated using the MDF ECPs. In the table below, the global minimum energy conformer is highlighted in bold.

In almost all cases, the crown is the highest-lying conformation–the only exception is for M = Si and Y = P, where both the crown and chair–chair conformations are isoenergetic and high-lying relative to the boat–boat and boat-chair structures. Crown conformations are the only isomers with M ← Y interaction distances longer than the sum of the van der Waals radii and considerably longer M ← Y distances than in the corresponding lower-lying boat–boat and boat-chair isomers. This energetic ordering of the various conformations reflects the stability imparted by the presence of a dative bond.

For all systems except those containing lead (Pb), as well as Sn ← N, all four conformations were confirmed by frequency analysis as minima on the respective surfaces; for the exceptional cases, the only conformer not located in our structural optimizations was the crown. The dative bond plays an important role in the conformational flexibility of **1b**. For instance, if a strong dative bonding interaction is present, the molecule is best considered as a bicyclic system containing two conformationally constrained five-membered rings. On the other hand, if the dative bond is weak or absent, the system can be thought of as a conformationally flexible eight-membered ring. What is the balance between the driving force for dative bonding versus conformational flexibility? Based on the data in Table 1, we conclude that in almost all cases the quasimetallatranes are flexible enough to adopt at least four conformations; and, in three of the four conformations, a dative bond is present. In almost all cases, the conformations with the dative bond lie lower in energy than those lacking such an interaction; for smaller metals the stability imparted by the presence of a dative bond is more significant than for larger metals.

Our data show that the boat–boat conformation consistently exhibits the shortest M ← Y bond length and the lowest relative energy in most cases. The boat-chair and chair–chair conformations also maintain a dative interaction between the metal and donor, albeit with slightly longer bond lengths and higher relative energies. The crown conformation deviates from this trend, by displaying significantly longer M ← Y separations in some cases, or no dative interaction in many cases, and substantially higher relative energies. Because the interaction distance between M and Y in the crown conformation is so long, we can associate the conformational isomerization energy with the breaking of the dative bond and approximate the dative bond energy as
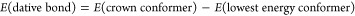


Using this equation, the dative bond energy is estimated to range between 2.48 (Si ← P)–17.02 (Ge ← N) kcal/mol (Table 1).

### Structural Analysis

In all cases of the boat–boat and boat-chair conformer, the distance between the metal and group 15 elements is considerably shorter than the sum of the van der Waals radii and somewhat longer than the sum of the covalent radii, which provides evidence for a bonding interaction between the metal and the electron donating group and indicates the presence of a dative bond (Table 2). We examined dative bond lengths as a function of different metal atoms (Si, Ge, Sn, Pb), donor groups (N, P, As), and diverse substituents (H, Me, tBut, OH, CN). The remainder of this report will focus on the boat–boat conformation as we observed that all congeners contain low-lying boat–boat minima on their corresponding surfaces.

**Table 2:** Dative Bond Distances (Å) of the Boat–Boat Congener with a Methyl R Group, Compared to the Sum of the van der Waals and Covalent Radii (Å) as Reported in the Literature (Ref (34) (vdW radii) and Refs (35,36) (Covalent Radii)^a^

	calculated	literature values	
bond	6-31G*/MDF	Σ *R*_vdw_(34)	Σ *R*_cov_(35,36)
Si ← N	2.26	3.65	1.82
Si ← P	3.10	3.90	2.18
Si ← As	3.18	3.95	2.30
Ge ← N	2.25	3.55	1.91
Ge ← P	2.66	3.80	2.27
Ge ← As	2.75	3.85	2.39
Sn ← N	2.48	3.72	2.10
Sn ← P	2.80	3.97	2.46
Sn ← As	2.93	4.02	2.58
Pb ← N	2.58	3.57	2.17
Pb ← P	2.86	3.82	2.53
Pb ← As	2.96	3.87	2.65

a The 6-31G* level of theory was used for the germanium- and silicon-containing compounds and the MDF ECP was used for the tin- and lead-containing compounds.

### Metal Dependence

In nitrogen-containing compounds, dative bond distances generally increase as we increase the size of the M atom from silicon (Si) to lead (Pb). Larger metal atoms have larger and more diffuse electron clouds, leading to longer bond distances. This trend is not consistent across all donor groups and geometries, which suggests that different congeners exhibit unique bonding behaviors. For instance, dative bond lengths with phosphorus (P) and arsenic (As) are less sensitive to the increasing size of the M atom from silicon (Si) to lead (Pb). This may be due to the increased polarizability and decreased electronegativity of P and As, relative to N. This effect is coupled with the increased polarization of M by the neighboring O atoms as M gets bigger and softer.

### Donor Group Influence

The donor group also plays a crucial role in determining dative bond distances. Bonds with nitrogen (N) donors tend to be shorter compared to those with phosphorus (P) or arsenic (As) donors. The differences in bond lengths can be attributed to the differences in the size and electronegativity of the electron donors. Nitrogen, being smaller, forms shorter and potentially stronger bonds compared to phosphorus and arsenic. As we move down the group, the size of the electron donor increases and leads to generally longer bonds. However, this picture can be complicated by the polarizability of the metal partner; with Si and Ge having smaller polarizabilities (37.3 and 40 a.u., respectively^74^) than Sn and Pb (53 and 47 a.u., respectively^74^). In some cases, the increasing polarizability of the metal atom can lead to stronger interactions with larger electron donors, which results in shorter dative bonds. This phenomenon has been noted in simple F_4_M ← NR_3_ complexes where even the Pb ← N distance may be shorter than the Si ← N distance, depending on the identity of R.^75^

### Effect of Substituents

Substituents (H, Me, tBu, OH, CN) have a significant impact on dative bond distances. Electron-withdrawing substituents, such as cyanide (CN) and hydroxyl (OH), generally lead to longer dative bond distances compared to hydrogen (H) or methyl (Me) substituents. This suggests that the presence of electron-withdrawing groups weakens the metal-donor dative bonds. For instance, in the silicon–nitrogen (Si ← N) boat–boat geometry, bonds with H or Me substituents are notably shorter than those with OH or CN groups (Table S2).

### Conformational Effects

As discussed in the section above, conformer geometry also affects dative bond distances. Among the conformations identified, the boat–boat geometry was the one most able to position the M close enough to the Y donor atom to consistently yield the shortest dative bond distances (Table 1). Interestingly, chair–chair conformations were able to form relatively short dative bonds (Table 1); however, the stabilizing effect of the dative bond is not enough to outweigh the strain associated with the adoption of a chair conformation which leads to chair–chair structures that are higher in energy by 3–6 kcal/mol.

### Harmonic Frequency Analysis

We calculated the infrared (IR) stretching vibrations of the dative bonds within our quasimetallatranes (Table 3) in order to guide spectroscopists in future studies of these molecules, as well as to provide an additional gauge of the strength of the dative bond. An extensive study by Ignatyev & Sundius using halogen-substituted tricyclic metallatranes. identified three main frequencies for dative vibrations: 180–270 cm^–1^, 450–500 cm^–1^, and above 600 cm^–1^. While a single frequency cannot be correlated with a particular bond vibration, they noted that the lowest frequency vibrations are the most “pure″, primarily involving Si ← N or Ge ← N stretching with minimal contributions from other coordinates.^65^ We visualized the motions corresponding to all frequencies to identify those most associated with dative bonding. This visualization was coupled with an examination of the normal modes of motion associated with each frequency. For each quasimetallatrane, we see frequencies that fall within the Ignatyev & Sundius ranges, as well as frequencies that lie even further into the far-IR with relatively significant intensities. Our analysis suggests that these lowest frequencies are the most representative of the M ← Y bond as they have minimal contributions from other atom movements. Shown in Table 3 are those far IR frequencies, as well as those that fall within the Ignatyev & Sundius ranges. In all cases, these frequencies contain significantly large M or Y displacements, and relatively strong IR intensities. All computed far-IR frequencies can be found in Table S3. The previously estimated experimental vibrational frequency range for Sn ← N of 198–260 cm^–1^ aligns well with the 180–270 cm^–1^ range predicted by Ignatyev & Sundius on the basis of tricyclic metallatranes. Our calculations on the Sn ← N quasimetallatrane reveal a relatively intense peak at 229 cm^–1^, in agreement with these previous reports. Interestingly, the only other experimentally measured frequency for a quasimetallatrane is for the Ge ← N system, and that frequency was reported to occur at 545 cm^–1^. Our results show that while there are two intense peaks near 550–560 cm^–1^ in the IR spectrum of Ge ← N quasimetallatrane, neither frequency corresponds to a dative bonding motion.

**Table 3:** Dative Bond IR Stretching Vibrations (cm^–1^) for All Compounds Studied^a^^,^^b^^,^^c^

		IR region (cm^–1^)		
bond	below 180	180–270	450–500	experiment
Si ← N	156	225	470	
Si ← P	42	180	397	
Si ← As	67	168	389 or 540	
Ge ← N	143	256	427	545^44^
Ge ← P	94	186		
Ge ← As	73	172	555	
Sn ← N	128	229	467	198–260,^45^ 454^76^
Sn ← P	70	246	422	
Sn ← As	48	253	554	
Pb ← N	110	207	434	
Pb ← P	71	265	484	
Pb ← As	51	235	555	

a All data obtained using B3LYP method and the 6-31G* (for Si and Ge) the MDF ECPs (for Sn and Pb) and scaled by 0.9641 for 6-31G* results, and 0.961 for the MDF results. The full set of frequency results below 600 cm^–1^ are shown in Figure S3.

b The 6-31G* basis sets are used for the germanium- and silicon-containing compounds and the MDF ECPs and basis sets are used for the tin- and lead-containing compounds.

c The frequencies calculated using the 6-31G* and MDF basis sets were scaled using 0.9641 and 0.961 scale factors obtained from the Computational Chemistry Comparison and Benchmark DataBase of the National Institute of Standards and Technology: https://cccbdb.nist.gov/vibscalejust.asp.^77^

Based on our vibrational data, we can conclude that dative bonds containing nitrogen atoms are the strongest since they have the highest frequency values across different metals. Generally, the strength of the dative bond decreases as the donor atom increases in size (N → P → As); the compound containing the Si ← P bond is exceptionally weak (42 cm^–1^, after scaling). Dative bonds containing nitrogen atoms also decrease in strength as the metal atom increases in size (Si → Ge → Sn → Pb). However, this trend is not consistent for either phosphorus or arsenic.

### Molecular Orbital Analysis

**Figure 3:**
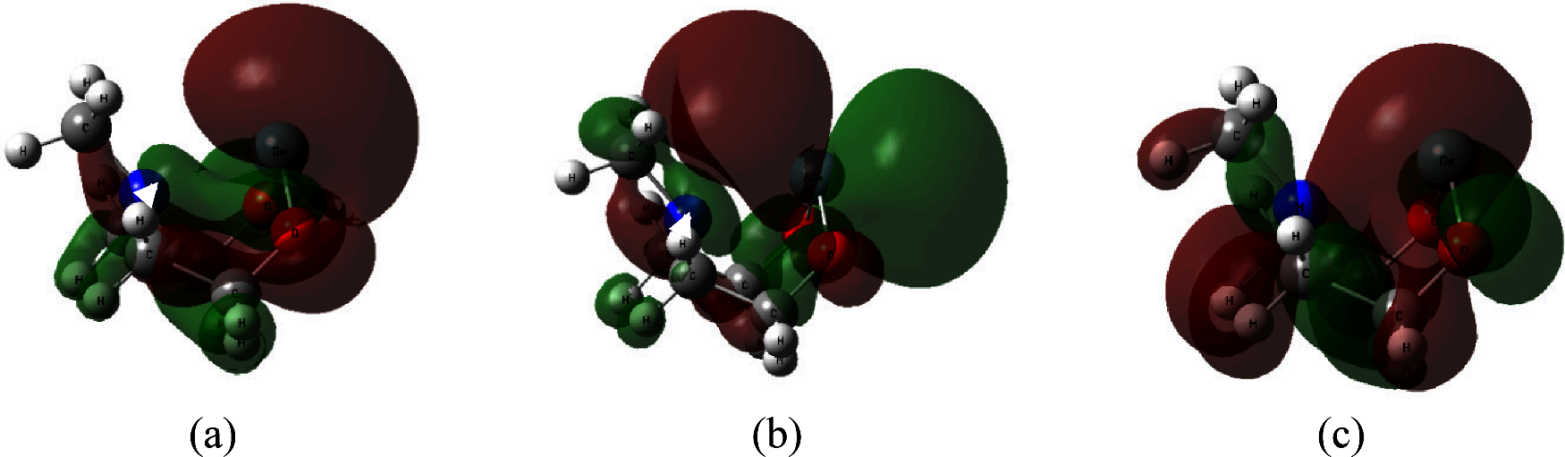
Molecular orbitals (MOs) for the germanium and nitrogen-containing compound in the boat–boat conformation are shown plotted at an isovalue of 0.02 atomic units. These MOs were obtained at the B3LYP/6-31G* level of theory. The three molecular orbitals above show the (a) HOMO, (b) LUMO, and (c) dative bond.

We have explored the electronic properties of the different metal–donor complexes. Specifically, we focused on the molecular orbital (MO) energies and energy differences (ΔEnergy) at the optimized ″boat–boat″ geometry (Table S3). We looked for evidence of dative bonding by examining the highest occupied molecular orbital (HOMO), the lowest unoccupied molecular orbital (LUMO), and the ten orbitals below the HOMO for evidence of electron density between the metal and the electron-donating nitrogen, phosphorus, and arsenic atoms (Table S3). However, in all of the compounds studied, neither the HOMO nor the LUMO contained electron density between Y and M, which suggests that electronic excitation will not easily disrupt the bicyclic structure of these compounds. It should be noted that in some cases there is evidence of a dative bond in the HOMO–1 molecular orbital. Additionally, for some of the compounds, there is evidence of dative bonding in several of the lower energy occupied molecular orbitals. Figure 3 highlights three of the Ge ← N molecular orbitals that are representative of the HOMO (3a), LUMO (3b) and dative (3c) orbitals that we see in these compounds. In Figure 3c, we can see the red wave function lobe linking the N atom (blue) and the germanium atom (gray), suggesting the presence of a dative bond.

The analysis of MO energies revealed systematic trends across different donors and metals. Generally, HOMO and LUMO energies become more negative from N to P to As donors, indicating a decrease in ionization energy, and emphasizing the important role that the donor atom plays in the electronic properties of these molecules. The HOMO and LUMO energy differences (Δ*E* (LUMO (L) – HOMO(H))) provided insight into quaismetallatranes stability and reactivity. For silicon-containing compounds, the HOMO–LUMO gap narrows as the donor changes from N to P to As for each metal. However, for germanium-containing compounds, the HOMO–LUMO gap narrows as the donor changes from As to N to P. For tin- and lead-containing compounds, the trend is completely different where Δ*E* (L-H) values decrease from N to As to P donors. Across all congeners, the lead-containing compounds had the lowest Δ*E* (L-H) values while germanium-containing compounds had the highest values. This suggests that some complexes and their reactivities may be more prone to electronic transitions than others, potentially influencing their chemical behavior. Moreover, the extent of this influence depends largely on both the metal and the electron donor present in the system. Our results are in relatively good agreement with those reported previously by Karlov and colleagues who used the PBE functional with the TZ2P basis set to calculate the HOMO–LUMO energy gaps for methyl substituted Sn ← N (4.59 eV or 0.169 au) and Ge ← N (5.48 eV or 2.01 a.u.)^78^. Our values for the same systems are 0.197 and 0.217 a.u., respectively (Table S3).

### Covalency Ratio–A Measure of Bonding

The covalency ratio (χ) (eq 1) serves as a key metric in assessing the nature of a chemical bond and provides a dimensionless measure of the bond’s covalent character; as such it can be used as a metric for the existence of a dative bond. The closer the covalency ratio is to unity, the greater the chemical overlap, or bonding forces, between two atoms.^66^^,^^79^
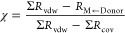
1

One of the prominent findings in our results is the variation in covalency ratios (χ) across different metal-donor combinations and conformations (Table 4). The covalency ratios range from approximately 0.53 to 0.79, reflecting the extent of dative bonding between the donor and the metal. Notably, most compounds in their boat–boat and boat-chair conformations exhibit χ values around 0.7, indicating a substantial degree of bonding.

**Table 4:** Electronic Composition of Dative Bonds in All Conformations of All Compounds (except for Crown Conformations of Sn and Pb as neither IBO or NBO Analyses Showed Dative Interactions in These High Energy Conformations)^a^

		NBO			IBO		χ
metal	geometry	%M	%Y	Wiberg bond index	%M	%Y	
Si ← N	Boat–Boat	7.33	92.67	0.243	13	83	0.76
	Boat-Chair	7.48	92.52	0.247	13	83	0.75
	Chair–Chair	7.70	92.30	0.256	14	83	0.77
	Crown			0.008			
Si ← P	Boat–Boat			0.238	3	96	0.47
	Boat-Chair	3.71	96.29	0.239	4	94	0.53
	Chair–Chair			0.069			0.20
	Crown			0.002			–0.25
Si ← As	Boat–Boat			0.067			0.46
	Boat-Chair			0.128	1	98	0.51
	Chair–Chair			0.036			0.39
	Crown			0.002			
Ge ← N	Boat–Boat	8.09	91.91	0.271	14	82	0.79
	Boat-Chair	8.22	91.78	0.276	14	82	0.78
	Chair–Chair	8.37	91.63	0.2880	15	81	0.79
	Crown			0.013			0.00
Ge ← P	Boat–Boat	13.53	86.47	0.389	13	84	0.74
	Boat-Chair	13.21	86.79	0.350	12	86	0.72
	Chair–Chair	13.54	86.46	0.376	13	85	0.74
	Crown			0.003			–0.44
Ge ← As	Boat–Boat	12.13	87.87	0.234	5	94	0.75
	Boat-Chair	11.98	88.02	0.245	6	93	0.73
	Chair–Chair	12.20	87.80	0.218	5	94	0.74
	Crown			0.003			–0.50
Sn ← N	Boat–Boat	7.03	92.97	0.234	16	80	0.77
	Boat-Chair	7.14	92.86	0.238	16	80	0.76
	Chair–Chair	7.27	92.73	0.244	17	80	0.77
Sn ← P	Boat–Boat	12.05	87.95	0.380	20	87	0.77
	Boat-Chair	11.75	88.25	0.373	20	87	0.75
	Chair–Chair	11.97	88.03	0.387	20	87	0.77
Sn ← As	Boat–Boat	6.33	93.67	0.324	13	85	0.76
	Boat-Chair	7.80	92.20	0.322	13	85	0.74
	Chair–Chair	7.49	92.51	0.333	14	85	0.75
Pb ← N	Boat–Boat	4.61	95.39	0.218	18	78	0.71
	Boat-Chair	5.44	94.56	0.225	19	77	0.71
	Chair–Chair	6.93	93.07	0.234	19	77	0.72
Pb ← P	Boat–Boat	11.75	88.25	0.366	24	74	0.74
	Boat-Chair	11.51	88.49	0.364	23	75	0.72
	Chair–Chair	11.79	88.21	0.383	24	74	0.74
Pb ← As	Boat–Boat	6.68	93.32	0.330	15	83	0.74
	Boat-Chair	8.05	91.95	0.330	15	83	0.72
	Chair–Chair	8.56	91.44	0.350	16	82	0.74

a The silicon- and germanium-containing compounds are calculated at B3LYP/6-31G* level of theory. Tin and lead-containing compounds are calculated with the MDF ECP. Metal and donor hybrid orbital composition (%M and %Y), along with orbital energies, were obtained using NBO and IBO analyses. (NBO orbital decomposition values can be found in Table S4). NBO was also used to determine Wiberg indices for the M -- Y interactions. The covalency ratio (χ) is also reported. This is a dimensionless ratio measuring the degree of electronic overlap in a chemical bond.

**Figure 4:**
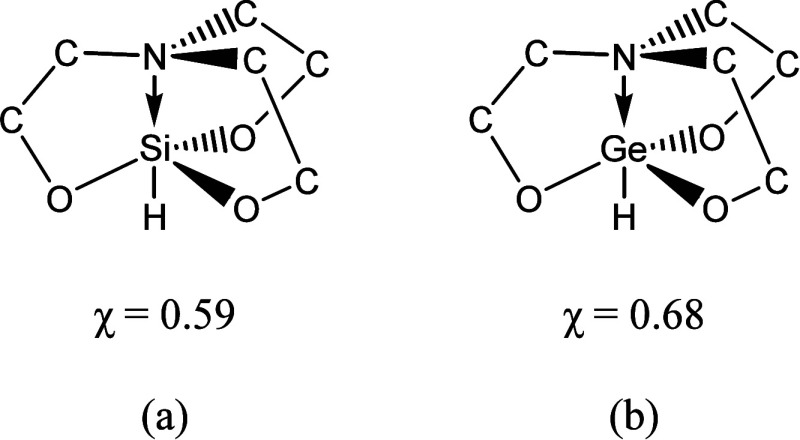
Tricyclic metallatrane compounds reported by Milov and their corresponding covalency ratios. (a). Si ← N and (b). Ge ← N containing metallatranes. The covalency ratio (χ) is a dimensionless ratio measuring the chemical overlap forces.

Compounds containing germanium (Ge) generally exhibit slightly higher covalency ratios than their silicon (Si) counterparts, highlighting the metal-dependent effect on dative bonding. Additionally, evidence for donor-dependency in dative bonding is shown by compounds with phosphorus (P) donors exhibiting lower χ values than those with nitrogen (N) donors. Conformational changes within these [3.3.0] bicyclic compounds also impact dative bonding as evidenced by changes in the covalency ratios. In some cases, variations in conformation lead to slight differences in χ values, emphasizing the sensitivity of covalent character to molecular geometry. Milov reports a covalency ratio of 0.59 in the [3.3.3.0] tricyclic compound that contains silicon and nitrogen (Figure 4a) and a covalency ratio of 0.68 in the tricyclic compound that contains germanium and nitrogen (Figure 4b).^79^ These covalency ratios are smaller than those determined for the [3.3.0] bicyclic compounds in our study, suggesting that dative interactions may be stronger in the tricyclic atranes.

### Natural Bond Orbital (NBO) and Intrinsic Bond Orbital (IBO) Analyses

Localized molecular orbital analysis has been a widely employed tool in the field of computational quantum chemistry for understanding and characterizing chemical bonds, especially those with strong donor–acceptor interactions.^80^ In this study, we utilized and compared two localization schemes; namely, the Natural Bond Orbital (NBO)^66,67,81^ and Intrinsic Bond Orbital (IBO)^70,80^ analyses. We used these methods to probe the electronic composition of dative bonds in [3.3.0] bicyclic compounds containing various metals (Si, Ge, Sn, Pb) and donors (N, P, As). By design, orbital localization methods are predicated on the assumption that wave functions can be transformed as localized orbitals which accurately represent chemical bonds and lone pairs of electrons. As such, these analyses provide valuable insights into the electronic structure, charge transfer, and bond covalency in a molecule.^66,67,82^ We utilized two different localization schemes as they adopt different approaches in their unitary transformation of the canonical orbitals. NBO emphasizes two-center bonds and one-center lone pairs which produces results most similar to Lewis structure theory,^69,83^ while the IBO transformation maximizes atomic populations in the localized molecular orbitals.^84^ Concerns have been raised regarding the sensitivity of NBO results to basis set superposition error (BSSE), which can lead to incorrect conclusions in intermolecular interactions.^85^

In Tables 6 and S4–5, we present Natural Bond Orbital (NBO) and Intrinsic Bond Orbital (IBO) data providing insights into the electronic composition of dative bonds in the [3.3.0] bicyclic quasimetallatranes compounds containing various metals and donors. NBO analysis confirms a dative interaction for all congeners and all noncrown conformations except for arsenic and phosphorus donors with the smaller Si metal. When arsenic and phosphorus are combined with Si, the NBO decomposition analysis (Table 4) did not indicate a dative bond even in the boat–boat conformation. IBO analysis did show some amount of dative bonding for the boat–boat and boat-chair conformers of Si ← P as well as for the boat-chair of Si ← As, but the percent contributions are very small and the metal contributions to the dative interaction are less than 4%. For arsenic with silicon, the covalency ratio was also low (χ range 0.39–0.51); however, the data presented above (dative distances, MOs, etc.) does suggest dative bonding. For phosphorus with silicon, the NBO and IBO analyses did not indicate Si ← P interaction except in the boat-chair conformation, which had a χ value of 0.53. This value is slightly higher than that of other conformations, such as the boat–boat (χ = 0.47), further suggesting a dative interaction in the boat-chair conformation. Furthermore, the boat-chair conformation of Si and P is the lowest energy conformation with the shortest dative bond, highlighting the role of dative bonding in the system’s stability.

The bond orbital data also provide a qualitative picture of the distribution of electron density among various hybrid orbitals of both the metal (M) and donor (Y) atoms comprising the dative bond (Tables S4 and S5). Participating hybrid orbitals include s-, p-, d-, and, in some cases, f-orbital contributions. The prominence of certain orbitals in these interactions provides valuable insights into the bonding mechanism. In several instances, the p-orbitals of the metal atom make a significant contribution to the dative bond’s electronic composition. This phenomenon is particularly pronounced in all compounds featuring germanium (Ge) metals where both NBO and IBO analyses show that the M hybrid is composed of up to 98% p-orbital character (Table 4). We also looked for evidence of dative interactions in the second order perturbation theory (SOPT) analysis of the Fock Matrix in the NBO basis (Table S6). These results show interactions between Y lone pair NBO orbitals and virtual M orbitals for only a subset of congeners and conformers, and suggest that As is the element most likely to donate electrons to a metal.

To further quantify the strength of the dative interaction, we calculated Wiberg bond indices (WBI) for all species (Table 4).^86,87^ WBIs are computed from the NBO natural populations and estimate the amount of electron density between two atoms. For the quasimetallatranes in this study, the WBIs between Y and M atoms range between 0.0020 (reflecting very little density) to 0.3894 (reflecting approximately 1/3 of the bond order of a single bond). In many cases, the WBIs correlate with our estimation of dative strength based on bond distances and energetic comparisons to the crown conformation (Table 1). For instance, for all congeners the crown conformations have WBIs near zero, and in most cases except for Si with P and As, the boat–boat, boat-chair and chair–chair conformers have similar WBIs. This latter WPI result is somewhat incongruent with the relative conformer energies in Table 1 which indicate that the chair–chair is ∼3–6 kcal/mol higher in energy than either the boat–boat or boat-chair conformation. The variability of all of these metrics reflects the difficulty associated with quantifying bonding.

**Figure 5:**
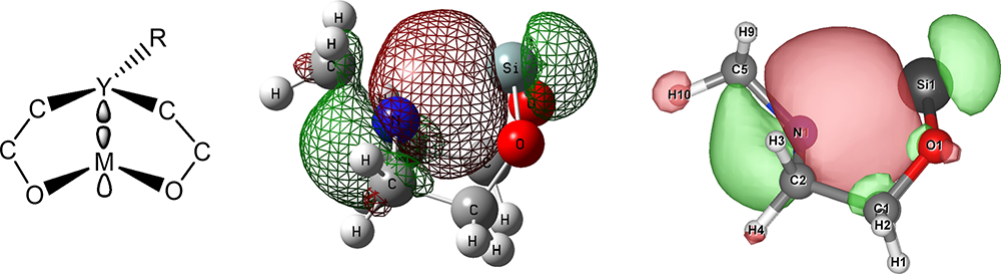
(a) Generalized orbital composition of the dative bond interaction which typically includes a p-orbital on the metal and an sp^3^ hybridized orbital on the Y atom. For some of the larger metal atoms, such as Sn and Pb, s, d and f orbital character mixes with the otherwise dominant p-orbital contribution (Table 4). (b) Natural Bond Orbital (NBO#21) representation of the dative interaction in the boat–boat conformation of the Si–N molecule. (c) Intrinsic Bond Orbital (IBO#36) representation of the dative interaction in the boat–boat conformation of the Si–N molecule. For image rendering of (b) and (c), an isovalue of 0.02 atomic units was used.

Despite the significant p-orbital character, the IBO and NBO analyses revealed no evidence of π-bonding. In almost all cases where a dative bond was identified in our orbital results, there was only one bonding orbital between the metal and the donor, indicative of mainly sigma overlap with an occupancy of approximately 2, reflecting a two-electron bond originating from the electron donor. Figure 5 demonstrates this orbital overlap and compares the NBO and IBO representations of the interaction for Y = N and M = Si. The dative bond orbital composition (i.e., %s, %p, etc.) is different in the boat–boat, boat-chair, and chair–chair conformations, emphasizing the sensitivity of the dative bond to molecular geometry. Such conformational sensitivity could influence the compound’s reactivity, especially in catalytic processes where subtle structural changes can significantly impact reaction pathways.

### Comparing Results Obtained with and without Effective Core Potentials (ECPs)

Quantum calculations on systems as large as those included in this study are computationally demanding. For this reason, and to better describe the relativistic effects^88^ in atoms with a large number of electrons, effective core potentials (ECPs) are often used to model the inner core electrons.^61,89,90^ For Sn- and Pb-containing quasimetallatranes, we compared the results obtained using the MDF ECPs^63^ (above) with those obtained using the Los Alamos ECP with the corresponding double-ζ basis sets LanL2DZ.^91−93^ We find that the conformational preferences, M ← Y dative interaction distances and near–IR M ← Y vibrational frequencies for these quasimetallatranes are relatively independent of which ECP was utilized (Table S7).

## Conclusions

We have extensively investigated the structure, stability, and bonding properties of fused ring [3.3.0] bicyclic quasimetallatranes. We have characterized the conformational flexibility of the five-membered bicyclic systems and identified the boat–boat, boat-chair, chair–chair, and crown conformations as minimum energy structures. We have confirmed the presence of dative bonds between the Group 14 metalloid or metal centers and the Group 15 electron-donors in all conformations except the highly symmetric crown conformation. For all congeners studied, the boat–boat and boat-chair are the most stable and are most likely to have the shortest dative bonding interactions. For these low energy conformations, a canonical molecular orbital analysis, along with a natural bond orbital analysis supports the existence of dative bonding. Our findings also reveal conformational diversity correlated with the size of the electron-donating group and the coordinating metal. Notably, the covalency ratios calculated align with previous studies, validating our methodological approach. This research contributes to the understanding of the unique properties of quasimetallatranes and their potential applications, and lays the foundation for future advancements in this exciting area of research.

## Supporting Information Available

Additional computational details including Cartesian coordinates, absolute energies and images for all geometry optimized species, dative bond distances for various R groups, molecular, natural bond and intrinsic orbital information, second order perturbation theory analysis in the Fock matrix of the NBO basis, and comparison of data with and without ECPs (PDF)
