# Supplementary material for: Dative Bonding in Quasimetallatranes Containing Group 15 Donors (Y = N, P, and As) and Group 14 Acceptors (M = Si, Ge, Sn, and Pb)
Source: Inorg Chem. 2024 Sep 20;63(39):18005–15. doi: 10.1021/acs.inorgchem.4c02532 (PMC11445729; doi:10.1021/acs.inorgchem.4c02532)
Supplement: Supplementary file 1 — ic4c02532_si_001.pdf [file ic4c02532_si_001.pdf]

Supporting Information for

Dative Bonding in Quasimetallatranes containing Group 15 Donors (Y = N, P and As) and Group 14 Acceptors (M = Si, Ge, Sn, and Pb)

Aamy A. Bakry, Matthew G. Fanelli, Martel Zeldin, Kelling J. Donald, and Carol A. Parish*

Department of Chemistry, Gottwald Center for the Sciences, University of Richmond, Richmond, VA 23173

*Corresponding author
cparish@richmond.edu

Table S1: The following table provides information on the optimized output for each compound analyzed in this project. The first column lists details such as the file name associated with the compound, which typically includes information about the conformation, metal, electron donor, and R group if applicable. All calculations were performed using Gaussian 16 with the B3LYP/6-31G* method for compounds containing silicon and germanium, and the B3LYP/LanL2DZ method for compounds containing tin and lead. These structures were further optimized using energy-consistent pseudopotentials (ECPs): MDF quality basis set for compounds with tin, and lead. The absolute energy of the optimized structure is reported in atomic units (au), along with the XYZ coordinates of the optimized structure. The second column contains a Gaussview PNG image of the optimized structure with atomic labels. Hydrogen atoms are not shown unless they are part of the R group or included in the R group on the metal.

File Information	Structure in Gaussview

File name: boat-boat_Si_N.log
Optimized Absolute E: -691.882250321
Optimized XYZ coordinates:
C 0.551366 -1.607053 0.982526
H 0.952910 -1.034182 1.830214
H 0.614573 -2.668737 1.256508
C -0.919231 -1.247878 0.710475
H -1.359528 -2.044321 0.104357
H -1.504964 -1.159093 1.635879
C -0.919372 1.247723 0.710547
H -1.359801 2.044148 0.104504
H -1.505054 1.158788 1.635968
C 0.551188 1.607074 0.982562
H 0.952841 1.034217 1.830207
H 0.614264 2.668754 1.256591
O 1.284685 -1.376366 -0.195841
O 1.284494 1.376541 -0.195845
C -2.167491 -0.000084 -0.992039
H -2.132249 -0.885289 -1.631410
H -3.111608 -0.000096 -0.429573
H -2.132291 0.885097 -1.631444
Si 0.986176 0.000065 -1.181218
N -0.995394 -0.000042 -0.093671

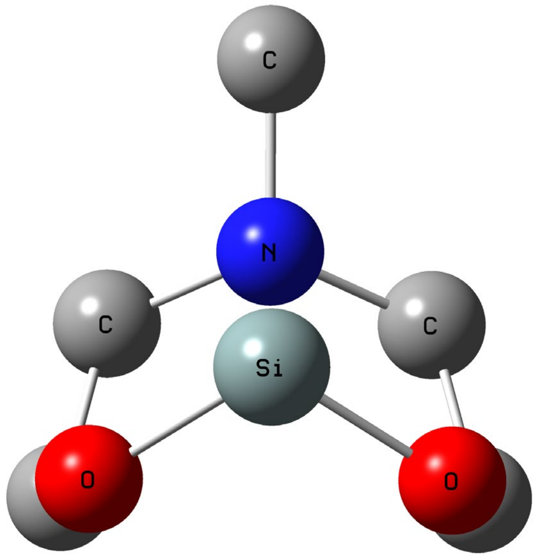

File name: boat-boat_Si_P.log
Optimized Absolute E: -978.486528980
Optimized XYZ coordinates:
C 0.613820 -1.652288 0.989130
H 0.848489 -1.026858 1.858431
H 0.790095 -2.692330 1.288305
C -0.864238 -1.474838 0.593678
H -1.195923 -2.344573 0.012280
H -1.482729 -1.430939 1.500010
C -0.864278 1.474840 0.593665
H -1.195936 2.344572 0.012246
H -1.482793 1.430956 1.499981
C 0.613770 1.652277 0.989149
H 0.848425 1.026813 1.858430
H 0.790047 2.692307 1.288364
O 1.504386 -1.364183 -0.079603
O 1.504340 1.364205 -0.079590
C -3.018789 -0.000029 -0.594631
H -3.358901 -0.883821 -1.143988

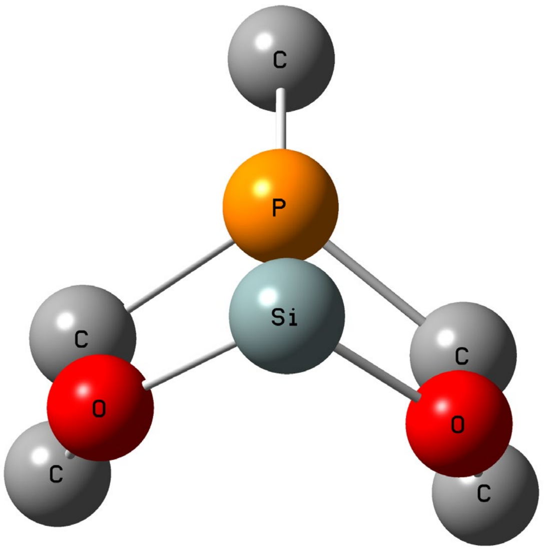

H -3.485135 -0.000035 0.397283<br>H -3.358902 0.883774 -1.143973<br>Si 1.906155 0.000022 -0.978514<br>P -1.156961 -0.000008 -0.525372	
File name: boat-boat_Si_As.log<br>Optimized Absolute E: -2870.90489975<br>Optimized XYZ coordinates:<br>C 0.891367 -1.659435 1.056641<br>H 1.164006 -1.020077 1.903651<br>H 1.115753 -2.691701 1.350897<br>C -0.603526 -1.517702 0.740203<br>H -0.953928 -2.402237 0.197183<br>H -1.185702 -1.428432 1.664014<br>C -0.603535 1.517707 0.740198<br>H -0.953927 2.402241 0.197170<br>H -1.185718 1.428445 1.664005<br>C 0.891357 1.659432 1.056645<br>H 1.163988 1.020066 1.903651<br>H 1.115747 2.691696 1.350910<br>O 1.717934 -1.362631 -0.067167<br>O 1.717926 1.362634 -0.067162<br>C -2.971027 -0.000007 -0.200876<br>H -3.390851 -0.884861 -0.685883<br>H -3.225780 -0.000009 0.862148<br>H -3.390856 0.884845 -0.685881<br>As -1.010043 -0.000001 -0.475059<br>Si 2.138855 0.000004 -0.951939	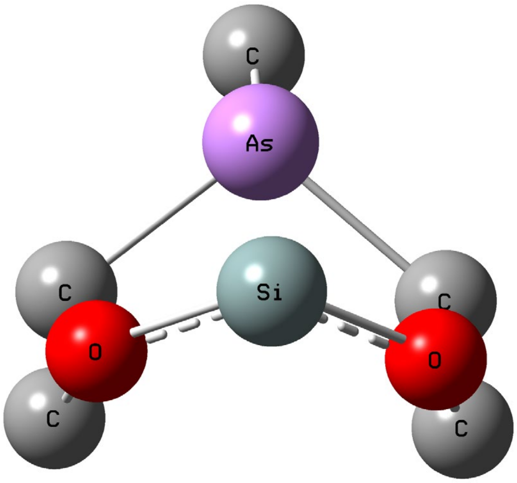
File name: boat-boat_Ge_N.log<br>Optimized Absolute E: -2477.38198265<br>Optimized XYZ coordinates:<br>C 0.344128 1.750329 1.105264<br>H 0.361797 1.312144 2.116743<br>H 0.507580 2.831987 1.229884<br>C 1.496240 1.185905 0.262114<br>H 1.627279 1.827143 -0.615322<br>H 2.446127 1.155089 0.815333<br>C 1.171400 -1.237775 0.782842<br>H 1.408261 -2.168796 0.261499<br>H 1.967457 -1.042308 1.513842<br>C -0.209905 -1.404503 1.449576<br>H -0.374577 -0.630360 2.211643<br>H -0.228919 -2.376039 1.965628	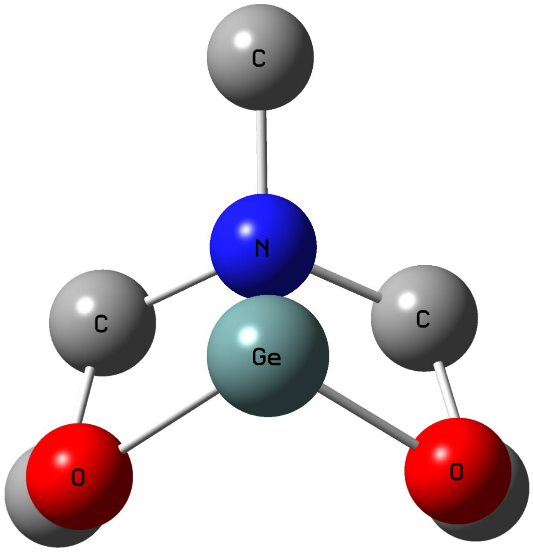

O -0.872487 1.518294 0.454714<br>O -1.196435 -1.368974 0.455429<br>C 1.915374 -0.519406 -1.449923<br>H 1.790903 0.258351 -2.207799<br>H 2.984109 -0.633293 -1.222246<br>H 1.533055 -1.457577 -1.859313<br>N 1.134846 -0.159964 -0.249097<br>Ge -1.053720 0.068798 -0.707706	
File name: boat-boat_Ge_P.log<br>Optimized Absolute E: -2763.98575734<br>Optimized XYZ coordinates:<br>C 0.204315 -1.700373 1.309090<br>H 0.337262 -1.054919 2.189965<br>H 0.291603 -2.738131 1.662167<br>C -1.220077 -1.493368 0.738464<br>H -1.482681 -2.339008 0.089927<br>H -1.965556 -1.437545 1.541353<br>C -1.220098 1.493367 0.738449<br>H -1.482715 2.338996 0.089904<br>H -1.965576 1.437540 1.541339<br>C 0.204291 1.700396 1.309073<br>H 0.337239 1.054967 2.189966<br>H 0.291570 2.738164 1.662123<br>O 1.203721 -1.472544 0.349047<br>O 1.203704 1.472550 0.349041<br>C -2.912229 -0.000020 -1.153317<br>H -3.011639 -0.885991 -1.787657<br>H -3.720526 -0.000027 -0.414361<br>H -3.011655 0.885948 -1.787658<br>Ge 1.387401 0.000001 -0.775116<br>P -1.240717 -0.000006 -0.360574	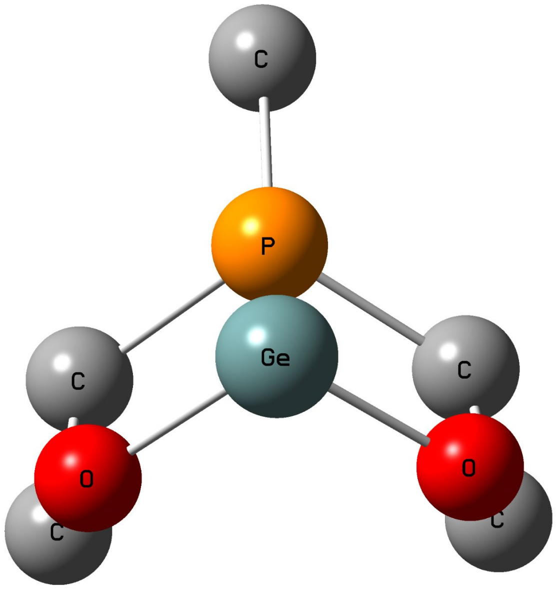

File name: boat-boat_Ge_As.log
Optimized Absolute E: -4656.40232513
Optimized XYZ coordinates:
C 0.467491 -1.701305 1.361014
H 0.645344 -1.039712 2.220427
H 0.610580 -2.730610 1.719919
C -0.986633 -1.535656 0.877132
H -1.285896 -2.402208 0.277602
H -1.688478 -1.424162 1.710222
C -0.986661 1.535677 0.877092
H -1.285903 2.402214 0.277531
H -1.688525 1.424211 1.710170
C 0.467453 1.701326 1.361002
H 0.645283 1.039747 2.220430
H 0.610543 2.730636 1.719893
O 1.408117 -1.468835 0.335901
O 1.408097 1.468833 0.335910
C -3.009881 -0.000029 -0.830426
H -3.217858 -0.888324 -1.430478
H -3.627255 -0.000017 0.070627
H -3.217869 0.888243 -1.430507
Ge 1.616736 0.000004 -0.773561
As -1.105329 -0.000006 -0.350341

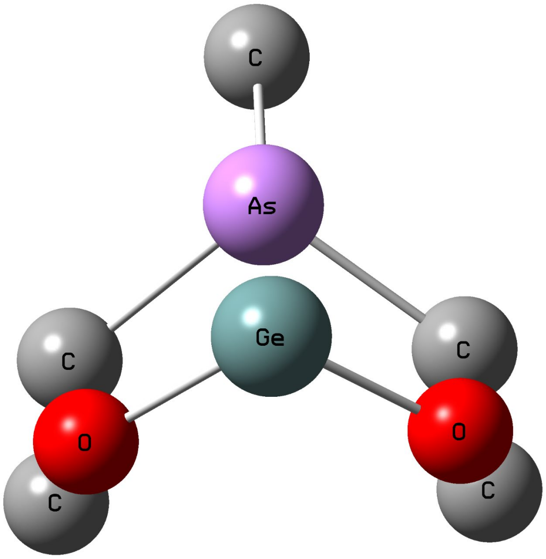

File name: boat-boat_Sn_N_ECP.log
Optimized Absolute E: -616.774849074
Optimized XYZ coordinates:
C 0.650787 -1.643410 -1.325810
H 0.759565 -1.021200 -2.227106
H 0.875638 -2.677004 -1.633544
C 1.697687 -1.249529 -0.266344
H 1.730693 -2.048116 0.481411
H 2.701423 -1.159744 -0.709634
C 1.697787 1.249517 -0.266005
H 1.730635 2.047942 0.481933
H 2.701606 1.159858 -0.709135
C 0.651069 1.643582 -1.325576
H 0.760061 1.021574 -2.226989
H 0.875932 2.677253 -1.633043
O -0.643629 -1.573564 -0.797345
O -0.643447 1.573557 -0.797389
C 1.918813 -0.000288 1.818508
H 1.582092 -0.888063 2.360740

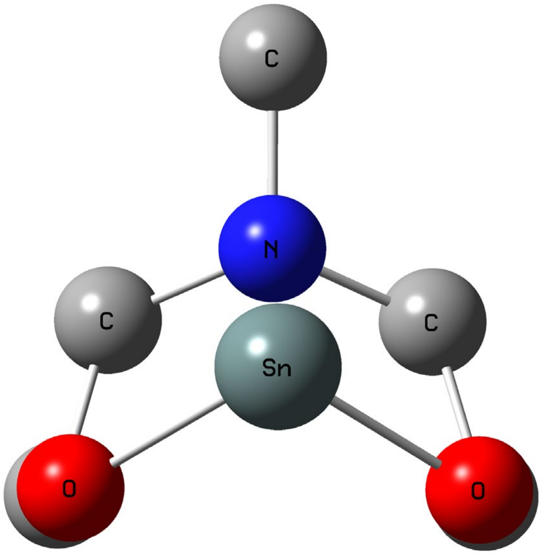

H 3.018681 -0.000339 1.792799<br>H 1.582181 0.887383 2.360965<br>Sn -1.141992 0.000038 0.387906<br>N 1.340120 -0.000086 0.459360	
File name: boat-boat_Sn_P_ECP.log<br>Optimized Absolute E: -903.381980913<br>Optimized XYZ coordinates:<br>C -0.298146 -1.737843 1.443857<br>H -0.294059 -1.072072 2.321124<br>H -0.329234 -2.766563 1.833756<br>C -1.600592 -1.496793 0.638284<br>H -1.769724 -2.339181 -0.045249<br>H -2.468174 -1.427134 1.307342<br>C -1.600618 1.496806 0.638239<br>H -1.769745 2.339176 -0.045317<br>H -2.468208 1.427160 1.307288<br>C -0.298183 1.737877 1.443826<br>H -0.294117 1.072136 2.321115<br>H -0.329271 2.766611 1.833689<br>O 0.864206 -1.581275 0.674768<br>O 0.864181 1.581275 0.674763<br>C -2.977670 -0.000041 -1.481393<br>H -2.984853 -0.886408 -2.123119<br>H -3.884254 -0.000044 -0.866607<br>H -2.984877 0.886306 -2.123146<br>Sn 1.360688 0.000002 -0.517911<br>P -1.442248 -0.000008 -0.447896	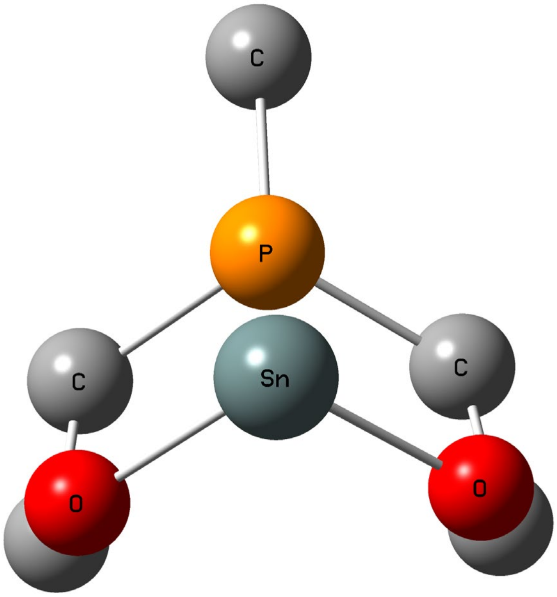

File name: boat-boat_Sn_As_ECP.log
Optimized Absolute E: -2795.43712739
Optimized XYZ coordinates:
C 0.002726 -1.750331 1.509730
H 0.065681 -1.067227 2.370447
H 0.043602 -2.771379 1.918618
C -1.363730 -1.562904 0.818400
H -1.586810 -2.423134 0.176058
H -2.169579 -1.467041 1.555483
C -1.363955 1.563045 0.818046
H -1.587037 2.423115 0.175490
H -2.169863 1.467296 1.555079
C 0.002426 1.750700 1.509455
H 0.065241 1.067937 2.370456
H 0.043284 2.771909 1.917942
O 1.098147 -1.579168 0.643386
O 1.097962 1.579149 0.643336
C -3.178467 -0.000278 -1.107377
H -3.323731 -0.889252 -1.726018
H -3.905390 -0.000252 -0.290737
H -3.323875 0.888543 -1.726204
Sn 1.594445 0.000006 -0.533136
As -1.334444 -0.000062 -0.400701

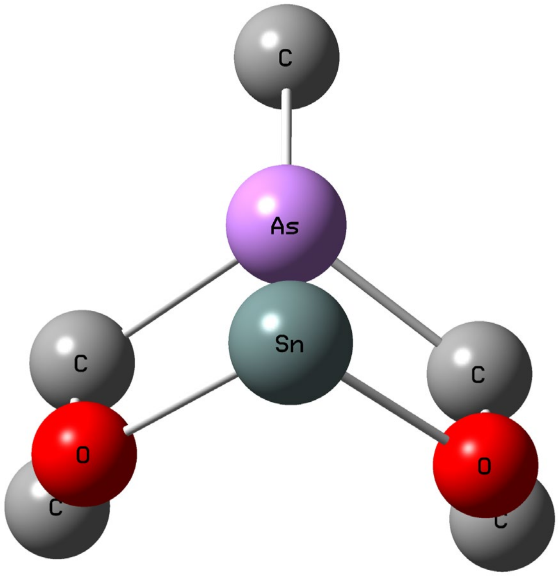

File name: boat-boat_Pb_N_ECP.log
Optimized Absolute E: -595.326300463
Optimized XYZ coordinates:
C 1.103297 -1.656009 -1.313271
H 1.294201 -1.014684 -2.188123
H 1.410223 -2.674217 -1.608186
C 2.019981 -1.249286 -0.141692
H 1.971095 -2.048974 0.604654
H 3.067038 -1.163287 -0.474554
C 2.020152 1.249291 -0.140634
H 1.970592 2.048497 0.606190
H 3.067447 1.163755 -0.472883
C 1.104125 1.656458 -1.312543
H 1.295885 1.015746 -2.187666
H 1.410945 2.674947 -1.606606
O -0.242368 -1.651489 -0.942644
O -0.241800 1.651271 -0.942892
C 2.050496 -0.000852 1.949110
H 1.669026 -0.889494 2.460142

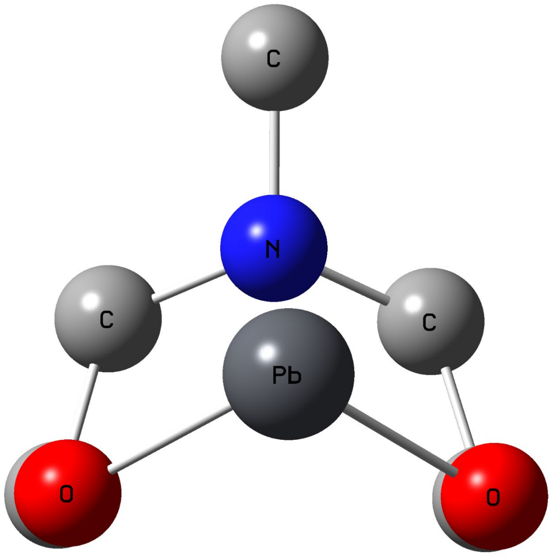

<table>
<tr><td>H 3.149001 -0.001012 2.025242
H 1.669245 0.887486 2.460838
Pb -0.964246 0.000087 0.212254
N 1.596932 -0.000248 0.544944</td><td></td></tr>
<tr><td>File name: boat-boat_Pb_P_ECP.log
Optimized Absolute E: -881.936231573
Optimized XYZ coordinates:
C -0.780641 -1.756833 1.504599
H -0.857878 -1.070164 2.363677
H -0.901122 -2.773909 1.912692
C -1.980869 -1.498820 0.555661
H -2.077336 -2.339340 -0.144376
H -2.918214 -1.424799 1.123257
C -1.980997 1.498893 0.555334
H -2.077387 2.339272 -0.144885
H -2.918409 1.424987 1.122835
C -0.780885 1.757077 1.504368
H -0.858316 1.070655 2.363629
H -0.901341 2.774278 1.912158
O 0.463341 -1.663727 0.874280
O 0.463190 1.663685 0.874281
C -3.141595 -0.000258 -1.681501
H -3.089479 -0.886916 -2.320799
H -4.101474 -0.000256 -1.153505
H -3.089574 0.886275 -2.320980
Pb 1.146459 0.000011 -0.313730
P -1.709428 -0.000066 -0.507139</td><td>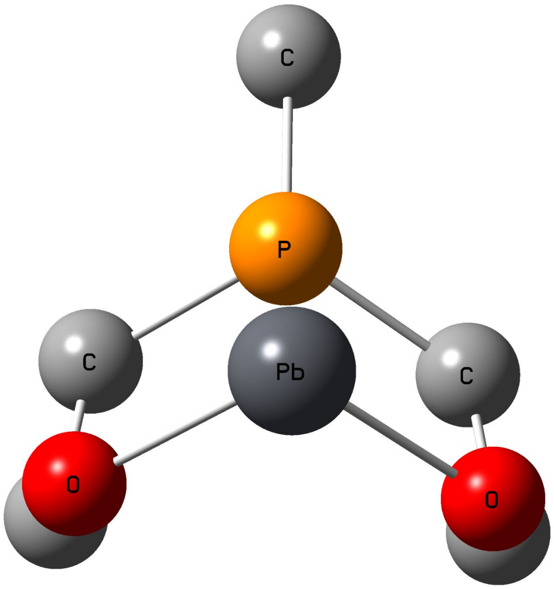
</td></tr>
<tr><td>File name: boat-boat_Pb_As_ECP.log
Optimized Absolute E: -2773.99178314
Optimized XYZ coordinates:
C -0.477227 -1.775386 1.601739
H -0.488061 -1.074843 2.452663
H -0.522331 -2.787510 2.037095
C -1.763679 -1.568033 0.772428
H -1.925398 -2.422859 0.104670
H -2.641806 -1.468501 1.421785
C -1.763677 1.568030 0.772438
H -1.925398 2.422860 0.104688
H -2.641801 1.468492 1.421798
C -0.477221 1.775377 1.601745
H -0.488048 1.074824 2.452662
H -0.522324 2.787495 2.037113</td><td>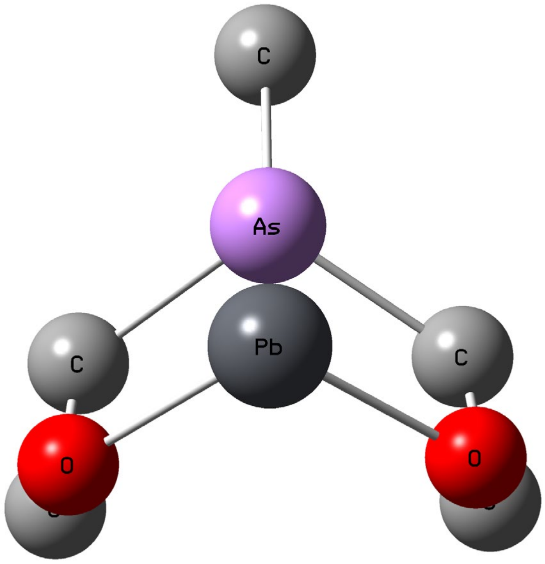
</td></tr>
</table>

O 0.703522 -1.663657 0.855769<br>O 0.703524 1.663659 0.855767<br>C -3.362571 0.000003 -1.331737<br>H -3.441031 -0.889005 -1.962383<br>H -4.173284 0.000005 -0.598126<br>H -3.441030 0.889010 -1.962385<br>Pb 1.354226 -0.000000 -0.335873<br>As -1.606853 0.000003 -0.429089	
File name: boat-chair_Si_N.log<br>Optimized Absolute E: -691.881409323<br>Optimized XYZ coordinates:<br>C -1.732135 0.392270 0.911815<br>H -1.364643 -0.184701 1.772403<br>H -2.692744 0.840361 1.194284<br>C -0.737219 1.495907 0.526674<br>H -1.200559 2.134196 -0.231641<br>H -0.453680 2.123631 1.383200<br>C 1.390667 0.275895 0.908516<br>H 2.317054 0.861516 0.956810<br>H 0.917377 0.309946 1.893529<br>O -1.907966 -0.434381 -0.220090<br>C 1.136522 1.734355 -1.061212<br>H 0.442679 1.996918 -1.864029<br>H 1.510018 2.653374 -0.587957<br>H 1.978443 1.194774 -1.502831<br>Si -0.501071 -0.973874 -1.026431<br>N 0.453439 0.861022 -0.091366<br>O 0.383759 -1.765106 0.237537<br>C 1.633484 -1.190936 0.522591<br>H 2.318271 -1.242352 -0.342670<br>H 2.114445 -1.729626 1.348616	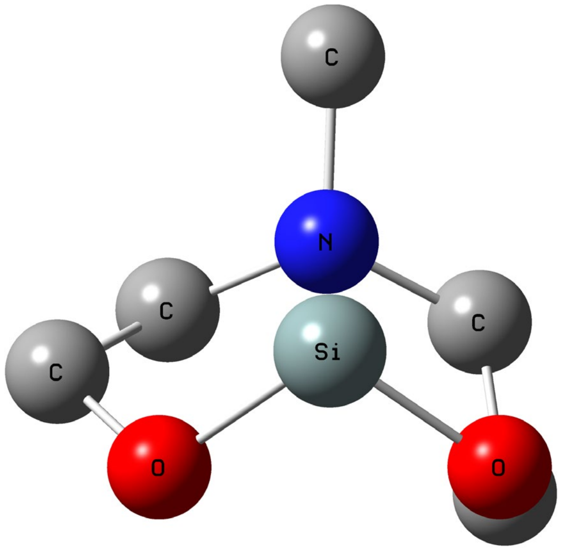

File name: boat-chair_Si_P.log<br>Optimized Absolute E: -978.487865171<br>Optimized XYZ coordinates:<br>C 0.825542 -1.597909 0.955104<br>H 1.048950 -0.863910 1.736284<br>H 1.070912 -2.593742 1.341020<br>C -0.668286 -1.541557 0.593099<br>H -0.930168 -2.415966 -0.016559<br>H -1.270075 -1.583444 1.510783<br>C -0.975540 1.402331 0.719171<br>H -1.819328 2.084718 0.556718<br>H -1.006105 1.075293 1.765587<br>O 1.651751 -1.366317 -0.185232<br>C -2.974752 -0.283695 -0.570522<br>H -3.190067 -1.194417 -1.138508<br>H -3.457246 -0.359262 0.410485<br>H -3.409412 0.560884 -1.114354<br>Si 1.804418 0.132593 -0.938017<br>O 1.397744 1.147666 0.371136<br>C 0.357791 2.107856 0.416415<br>H 0.280139 2.652706 -0.536828<br>H 0.599761 2.838672 1.196885<br>P -1.130913 -0.055386 -0.449740	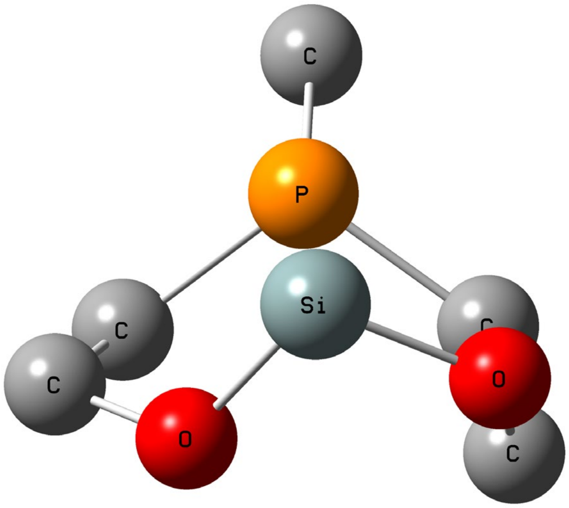
File name: boat-chair_Si_As.log<br>Optimized Absolute E: -2870.90589242<br>Optimized XYZ coordinates:<br>C 1.044175 -1.608818 1.014281<br>H 1.294236 -0.858987 1.771416<br>H 1.315300 -2.595572 1.407064<br>C -0.454676 -1.564895 0.700616<br>H -0.740610 -2.449756 0.121101<br>H -1.039431 -1.553521 1.627524<br>C -0.687773 1.470438 0.836223<br>H -1.502312 2.194164 0.717962<br>H -0.697430 1.095441 1.864853<br>O 1.834224 -1.394858 -0.160896<br>C -2.949258 -0.223363 -0.186314<br>H -3.274780 -1.146506 -0.671994<br>H -3.204157 -0.248396 0.876209<br>H -3.451354 0.616281 -0.672804<br>Si 2.075140 0.096710 -0.893789<br>O 1.661743 1.120076 0.393901	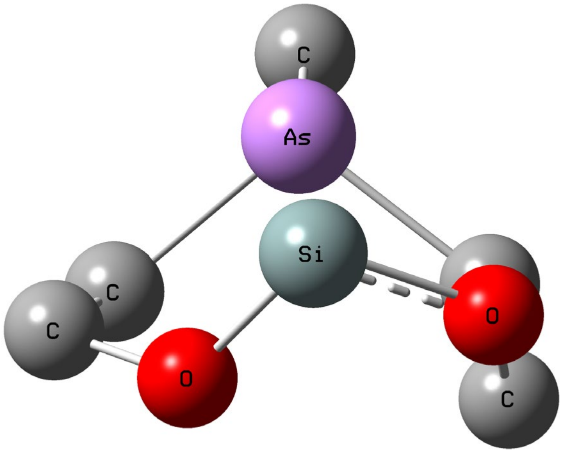

C 0.655367 2.118236 0.485713<br>H 0.563031 2.667777 -0.463257<br>H 0.965572 2.834944 1.255492<br>As -0.996811 -0.026095 -0.432960	
File name: boat-chair_Ge_N.log<br>Optimized Absolute E: -2477.38220664<br>Optimized XYZ coordinates:<br>C -0.575181 1.646635 1.134060<br>H -0.466472 1.024353 2.034862<br>H -0.875112 2.653580 1.456946<br>C 0.759639 1.738118 0.381080<br>H 0.632688 2.432567 -0.455040<br>H 1.581888 2.097641 1.015948<br>C 1.638982 -0.547697 0.810709<br>H 2.704873 -0.722538 0.617061<br>H 1.541496 -0.097422 1.801629<br>O -1.540795 1.119313 0.258828<br>C 1.910655 0.488056 -1.411342<br>H 1.379459 1.065743 -2.172759<br>H 2.883559 0.957696 -1.210757<br>H 2.074058 -0.518555 -1.805432<br>O -0.547666 -1.483908 0.831278<br>C 0.804942 -1.838134 0.773841<br>H 1.055305 -2.408668 -0.141756<br>H 1.068570 -2.476469 1.628911<br>Ge -0.990277 -0.401653 -0.632982<br>N 1.083147 0.405695 -0.195019	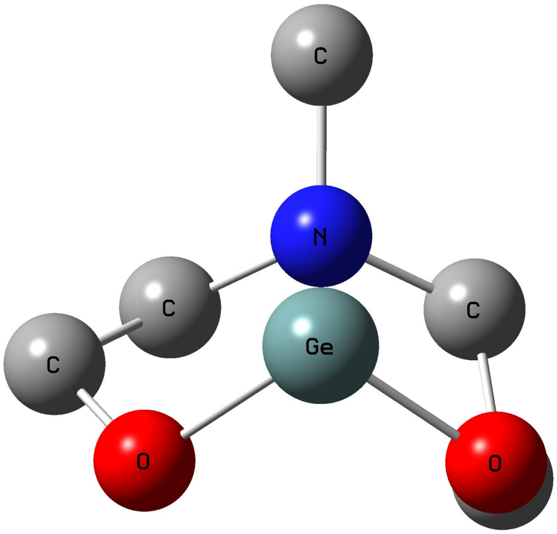
File name: boat-chair_Ge_P.log<br>Optimized Absolute E: -2763.98836191<br>Optimized XYZ coordinates:<br>C -0.426897 1.657982 1.246989<br>H -0.582763 0.898969 2.023206<br>H -0.592737 2.646212 1.696687<br>C 1.028868 1.577725 0.737936<br>H 1.235707 2.440184 0.091656<br>H 1.741919 1.594844 1.571942<br>C 1.367256 -1.387926 0.819786<br>H 2.206497 -2.034106 0.534558<br>H 1.533421 -1.048825 1.848515<br>O -1.346893 1.494258 0.187806<br>C 2.954938 0.279845 -1.051847<br>H 2.966734 1.159606 -1.702471	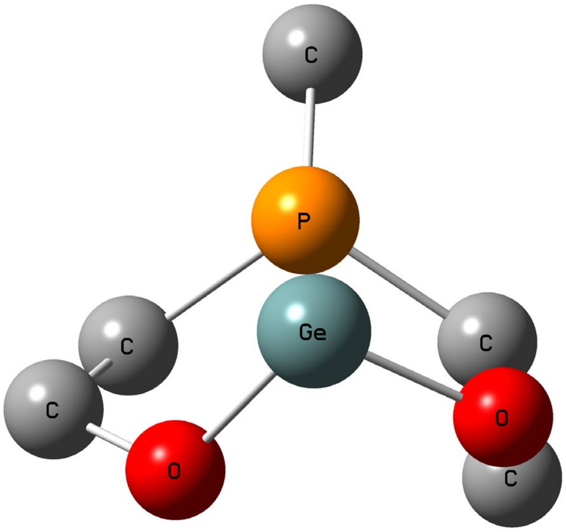

H 3.721361 0.401661 -0.278920<br>H 3.200654 -0.596203 -1.659767<br>O -1.038478 -1.185484 0.779114<br>C 0.008989 -2.119518 0.710221<br>H -0.007716 -2.694455 -0.232531<br>H -0.101552 -2.841122 1.530930<br>Ge -1.398469 -0.110939 -0.718227<br>P 1.260902 0.073631 -0.330294	
File name: boat-chair_Ge_As.log<br>Optimized Absolute E: -4656.40549405<br>Optimized XYZ coordinates:<br>C 0.650131 -1.643814 1.302357<br>H 0.848354 -0.862132 2.044416<br>H 0.849793 -2.617911 1.768795<br>C -0.823179 -1.581153 0.866638<br>H -1.074749 -2.463510 0.268121<br>H -1.503582 -1.527503 1.723583<br>C -1.100767 1.482966 0.932502<br>H -1.917721 2.171377 0.690943<br>H -1.237787 1.108554 1.951358<br>O 1.521877 -1.512465 0.191142<br>C -3.034844 -0.238634 -0.748585<br>H -3.171630 -1.148090 -1.337513<br>H -3.617730 -0.295424 0.173603<br>H -3.354215 0.619348 -1.343834<br>O 1.288749 1.157702 0.772371<br>C 0.278401 2.142048 0.755393<br>H 0.279357 2.716145 -0.187284<br>H 0.475804 2.853844 1.568504<br>As -1.125889 -0.039563 -0.326808<br>Ge 1.633593 0.081891 -0.715436	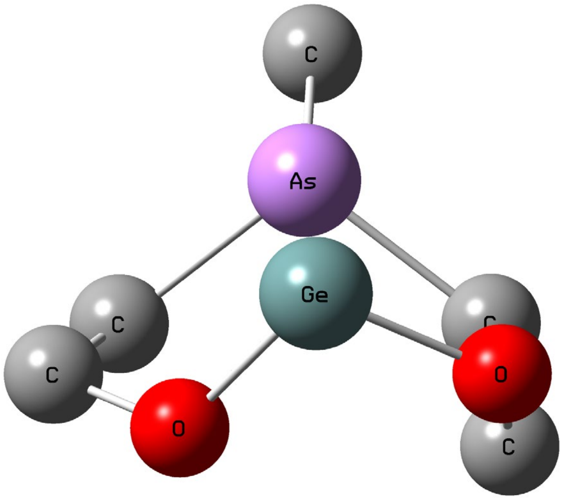

File name: boat-chair_Sn_N_ECP.log
Optimized Absolute E: -616.773326318
Optimized XYZ coordinates:
C -0.469057 1.914551 -1.096688
H -0.637942 1.396735 -2.055160
H -0.588121 2.990820 -1.293921
C -1.544269 1.502218 -0.077199
H -1.436245 2.128508 0.815044
H -2.558659 1.650344 -0.479427
C -1.778194 -0.881877 -0.702593
H -2.774741 -1.280464 -0.464119
H -1.847113 -0.348827 -1.653637
O 0.814150 1.661589 -0.592240
C -1.998449 -0.192512 1.641785
H -1.568249 0.448707 2.416845
H -3.084067 -0.019219 1.599773
H -1.823066 -1.235993 1.918692
O 0.534343 -1.438140 -1.067988
C -0.731979 -1.995184 -0.853169
H -0.758604 -2.648692 0.041300
H -1.017688 -2.629083 -1.706247
N -1.354636 0.090865 0.346998
Sn 1.118414 -0.099193 0.364818

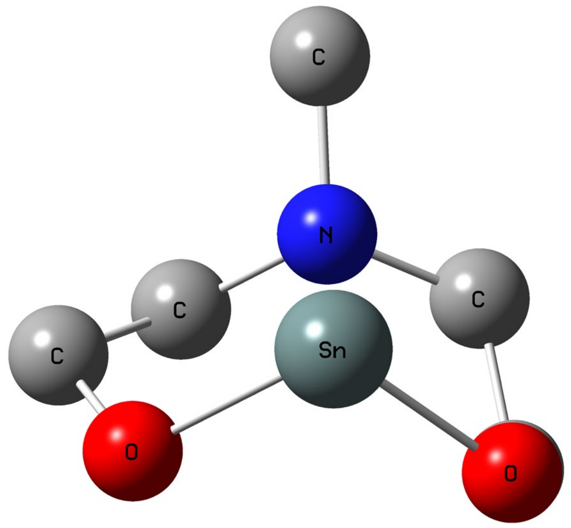

File name: boat-chair_Sn_P_ECP.log
Optimized Absolute E: -903.381594684
Optimized XYZ coordinates:
H -0.082316 -1.003094 2.182444
H -0.107726 -2.744203 1.854301
C -1.459430 -1.604314 0.616301
H -1.570781 -2.438304 -0.088307
H -2.311061 -1.626070 1.309301
C -1.700172 1.350876 0.813472
H -2.542831 1.982682 0.505371
H -1.929930 0.938976 1.802827
O 0.988073 -1.648033 0.521622
C -3.049945 -0.145078 -1.366214
H -2.998839 -0.988797 -2.061432
H -3.920709 -0.276112 -0.714669
H -3.179044 0.770830 -1.951119
O 0.725489 1.299955 1.020098
C -0.377618 2.152363 0.869205
H -0.305431 2.774725 -0.042296

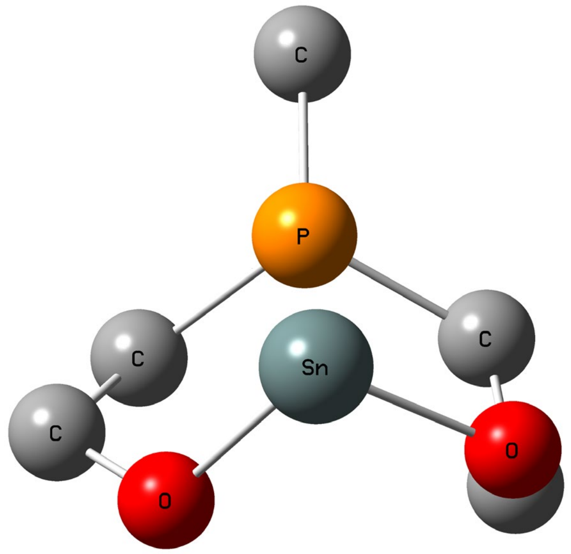

H -0.414004 2.849045 1.719443<br>Sn 1.360096 0.064926 -0.494607<br>P -1.472540 -0.047163 -0.399212<br>C -0.123257 -1.752829 1.378718	
File name: boat-chair_Sn_As_ECP.log<br>Optimized Absolute E: -2795.43778412<br>Optimized XYZ coordinates:<br>C 0.154882 -1.731985 1.450947<br>H 0.253503 -0.952358 2.219223<br>H 0.228185 -2.705191 1.958084<br>C -1.234150 -1.626201 0.797655<br>H -1.413883 -2.488843 0.145557<br>H -2.025440 -1.592731 1.556378<br>C -1.446360 1.469117 0.938806<br>H -2.268070 2.142031 0.666964<br>H -1.648789 1.056897 1.932826<br>O 1.200695 -1.649998 0.505865<br>C -3.219409 -0.164043 -1.020046<br>H -3.309259 -1.045664 -1.659591<br>H -3.916639 -0.249276 -0.182155<br>H -3.461081 0.724862 -1.607981<br>O 0.979399 1.273401 1.007667<br>C -0.084552 2.188433 0.914598<br>H -0.023633 2.803919 -0.001432<br>H -0.024495 2.885607 1.763289<br>Sn 1.600057 0.049052 -0.505473<br>As -1.359287 -0.025183 -0.367198	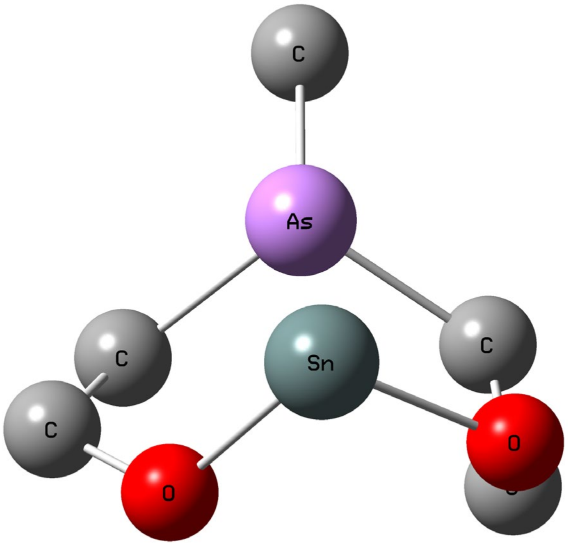
File name: boat-chair_Pb_N_ECP.log<br>Optimized Absolute E: -595.325171244<br>Optimized XYZ coordinates:<br>C -0.959274 1.935038 -1.088752<br>H -1.177461 1.400251 -2.029190<br>H -1.193702 2.996315 -1.273858<br>C -1.910728 1.447557 0.019227<br>H -1.753945 2.070637 0.907028<br>H -2.963627 1.553533 -0.289309<br>C -2.086473 -0.937670 -0.615763<br>H -3.057639 -1.360597 -0.316879<br>H -2.228209 -0.405568 -1.559273<br>O 0.379833 1.801407 -0.712496<br>C -2.157055 -0.279442 1.741935<br>H -1.701773 0.374945 2.491830	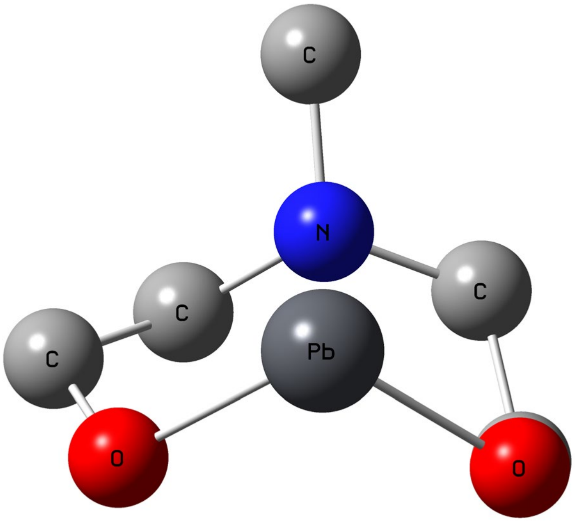

H -3.250365 -0.155929 1.785605<br>H -1.917450 -1.316823 1.993536<br>O 0.191082 -1.475755 -1.216801<br>C -1.034145 -2.032653 -0.846322<br>H -0.958958 -2.661765 0.065475<br>H -1.407619 -2.697931 -1.642153<br>Pb 0.942861 -0.042731 0.209583<br>N -1.626450 0.043523 0.407687	
File name: boat-chair_Pb_P_ECP.log<br>Optimized Absolute E: -881.936075691<br>Optimized XYZ coordinates:<br>C -0.606437 -1.800933 1.434405<br>H -0.622313 -1.044840 2.235190<br>H -0.692792 -2.785895 1.919849<br>C -1.853802 -1.608170 0.539666<br>H -1.907564 -2.429268 -0.186697<br>H -2.772951 -1.623212 1.141229<br>C -2.071062 1.348286 0.765852<br>H -2.893851 1.976957 0.401407<br>H -2.373207 0.918933 1.728026<br>O 0.589484 -1.759724 0.703819<br>C -3.235551 -0.091428 -1.548055<br>H -3.136142 -0.923749 -2.251776<br>H -4.161112 -0.220887 -0.976426<br>H -3.301851 0.836562 -2.124444<br>O 0.324621 1.340095 1.224988<br>C -0.767867 2.164638 0.943999<br>H -0.616006 2.778087 0.033660<br>H -0.910632 2.877161 1.772065<br>Pb 1.140213 0.041817 -0.308245<br>P -1.747572 -0.033745 -0.444109	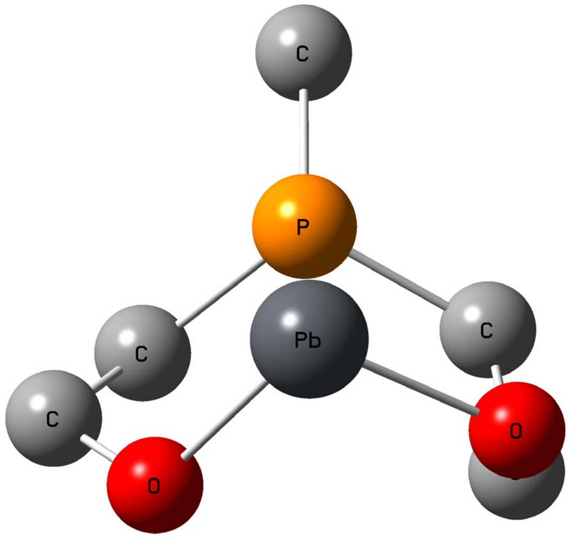

File name: boat-chair_Pb_As_ECP.log<br>Optimized Absolute E: -2773.99255100<br>Optimized XYZ coordinates:<br>H -0.273808 -0.984146 2.306005<br>H -0.337543 -2.739044 2.073322<br>C -1.639256 -1.639303 0.759055<br>H -1.765778 -2.491949 0.081740<br>H -2.500502 -1.601618 1.437551<br>C -1.839740 1.459637 0.920009<br>H -2.651687 2.120816 0.594217<br>H -2.108678 1.029624 1.890475<br>O 0.811823 -1.750166 0.706521<br>C -3.419352 -0.130590 -1.231789<br>H -3.450427 -0.999438 -1.894003<br>H -4.199245 -0.224567 -0.471196<br>H -3.593169 0.771493 -1.823720<br>O 0.577180 1.325329 1.220281<br>C -0.493705 2.204741 1.013659<br>H -0.367280 2.816860 0.099521<br>H -0.539155 2.915957 1.854037<br>As -1.636692 -0.021061 -0.387509<br>Pb 1.353095 0.033655 -0.326682<br>C -0.319230 -1.774489 1.541288	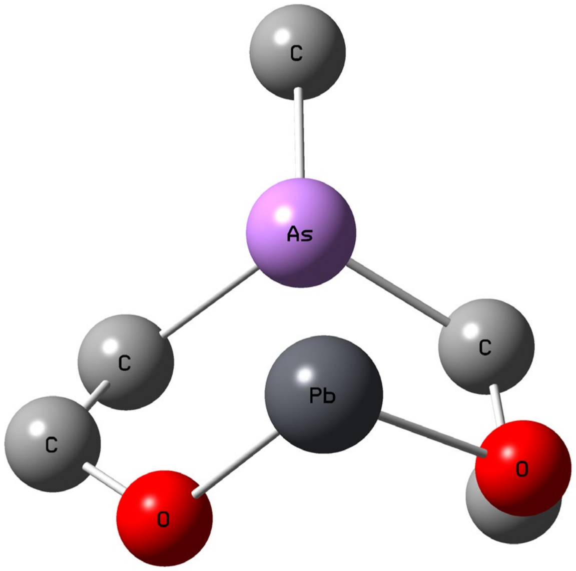
File name: chair-chair_Si_N.log<br>Optimized Absolute E: -691.871762272<br>Optimized XYZ coordinates:<br>C 2.149550 -0.208447 -0.308820<br>H 2.968932 -0.369546 -1.019935<br>H 2.608449 0.042478 0.664013<br>C 1.257721 0.951255 -0.782431<br>H 1.757010 1.924675 -0.694345<br>H 1.003191 0.790441 -1.832009<br>C -1.263562 0.954341 -0.775783<br>H -1.764713 1.925663 -0.676906<br>H -1.014469 0.803246 -1.828136<br>C -2.150222 -0.211269 -0.305933<br>H -2.971111 -0.371600 -1.015450<br>H -2.606999 0.033260 0.669556<br>O 1.353420 -1.367631 -0.228908<br>O -1.349667 -1.367866 -0.234475<br>C 0.003585 1.916570 1.111666<br>H 0.888569 1.765897 1.735469	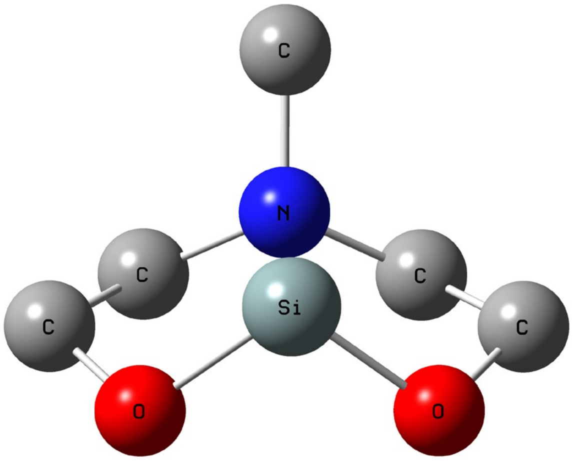

H 0.003555 2.944256 0.722925<br>H -0.877891 1.767708 1.740824<br>N -0.000792 0.927975 0.017986<br>Si -0.000103 -1.163074 0.820212	
File name: chair-chair_Si_P.log<br>Optimized Absolute E: -978.482555106<br>Optimized XYZ coordinates:<br>C 0.701142 2.045747 -0.300983<br>H 1.072550 2.658035 -1.132784<br>H 0.414461 2.731914 0.506219<br>C -0.528331 1.256077 -0.766916<br>H -1.286265 1.998423 -1.051243<br>H -0.290294 0.671832 -1.660232<br>C -1.165323 -1.544611 -0.307883<br>H -1.897296 -2.219881 0.154534<br>H -1.431741 -1.443371 -1.368116<br>C 0.239624 -2.137184 -0.172285<br>H 0.358053 -3.004560 -0.832859<br>H 0.406349 -2.480532 0.859305<br>O 1.805086 1.289728 0.192176<br>O 1.201383 -1.155207 -0.540424<br>C -3.099233 0.463137 0.243638<br>H -3.336145 1.484518 0.559959<br>H -3.384160 0.347193 -0.808998<br>H -3.705663 -0.219879 0.847858<br>P -1.287624 0.125333 0.548132<br>Si 2.246820 -0.284205 0.451209	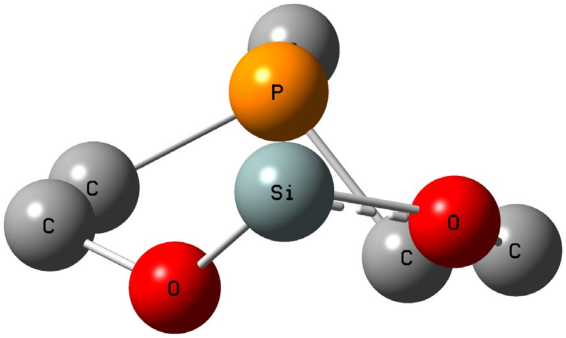
File name: chair-chair_Si_As.log<br>Optimized Absolute E: -2870.90106473<br>Optimized XYZ coordinates:<br>C -0.991562 2.037107 0.515004<br>H -1.471797 2.526957 1.371874<br>H -0.673799 2.826589 -0.178530<br>C 0.234302 1.247283 0.982348<br>H 0.999581 1.970990 1.286671<br>H -0.004725 0.611938 1.837074<br>C 0.907493 -1.640771 0.502882<br>H 1.636043 -2.326346 0.055727<br>H 1.177019 -1.475401 1.551235<br>C -0.511326 -2.198583 0.368202<br>H -0.703427 -2.985575 1.107061<br>H -0.648349 -2.640796 -0.630478	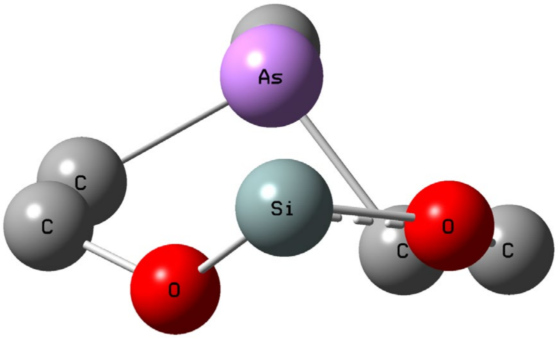

O -1.998108 1.291402 -0.173066<br>O -1.446944 -1.145280 0.589125<br>C 2.924226 0.460453 -0.052333<br>H 3.147293 1.490572 -0.342025<br>H 3.133703 0.317307 1.011267<br>H 3.551920 -0.206194 -0.649063<br>As 1.019981 0.098777 -0.453580<br>Si -2.258658 -0.283683 -0.619839	
File name: chair-chair_Ge_N.log<br>Optimized Absolute E: -2477.37298868<br>Optimized XYZ coordinates:<br>C 2.173848 0.112982 -0.465426<br>H 3.021988 0.142983 -1.163526<br>H 2.598624 0.182869 0.555047<br>C 1.254686 1.319001 -0.720905<br>H 1.734429 2.275071 -0.473185<br>H 0.991158 1.326320 -1.780005<br>C -1.269271 1.320643 -0.706497<br>H -1.755247 2.267647 -0.437673<br>H -1.016589 1.349673 -1.767921<br>C -2.175467 0.102002 -0.461023<br>H -3.026738 0.130519 -1.155310<br>H -2.596556 0.157696 0.561935<br>O 1.434082 -1.063207 -0.659630<br>O -1.424241 -1.064194 -0.670055<br>C 0.005340 1.917731 1.324446<br>H 0.891513 1.653365 1.907988<br>H 0.004513 2.999513 1.133065<br>H -0.873433 1.653706 1.919187<br>N -0.002847 1.146614 0.068193<br>Ge 0.001022 -1.055644 0.532405	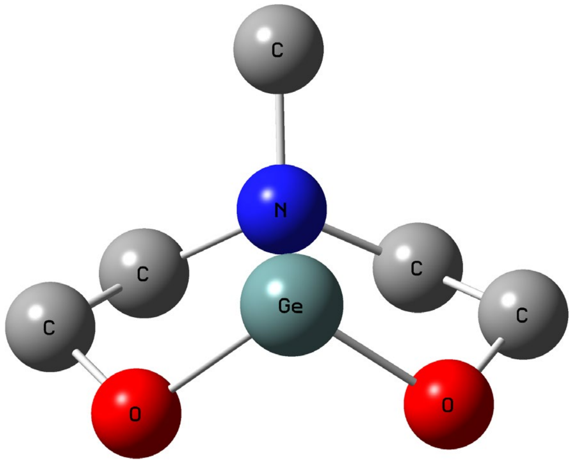

File name: chair-chair_Ge_P.log
Optimized Absolute E: -2763.98059813
Optimized XYZ coordinates:
C 0.359524 2.158806 -0.720525
H 0.661625 2.735286 -1.606909
H 0.052966 2.890712 0.047041
C -0.848944 1.279528 -1.093389
H -1.721029 1.898430 -1.337832
H -0.592622 0.664122 -1.959311
C -1.511055 -1.543706 -0.514294
H -2.228954 -2.135982 0.065853
H -1.924424 -1.397272 -1.518628
C -0.113151 -2.212296 -0.578169
H -0.090021 -2.983348 -1.359204
H 0.073718 -2.720237 0.385189
O 1.470095 1.427438 -0.256899
O 0.860938 -1.235665 -0.861458
C -2.864016 0.625621 0.992433
H -2.778222 1.638384 1.398308
H -3.650307 0.616096 0.229803
H -3.152235 -0.043851 1.808638
P -1.224961 0.099868 0.305827
Ge 1.404422 -0.188821 0.615317

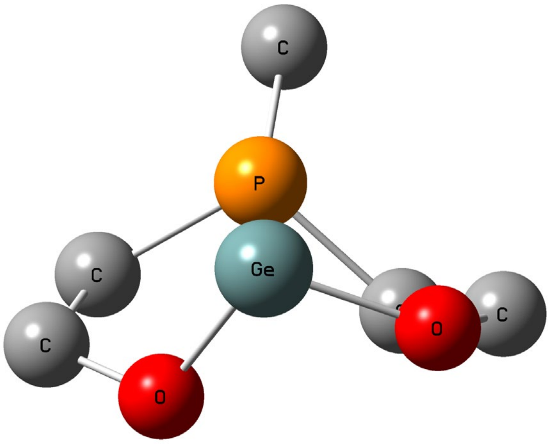

File name: chair-chair_Ge_As.log
Optimized Absolute E: -4656.39830527
Optimized XYZ coordinates:
C -0.547436 2.158069 0.787823
H -0.910854 2.698408 1.673865
H -0.196950 2.917781 0.068884
C 0.637652 1.269722 1.191813
H 1.504553 1.884770 1.456473
H 0.367858 0.627198 2.031278
C 1.251190 -1.658266 0.634459
H 1.952122 -2.311517 0.104732
H 1.633292 -1.465506 1.641325
C -0.182427 -2.227331 0.655113
H -0.296771 -2.972146 1.453649
H -0.370898 -2.746331 -0.301391
O -1.639182 1.465030 0.215993
O -1.105302 -1.179512 0.877518
C 2.964537 0.522817 -0.713505
H 2.996056 1.552023 -1.077680

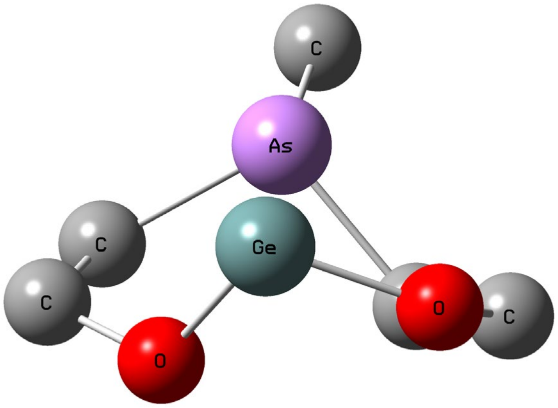

H 3.574728 0.421183 0.187195<br>H 3.336329 -0.140846 -1.496875<br>As 1.095718 0.064826 -0.311156<br>Ge -1.641668 -0.164953 -0.611113	
File name: chair-chair_Sn_N_ECP.log<br>Optimized Absolute E: -616.764308126<br>Optimized XYZ coordinates:<br>C -0.477261 2.144034 -0.787507<br>H -0.543643 2.712185 -1.728102<br>H -0.768040 2.842601 0.017299<br>C -1.478847 0.989483 -0.880501<br>H -2.514516 1.360169 -0.941144<br>H -1.257307 0.417092 -1.782585<br>C -1.796409 -1.326342 -0.127155<br>H -2.303637 -1.795328 0.722427<br>H -2.528728 -1.243204 -0.939459<br>C -0.566902 -2.182604 -0.559826<br>H -0.893694 -2.939459 -1.286843<br>H -0.215454 -2.742333 0.330816<br>O 0.835756 1.683189 -0.591941<br>O 0.445512 -1.388195 -1.111970<br>C -2.040650 0.543295 1.470496<br>H -1.655305 1.533492 1.728878<br>H -3.126561 0.621309 1.312822<br>H -1.852585 -0.129988 2.311603<br>Sn 1.100702 -0.085803 0.346201<br>N -1.352192 0.040927 0.268929	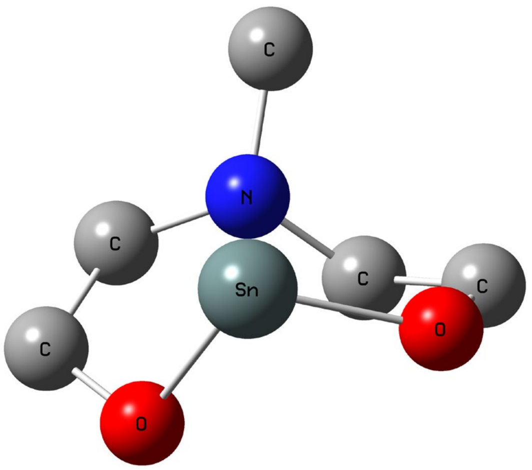
File name: chair-chair_Sn_P_ECP.log<br>Optimized Absolute E: -903.375142132<br>Optimized XYZ coordinates:<br>C -0.167119 -2.276272 0.782000<br>H -0.082045 -2.940059 1.655607<br>H -0.416503 -2.923415 -0.078799<br>C -1.335985 -1.302991 1.034632<br>H -2.283551 -1.848276 1.135694<br>H -1.151359 -0.751003 1.961120<br>C -1.764610 1.584229 0.509674<br>H -2.454163 2.192491 -0.088835<br>H -2.230604 1.403677 1.485043<br>C -0.384302 2.271948 0.677129<br>H -0.453045 3.050839 1.449678<br>H -0.139348 2.791166 -0.269221	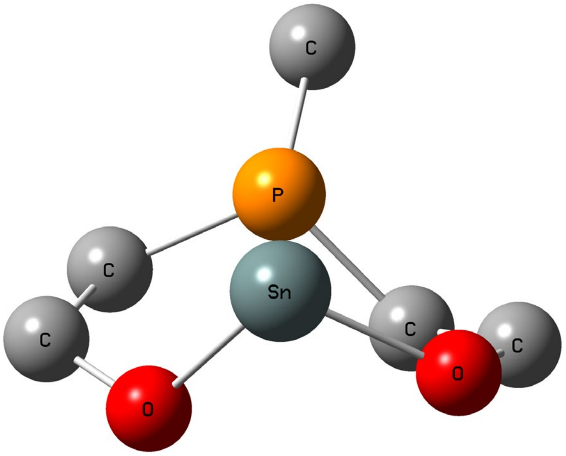

O 1.065778 -1.635364 0.577220<br>O 0.597613 1.336881 1.042974<br>C -3.016509 -0.452313 -1.246266<br>H -2.930272 -1.452312 -1.682943<br>H -3.888479 -0.430398 -0.583377<br>H -3.171240 0.262091 -2.060777<br>Sn 1.354009 0.094244 -0.426758<br>P -1.453055 -0.041782 -0.339324	
File name: chair-chair_Sn_As_ECP.log<br>Optimized Absolute E: -2795.43180280<br>Optimized XYZ coordinates:<br>C -0.086897 2.294407 0.835602<br>H -0.247643 2.936936 1.714810<br>H 0.192550 2.965426 0.004009<br>C 1.091932 1.356215 1.138365<br>H 2.016666 1.929895 1.273141<br>H 0.894103 0.781052 2.046737<br>C 1.528932 -1.667228 0.674171<br>H 2.241839 -2.322413 0.160594<br>H 1.919448 -1.439030 1.671219<br>C 0.119293 -2.287140 0.758228<br>H 0.096340 -3.052191 1.547582<br>H -0.093885 -2.811505 -0.192245<br>O -1.296580 1.638746 0.538409<br>O -0.841188 -1.299263 1.048213<br>C 3.186859 0.393238 -0.942192<br>H 3.227688 1.391638 -1.384987<br>H 3.886605 0.337331 -0.103728<br>H 3.466550 -0.341386 -1.701451<br>As 1.342226 0.032271 -0.329389<br>Sn -1.596645 -0.093870 -0.432877	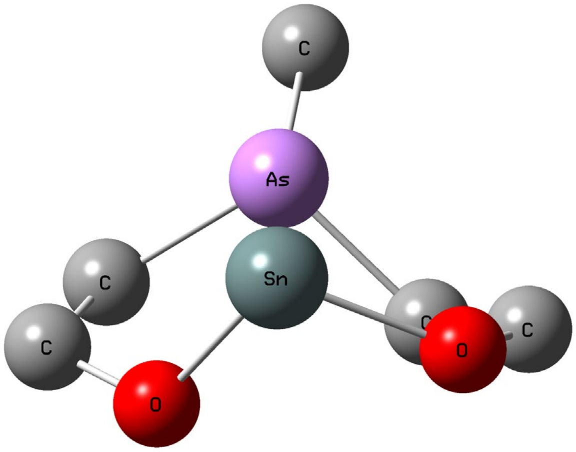

File name: chair-chair_Pb_N_ECP.log
Optimized Absolute E: -595.316500456
Optimized XYZ coordinates:
C -0.954576 2.162226 -0.758157
H -1.164855 2.753611 -1.664815
H -1.236683 2.805788 0.096374
C -1.866933 0.932749 -0.815839
H -2.928849 1.230254 -0.831898
H -1.643284 0.388575 -1.735288
C -2.038974 -1.402293 -0.081985
H -2.516850 -1.892929 0.773294
H -2.785432 -1.343283 -0.883961
C -0.799462 -2.220427 -0.541579
H -1.143302 -3.064444 -1.159939
H -0.349991 -2.677956 0.368608
O 0.404474 1.825812 -0.704668
O 0.114482 -1.435459 -1.244730
C -2.279281 0.432515 1.552431
H -1.916839 1.430668 1.813751
H -3.374851 0.476890 1.451957
H -2.030278 -0.250322 2.370122
Pb 0.927136 -0.027482 0.203655
N -1.635751 -0.022106 0.309726

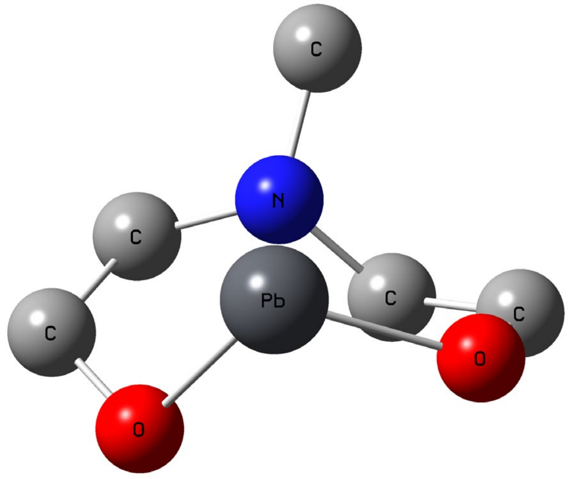

File name: chair-chair_Pb_P_ECP.log
Optimized Absolute E: -881.929997370
Optimized XYZ coordinates:
C 0.616165 2.325032 0.809012
H 0.664338 3.031844 1.653452
H 0.824146 2.919983 -0.101763
C 1.748976 1.295195 0.999585
H 2.728160 1.790921 1.043378
H 1.587299 0.758798 1.939765
C 2.086941 -1.608095 0.469167
H 2.737947 -2.219373 -0.168745
H 2.611967 -1.423903 1.413564
C 0.721525 -2.297324 0.729121
H 0.860984 -3.105909 1.463482
H 0.404541 -2.790617 -0.212631
O -0.662137 1.756786 0.763787
O -0.225657 -1.384780 1.205486
C 3.237099 0.395109 -1.385814
H 3.138200 1.394679 -1.820816

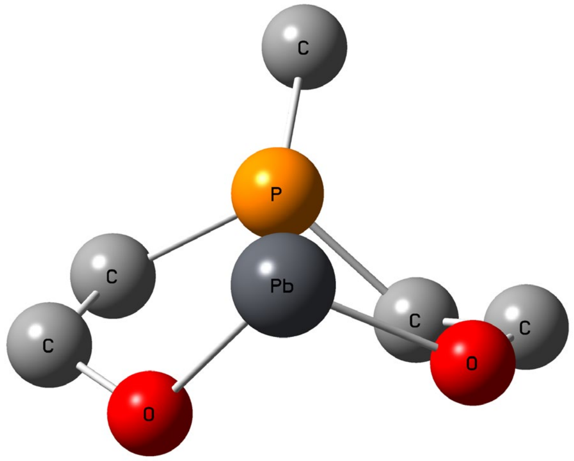

H 4.153476 0.360362 -0.786200<br>H 3.320876 -0.326100 -2.204724<br>Pb -1.127331 -0.052441 -0.271184<br>P 1.736488 0.018261 -0.364150	
File name: chair-chair_Pb_As_ECP.log<br>Optimized Absolute E: -2773.98685033<br>Optimized XYZ coordinates:<br>C -0.358372 -2.352976 0.880708<br>H -0.326292 -3.047744 1.736288<br>H -0.595002 -2.969397 -0.007627<br>C -1.516511 -1.370584 1.122672<br>H -2.473128 -1.902775 1.192161<br>H -1.348332 -0.814956 2.049435<br>C -1.877288 1.677945 0.660013<br>H -2.568183 2.326457 0.108694<br>H -2.320094 1.440123 1.632734<br>C -0.481657 2.313939 0.829975<br>H -0.534765 3.106066 1.593525<br>H -0.212274 2.820440 -0.118718<br>O 0.900494 -1.749983 0.749977<br>O 0.466965 1.357475 1.218018<br>C -3.405113 -0.352175 -1.121313<br>H -3.414046 -1.345092 -1.578047<br>H -4.178447 -0.299181 -0.349882<br>H -3.607696 0.394084 -1.893634<br>As -1.623957 -0.017413 -0.333661<br>Pb 1.342230 0.054997 -0.284517	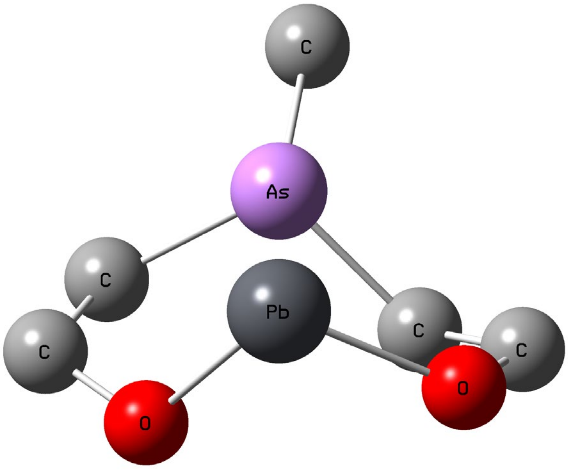
File name: crown_Si_N.log<br>Optimized Absolute E: -691.864130157<br>Optimized XYZ coordinates:<br>C -0.042521 2.026162 -0.086484<br>H -0.172471 3.014487 0.374661<br>H 0.268129 2.179868 -1.128303<br>C 1.038992 1.248008 0.660988<br>H 1.924321 1.897382 0.756623<br>H 0.683679 1.052948 1.681942<br>C 1.039178 -1.248199 0.660758<br>H 1.924569 -1.897522 0.756096<br>H 0.684003 -1.053411 1.681811<br>C -0.042469 -2.026142 -0.086721<br>H -0.172282 -3.014662 0.374030<br>H 0.267883 -2.179365 -1.128697	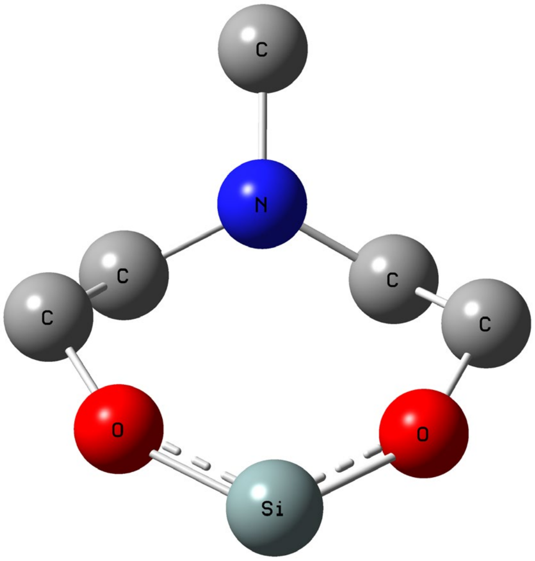

O -1.308205 1.388199 -0.078593<br>O -1.308171 -1.388189 -0.078132<br>C 2.652112 0.000158 -0.709694<br>H 2.720356 0.881057 -1.357281<br>H 3.535115 0.000222 -0.040967<br>H 2.720532 -0.880676 -1.357348<br>N 1.386213 0.000002 0.000244<br>Si -2.216291 -0.000024 -0.142397	
File name: crown_Si_P.log<br>Optimized Absolute E: -978.483089761<br>Optimized XYZ coordinates:<br>C 0.470751 -1.914350 -0.525820<br>H 0.585242 -3.002417 -0.459271<br>H 0.416589 -1.657857 -1.592467<br>C -0.816873 -1.493893 0.199127<br>H -1.545402 -2.309025 0.089209<br>H -0.614533 -1.397126 1.273963<br>C -0.816928 1.493920 0.199261<br>H -1.545450 2.309072 0.089457<br>H -0.614501 1.397069 1.274071<br>C 0.470643 1.914397 -0.525793<br>H 0.585192 3.002454 -0.459190<br>H 0.416361 1.657958 -1.592445<br>O 1.649948 -1.361457 0.040193<br>O 1.649861 1.361410 0.040069<br>C -3.180305 -0.000113 0.634677<br>H -3.796131 -0.883811 0.436864<br>H -2.898581 -0.000204 1.694648<br>H -3.796196 0.883577 0.437033<br>Si 2.515387 0.000031 0.422688<br>P -1.704680 0.000032 -0.509354	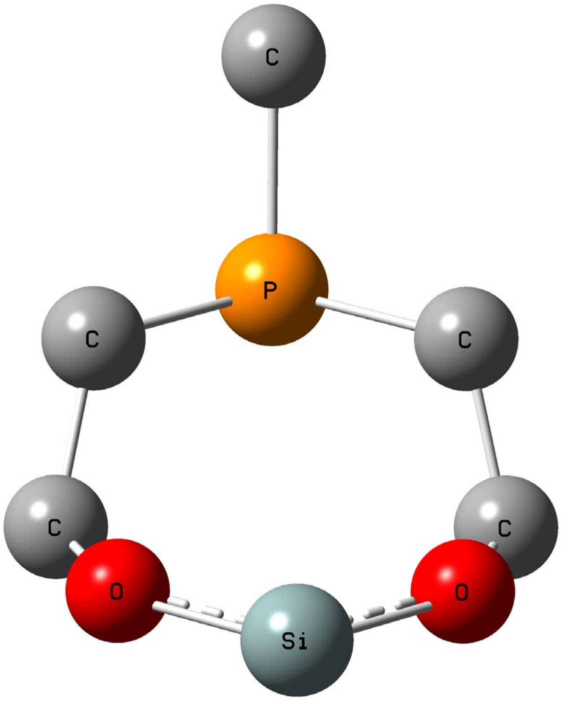

File name: crown_Si_As.log
Optimized Absolute E: -2870.89649174
Optimized XYZ coordinates:
C -0.795620 1.889709 -0.506786
H -0.928919 2.977686 -0.528728
H -0.697412 1.555453 -1.549285
C 0.464389 1.537237 0.297492
H 1.165424 2.376980 0.218922
H 0.218630 1.407646 1.356647
C 0.464389 -1.537240 0.297485
H 1.165422 -2.376984 0.218907
H 0.218634 -1.407656 1.356641
C -0.795624 -1.889701 -0.506791
H -0.928921 -2.977678 -0.528752
H -0.697423 -1.555427 -1.549286
O -1.988582 1.361237 0.055396
O -1.988584 -1.361242 0.055409
C 2.873357 -0.000003 1.018861
H 3.505414 0.885020 0.905906
H 2.410592 -0.000004 2.009781
H 3.505413 -0.885026 0.905903
As 1.502706 0.000000 -0.407460
Si -2.855299 0.000000 0.438681

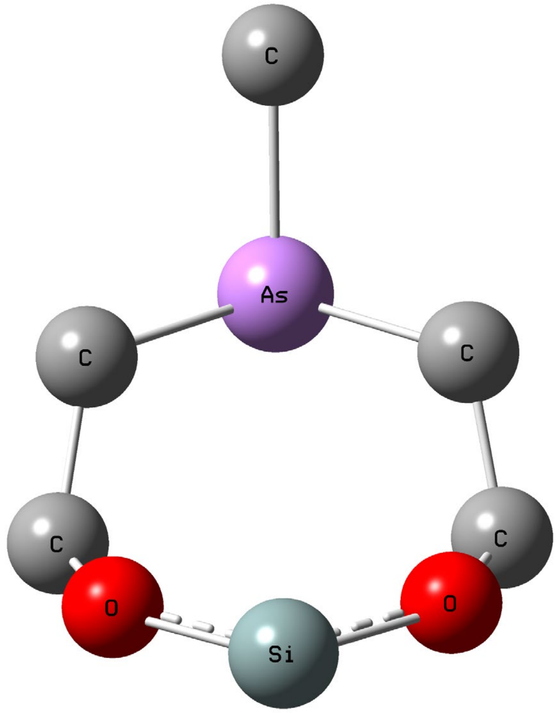

File name: crown_Ge_N.log
Optimized Absolute E: -2477.35306394
Optimized XYZ coordinates:
C 0.442592 2.101340 -0.020653
H 0.368194 3.085281 0.465737
H 0.763157 2.270652 -1.059116
C 1.485942 1.250962 0.702275
H 2.411697 1.841922 0.816513
H 1.115321 1.044458 1.713947
C 1.486949 -1.251746 0.701480
H 2.412817 -1.842950 0.813861
H 1.117891 -1.046211 1.713862
C 0.442758 -2.101439 -0.020855
H 0.368297 -3.085461 0.465370
H 0.763159 -2.270640 -1.059365
O -0.846463 1.527306 -0.021900
O -0.846301 -1.527275 -0.021905
C 2.893291 0.000920 -0.867788
H 2.875704 0.882661 -1.517746

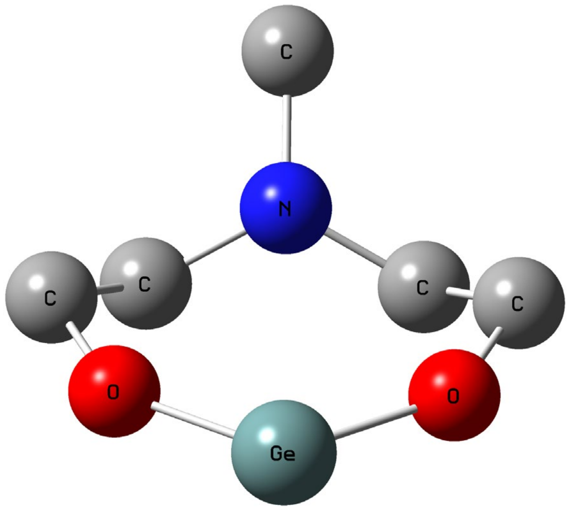

H 3.856115 0.001052 -0.319740<br>H 2.876440 -0.880183 -1.518622<br>Ge -1.814009 -0.000035 -0.099236<br>N 1.736058 0.000009 0.006363	
File name: crown_Ge_P.log<br>Optimized Absolute E: -2763.97180730<br>Optimized XYZ coordinates:<br>C 0.022562 1.815112 -0.758253<br>H -0.028402 2.898104 -0.926770<br>H 0.101528 1.342389 -1.747328<br>C 1.260286 1.488280 0.105261<br>H 1.967811 2.325884 0.042693<br>H 0.948292 1.421235 1.155635<br>C 1.260312 -1.488287 0.105259<br>H 1.967801 -2.325924 0.042705<br>H 0.948246 -1.421323 1.155621<br>C 0.022568 -1.815124 -0.758273<br>H -0.028432 -2.898112 -0.926766<br>H 0.101475 -1.342381 -1.747334<br>O -1.198031 1.457460 -0.133939<br>O -1.198008 -1.457452 -0.133873<br>C 3.539372 0.000043 0.911563<br>H 4.178560 0.883901 0.812909<br>H 3.094705 0.000035 1.914242<br>H 4.178698 -0.883718 0.812957<br>Ge -2.149865 0.000003 0.332679<br>P 2.260208 -0.000025 -0.448343	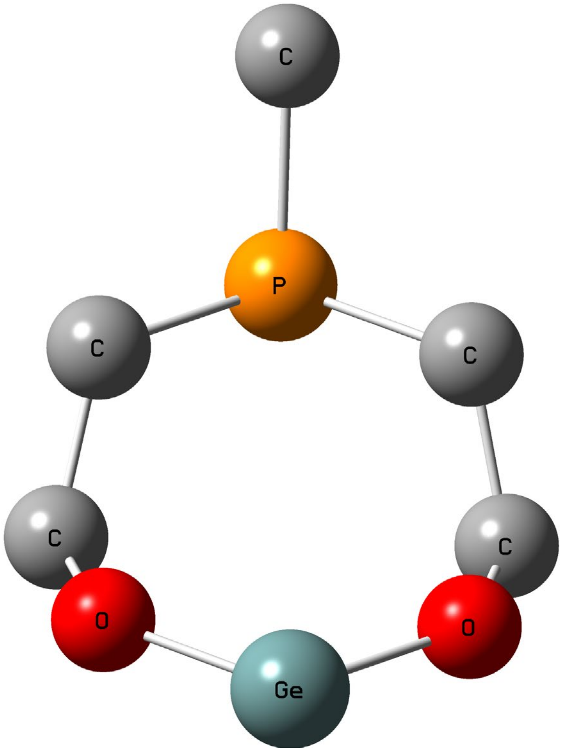

File name: crown_Ge_As.log<br>Optimized Absolute E: -4656.38553706<br>Optimized XYZ coordinates:<br>C 0.320601 -1.820544 -0.709254<br>H 0.379141 -2.897947 -0.910619<br>H 0.196710 -1.323038 -1.682076<br>C -0.881482 -1.529515 0.211623<br>H -1.565945 -2.385399 0.187221<br>H -0.538673 -1.408423 1.244330<br>C -0.881486 1.529521 0.211605<br>H -1.565946 2.385408 0.187182<br>H -0.538686 1.408447 1.244316<br>C 0.320607 1.820526 -0.709267<br>H 0.379144 2.897922 -0.910670<br>H 0.196733 1.322983 -1.682073<br>O 1.561180 -1.461567 -0.125958<br>O 1.561178 1.461575 -0.125937<br>C -3.190391 0.000006 1.228220<br>H -3.831843 -0.884999 1.197095<br>H -2.604733 0.000007 2.151901<br>H -3.831838 0.885014 1.197090<br>Ge 2.517486 0.000001 0.320617<br>As -2.010290 -0.000001 -0.359572	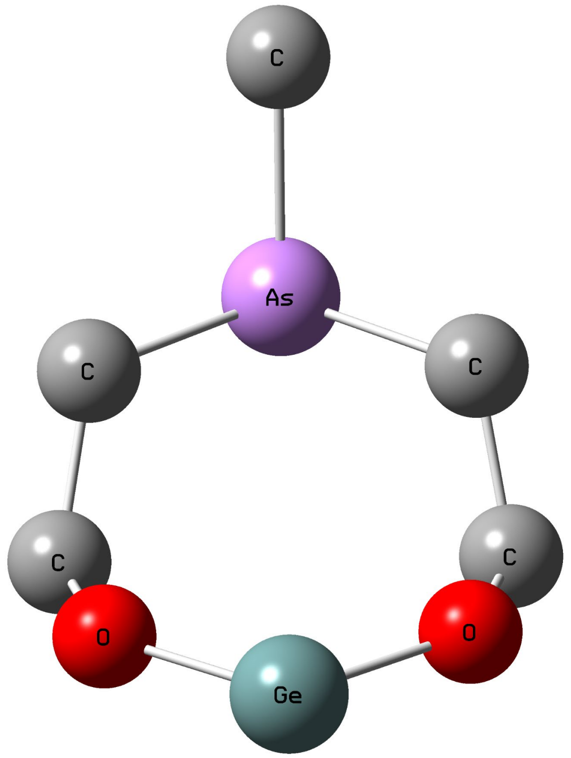
File name: crown_Sn_P_ECP.log<br>Optimized Absolute E: -903.364998399<br>Optimized XYZ coordinates:<br>C -0.488944 -2.238343 -0.118996<br>H -0.407657 -3.213986 0.383679<br>H -0.684410 -2.441584 -1.182708<br>C -1.681004 -1.483938 0.476323<br>H -2.527601 -2.184500 0.498312<br>H -1.470450 -1.208061 1.518615<br>C -1.681055 1.483978 0.476260<br>H -2.527650 2.184546 0.498140<br>H -1.470575 1.208172 1.518585<br>C -0.488949 2.238335 -0.119029<br>H -0.407693 3.214012 0.383584<br>H -0.684340 2.441502 -1.182769<br>O 0.757963 -1.595850 0.009517<br>O 0.757945 1.595843 0.009626<br>C -4.054513 -0.000025 0.064458<br>H -4.568079 -0.884328 -0.327937	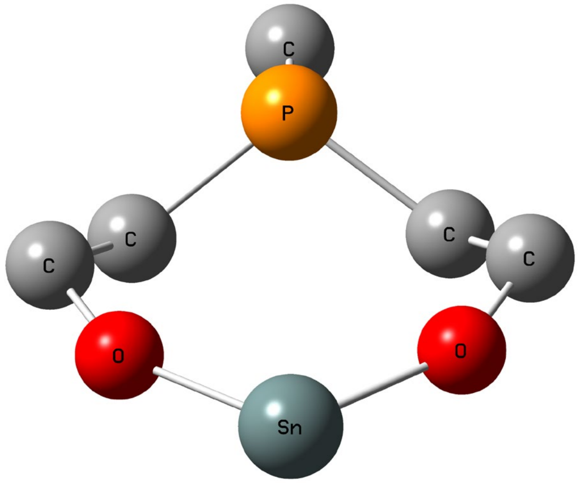

H -4.144534 -0.000001 1.157875<br>H -4.568107 0.884244 -0.327977<br>Sn 1.916926 0.000003 0.000800<br>P -2.276378 -0.000010 -0.520310	
File name: crown_Sn_As_ECP.log<br>Optimized Absolute E: -2795.42434475<br>Optimized XYZ coordinates:<br>C -0.171261 -2.317757 0.148773<br>H -0.018359 -3.218062 0.764139<br>H -0.403152 -2.664935 -0.869832<br>C -1.361302 -1.537070 0.699315<br>H -2.222900 -2.214939 0.754799<br>H -1.154117 -1.183288 1.716417<br>C -1.364093 1.539618 0.696049<br>H -2.225495 2.218168 0.746180<br>H -1.160667 1.189171 1.715064<br>C -0.171708 2.317781 0.147008<br>H -0.020486 3.220076 0.759849<br>H -0.399903 2.661623 -0.873567<br>O 1.046992 -1.610074 0.123082<br>O 1.046239 1.609308 0.128404<br>C -3.836304 -0.001311 0.124138<br>H -4.333312 -0.889227 -0.277744<br>H -3.929960 -0.000036 1.214858<br>H -4.334843 0.884743 -0.279949<br>Sn 2.172086 0.000175 -0.067380<br>As -1.930877 -0.000408 -0.451661	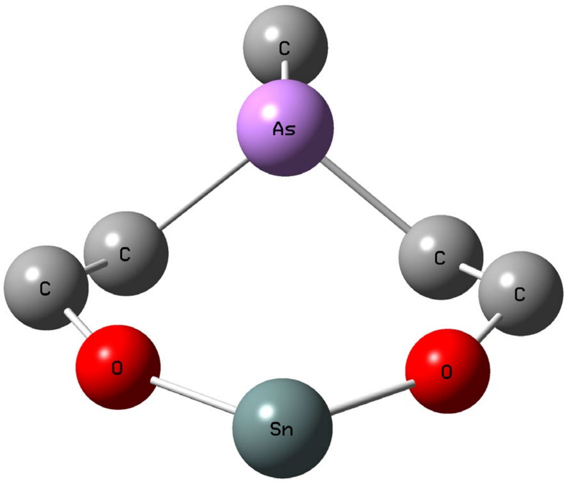
File name: H_boat-boat_Si_N.log<br>Optimized Absolute E: -652.575498360<br>Optimized XYZ coordinates:<br>C -1.635139 0.249203 0.855653<br>H -1.071510 0.410590 1.786668<br>H -2.700586 0.341643 1.104432<br>C -1.264781 1.308230 -0.197529<br>H -2.035371 1.321492 -0.973997<br>H -1.188667 2.314200 0.235653<br>C 1.264759 1.308251 -0.197512<br>H 2.035357 1.321541 -0.973973<br>H 1.188619 2.314213 0.235683<br>C 1.635132 0.249216 0.855655<br>H 1.071492 0.410576 1.786669<br>H 2.700575 0.341675 1.104444	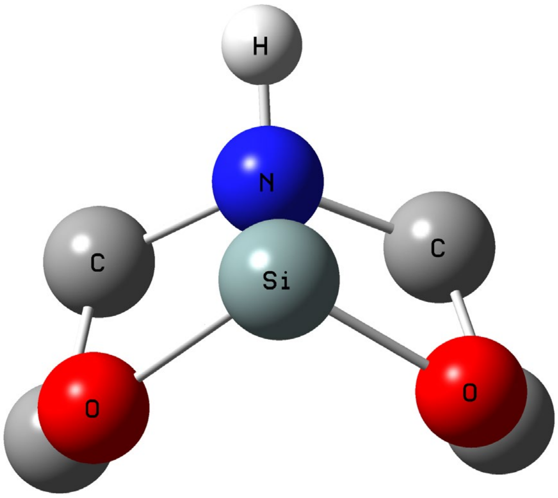

O -1.382205 -1.021355 0.309560<br>O 1.382229 -1.021339 0.309540<br>Si 0.000007 -1.331286 -0.667895<br>N -0.000003 0.901831 -0.854309<br>H 0.000001 1.201418 -1.825286	
File name: OH_boat-boat_Si_N.log<br>Optimized Absolute E: -727.731468035<br>Optimized XYZ coordinates:<br>C -0.435825 -1.615565 -1.004430<br>H -0.743875 -1.048469 -1.894208<br>H -0.469780 -2.679137 -1.274870<br>C 1.002832 -1.259086 -0.587778<br>H 1.368847 -2.017110 0.107761<br>H 1.685250 -1.195524 -1.448549<br>C 1.002747 1.259136 -0.587798<br>H 1.368722 2.017194 0.107725<br>H 1.685160 1.195601 -1.448575<br>C -0.435937 1.615523 -1.004443<br>H -0.743965 1.048390 -1.894205<br>H -0.469957 2.679087 -1.274905<br>O -1.286061 -1.374792 0.088316<br>O -1.286144 1.374723 0.088319<br>Si -1.133393 -0.000030 1.110572<br>N 0.981924 0.000030 0.184771<br>O 2.028108 0.000073 1.153479<br>H 2.863496 0.000096 0.644186	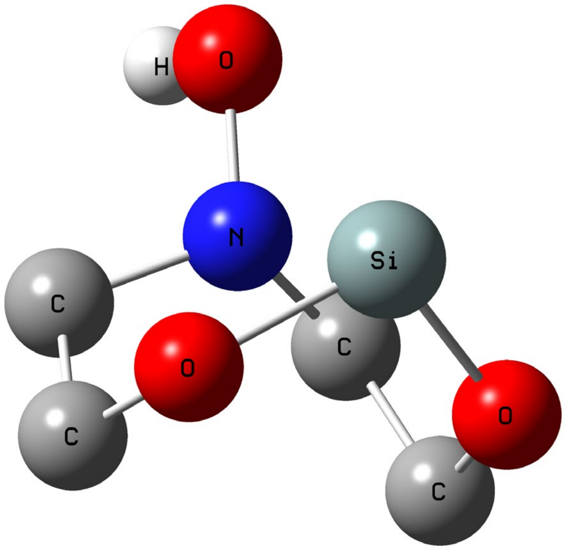

File name: CN_boat-boat_Si_N.log
Optimized Absolute E: -744.796415007
Optimized XYZ coordinates:
C 0.735851 -1.587387 0.956641
H 1.236142 -1.012128 1.743471
H 0.852842 -2.646829 1.214887
C -0.770685 -1.280014 0.913427
H -1.274362 -2.057167 0.334621
H -1.180448 -1.297084 1.934593
C -0.770688 1.280111 0.913307
H -1.274327 2.057214 0.334399
H -1.180493 1.297296 1.934455
C 0.735850 1.587459 0.956554
H 1.236095 1.012266 1.743463
H 0.852850 2.646924 1.214703
O 1.344911 -1.362901 -0.297348
O 1.344963 1.362841 -0.297385
Si 1.546092 -0.000046 -1.260113
N -1.078814 0.000018 0.250177
C -2.155855 -0.000021 -0.554569
N -3.075387 -0.000055 -1.274078

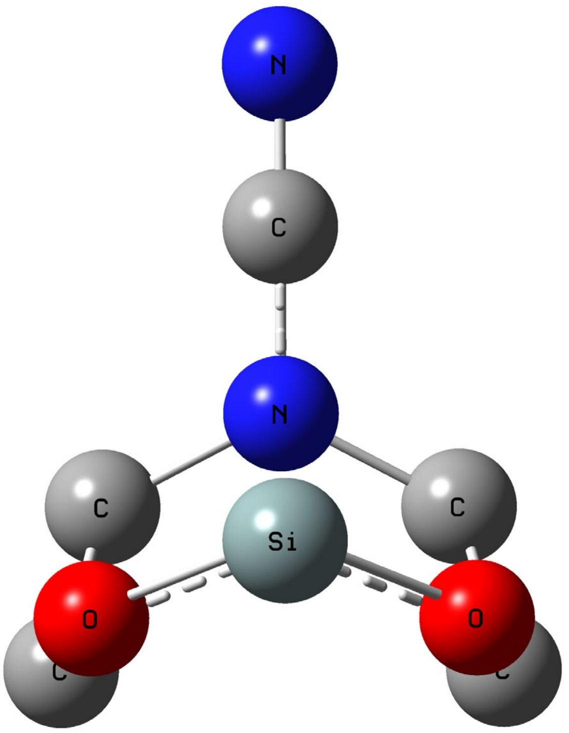

File name: tBut_boat-boat_Si_N.log
Optimized Absolute E: -809.817190714
Optimized XYZ coordinates:
C -1.590295 1.685654 0.668752
H -2.111453 1.221814 1.519135
H -1.816661 2.759972 0.705858
C -0.070400 1.495014 0.779051
H 0.403169 2.212443 0.110914
H 0.281093 1.691527 1.801332
C -0.120884 -0.891464 1.338414
H 0.588648 -1.715720 1.308696
H -0.086200 -0.466647 2.349617
C -1.527847 -1.456713 1.025600
H -2.315496 -0.831651 1.465739
H -1.617849 -2.453460 1.477721
O -2.026729 1.152506 -0.557849
O -1.680714 -1.556551 -0.369505
C 1.742863 -0.022709 -0.134365
Si -1.287741 -0.194905 -1.327485
N 0.291030 0.126950 0.326438
C 2.716118 0.113823 1.053055

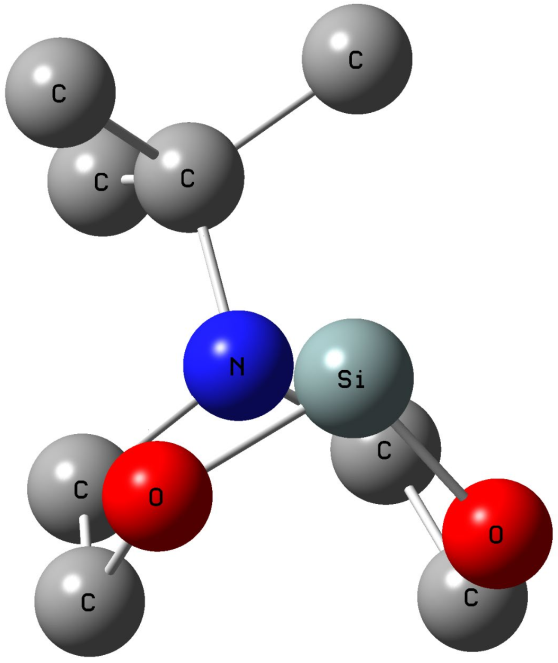

H 3.748715 0.032663 0.696312<br>H 2.614596 1.085909 1.547981<br>H 2.565266 -0.670019 1.802645<br>C 1.925803 -1.394051 -0.816790<br>H 1.260397 -1.492438 -1.679450<br>H 2.958991 -1.477219 -1.169668<br>H 1.745954 -2.242053 -0.151048<br>C 2.075003 1.051308 -1.191058<br>H 2.194987 2.051504 -0.764815<br>H 3.026441 0.793832 -1.667007<br>H 1.307937 1.086746 -1.971359	
File name: H_boat-boat_Si_P.log<br>Optimized Absolute E: -939.165239705<br>Optimized XYZ coordinates:<br>C 0.086781 -1.653017 0.945662<br>H 0.148785 -1.028079 1.843741<br>H 0.206487 -2.692914 1.271201<br>C -1.289230 -1.482182 0.276973<br>H -1.498782 -2.349409 -0.361261<br>H -2.062686 -1.450370 1.053707<br>C -1.289234 1.482178 0.276972<br>H -1.498789 2.349405 -0.361263<br>H -2.062691 1.450365 1.053705<br>C 0.086776 1.653018 0.945662<br>H 0.148781 1.028081 1.843741<br>H 0.206478 2.692915 1.271200<br>O 1.168901 -1.361229 0.069851<br>O 1.168897 1.361232 0.069852<br>Si 1.826046 0.000003 -0.657840<br>P -1.375696 -0.000002 -0.876754<br>H -2.789739 -0.000004 -1.042940	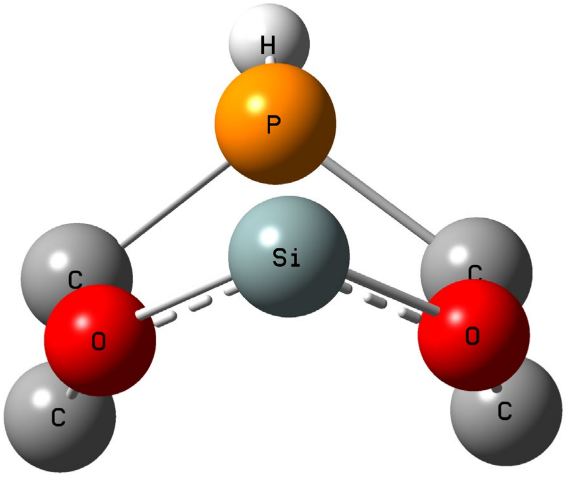

File name: OH_boat-boat_Si_P.log<br>Optimized Absolute E: -1014.39759281<br>Optimized XYZ coordinates:<br>C -0.534661 -1.643003 -0.969053<br>H -0.751603 -1.022919 -1.845492<br>H -0.704584 -2.684008 -1.266034<br>C 0.927720 -1.461702 -0.534605<br>H 1.256425 -2.333561 0.044082<br>H 1.563817 -1.409147 -1.430878<br>C 0.927722 1.461703 -0.534602<br>H 1.256427 2.333560 0.044087<br>H 1.563820 1.409150 -1.430875<br>C -0.534658 1.643007 -0.969051<br>H -0.751600 1.022927 -1.845493<br>H -0.704581 2.684013 -1.266027<br>O -1.464345 -1.354039 0.074865<br>O -1.464344 1.354038 0.074864<br>Si -2.084981 0.000000 0.838841<br>P 1.237697 -0.000001 0.604393<br>O 2.911103 -0.000002 0.657337<br>H 3.320097 -0.000002 -0.225708	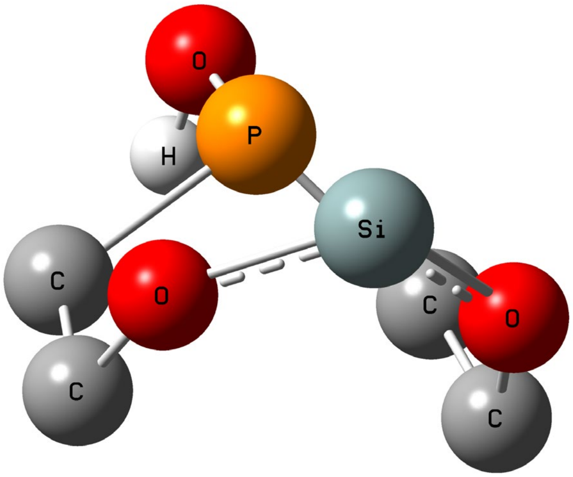
File name: CN_boat-boat_Si_P.log<br>Optimized Absolute E: -1031.40970154<br>Optimized XYZ coordinates:<br>C -0.802505 -1.649389 -0.954269<br>H -1.046245 -1.025933 -1.820925<br>H -0.987381 -2.689350 -1.245495<br>C 0.683038 -1.491420 -0.587218<br>H 1.015388 -2.352105 0.004356<br>H 1.285179 -1.465106 -1.502111<br>C 0.683038 1.491419 -0.587218<br>H 1.015388 2.352105 0.004356<br>H 1.285178 1.465106 -1.502112<br>C -0.802506 1.649389 -0.954269<br>H -1.046245 1.025933 -1.820925<br>H -0.987381 2.689349 -1.245495<br>O -1.678983 -1.353930 0.127653<br>O -1.678984 1.353930 0.127653<br>Si -2.286396 -0.000000 0.906555<br>P 1.011954 0.000000 0.512912<br>C 2.803108 0.000000 0.394708<br>N 3.967865 0.000000 0.404294	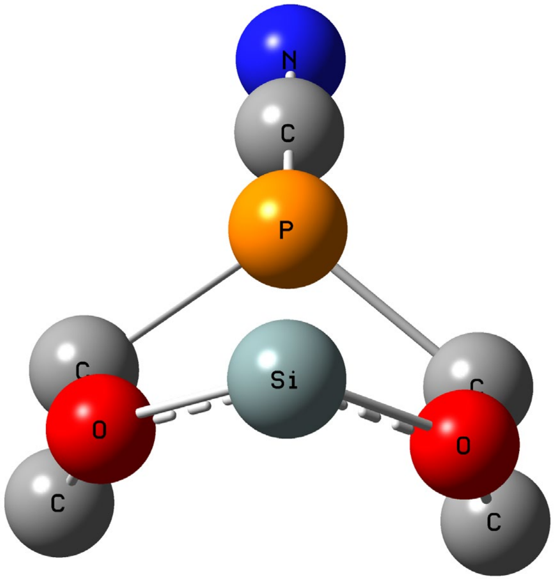

File name: tBut_boat-boat_Si_P.log
Optimized Absolute E: -1096.42200344
Optimized XYZ coordinates:
C 1.659445 -1.659280 0.948664
H 2.106399 -1.034727 1.732940
H 1.895942 -2.701101 1.198073
C 0.123994 -1.474521 0.941519
H -0.342898 -2.344710 0.466267
H -0.252857 -1.410307 1.969046
C 0.123945 1.474731 0.941293
H -0.342874 2.344851 0.465841
H -0.252993 1.410719 1.968800
C 1.659401 1.659427 0.948538
H 2.106253 1.035019 1.732989
H 1.895922 2.701291 1.197748
O 2.238091 -1.372731 -0.310632
O 2.238160 1.372589 -0.310640
C -2.236536 -0.000027 -0.196738
Si 2.135021 -0.000070 -1.318620
P -0.339899 0.000020 -0.102257
C -2.661242 1.254102 -0.988059
H -3.747689 1.239005 -1.143442
H -2.421490 2.180706 -0.455591
H -2.182161 1.292661 -1.972549
C -2.910917 0.000008 1.186783
H -4.003033 -0.000014 1.067892
H -2.645353 -0.887107 1.771885
H -2.645382 0.887169 1.771828
C -2.661177 -1.254237 -0.987965
H -3.747631 -1.239230 -1.143311
H -2.182126 -1.292824 -1.972468
H -2.421339 -2.180791 -0.455448

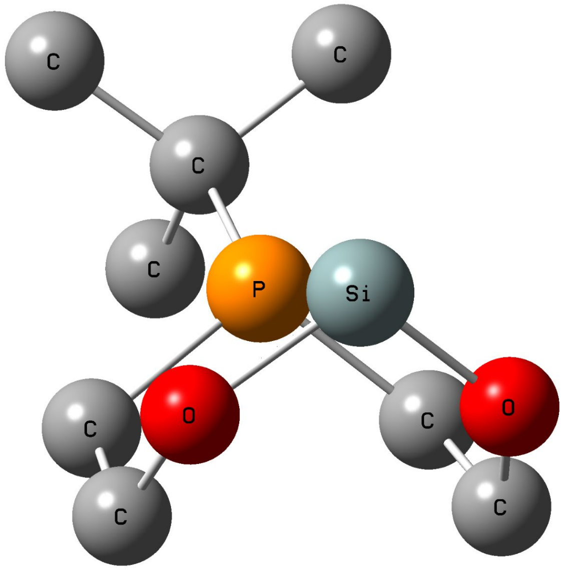

File name: H_boat-boat_Si_As.log<br>Optimized Absolute E: -2831.57933038<br>Optimized XYZ coordinates:<br>C 0.565513 -1.665722 1.060575<br>H 0.792567 -1.028052 1.922038<br>H 0.779227 -2.698341 1.360929<br>C -0.911725 -1.529550 0.671943<br>H -1.236587 -2.408053 0.104824<br>H -1.537632 -1.446608 1.565325<br>C -0.911727 1.529550 0.671942<br>H -1.236590 2.408052 0.104821<br>H -1.537635 1.446608 1.565324<br>C 0.565511 1.665724 1.060574<br>H 0.792565 1.028055 1.922039<br>H 0.779224 2.698343 1.360927<br>O 1.447158 -1.360854 -0.019268<br>O 1.447157 1.360855 -0.019267<br>As -1.257347 -0.000001 -0.555802<br>Si 1.977002 0.000001 -0.841552<br>H -2.780686 -0.000002 -0.264961	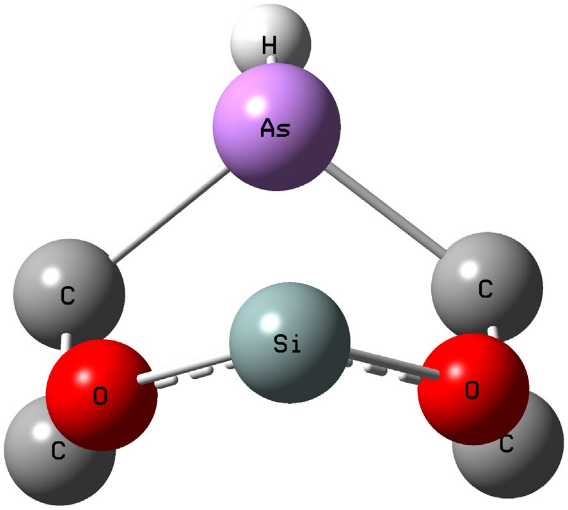
File name: OH_boat-boat_Si_As.log<br>Optimized Absolute E: -2906.81109017<br>Optimized XYZ coordinates:<br>C -0.850374 -1.665956 -1.054220<br>H -1.129742 -1.033009 -1.903456<br>H -1.082012 -2.699761 -1.334888<br>C 0.641405 -1.518750 -0.741527<br>H 1.010220 -2.396770 -0.201290<br>H 1.222955 -1.419246 -1.666139<br>C 0.641405 1.518750 -0.741527<br>H 1.010220 2.396770 -0.201289<br>H 1.222956 1.419247 -1.666139<br>C -0.850374 1.665957 -1.054220<br>H -1.129742 1.033010 -1.903456<br>H -1.082012 2.699762 -1.334888<br>O -1.672585 -1.355415 0.075805<br>O -1.672586 1.355415 0.075805<br>As 1.039169 -0.000000 0.484178<br>Si -2.207994 -0.000000 0.899569<br>O 2.845229 -0.000000 0.324166<br>H 3.083655 0.000000 -0.617537	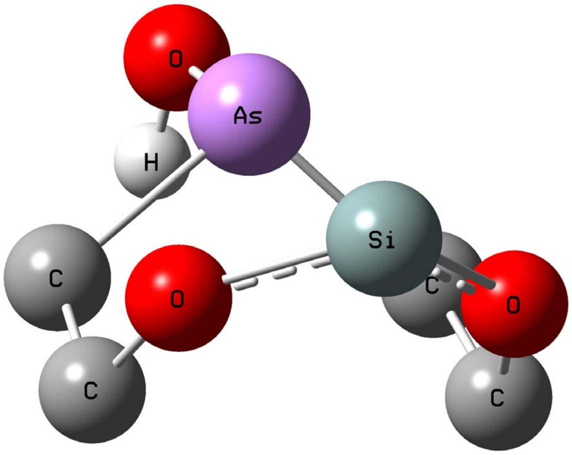

File name: CN_boat-boat_Si_As.log<br>Optimized Absolute E: -2923.83253430<br>Optimized XYZ coordinates:<br>C -1.020587 -1.662073 -1.052309<br>H -1.289322 -1.025261 -1.901457<br>H -1.246172 -2.695052 -1.340100<br>C 0.474431 -1.531724 -0.734407<br>H 0.820501 -2.405981 -0.175323<br>H 1.066574 -1.441979 -1.648884<br>C 0.473980 1.532304 -0.734540<br>H 0.819867 2.406775 -0.175678<br>H 1.065945 1.442595 -1.649135<br>C -1.021192 1.662037 -1.052111<br>H -1.289776 1.025328 -1.901383<br>H -1.247320 2.694986 -1.339574<br>O -1.844372 -1.355660 0.073230<br>O -1.844677 1.354931 0.073485<br>As 0.831938 0.000443 0.486246<br>Si -2.393480 -0.000529 0.893414<br>C 2.718946 0.000127 0.222525<br>N 3.873343 -0.000974 0.072708	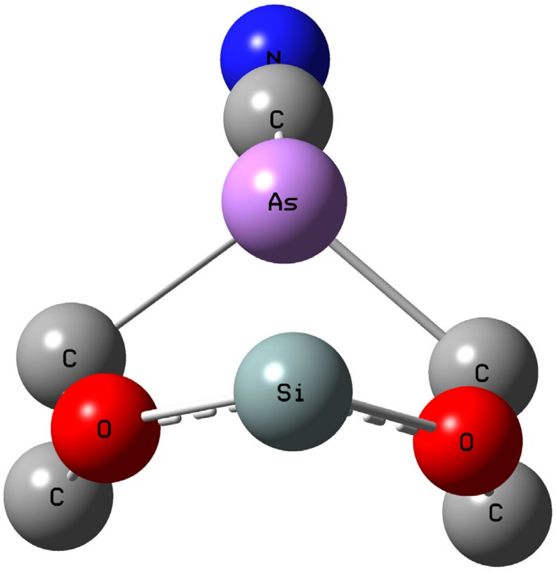
File name: tBut_boat-boat_Si_As.log<br>Optimized Absolute E: -2988.84891563<br>Optimized XYZ coordinates:<br>C 1.748330 -1.665757 0.985877<br>H 2.180993 -1.028169 1.766963<br>H 2.015024 -2.700281 1.235287<br>C 0.214219 -1.516553 0.970383<br>H -0.235774 -2.401791 0.510303<br>H -0.182085 -1.401518 1.983400<br>C 0.214219 1.516562 0.970376<br>H -0.235751 2.401802 0.510279<br>H -0.182100 1.401546 1.983390<br>C 1.748331 1.665741 0.985888<br>H 2.180978 1.028127 1.766963<br>H 2.015042 2.700255 1.235323<br>O 2.329234 -1.370692 -0.276136<br>O 2.329236 1.370694 -0.276129<br>C -2.309349 -0.000001 -0.149123<br>As -0.288930 0.000002 -0.195563<br>Si 2.268765 0.000003 -1.285403<br>C -2.772944 1.256904 -0.902736	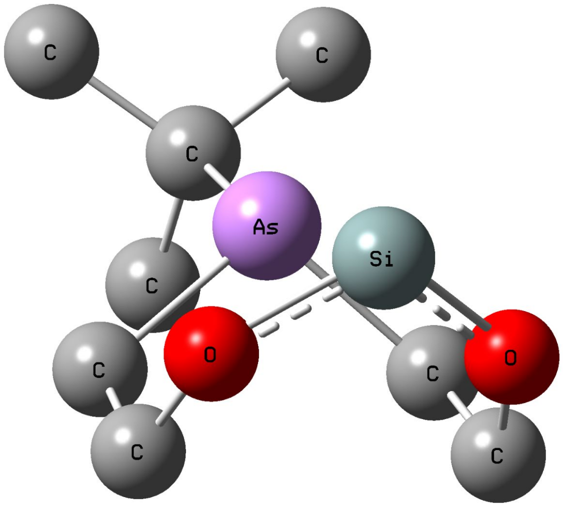

H -3.869100 1.267821 -0.976699<br>H -2.472863 2.178592 -0.391509<br>H -2.370890 1.289519 -1.921839<br>C -2.843098 -0.000000 1.288051<br>H -3.942767 -0.000002 1.286456<br>H -2.522130 -0.885882 1.846585<br>H -2.522134 0.885884 1.846583<br>C -2.772940 -1.256909 -0.902734<br>H -3.869097 -1.267832 -0.976692<br>H -2.370890 -1.289521 -1.921839<br>H -2.472852 -2.178595 -0.391508	
File name: H_boat-boat_Ge_N.log<br>Optimized Absolute E: -2438.07407574<br>Optimized XYZ coordinates:<br>C -0.764224 -1.642795 0.879935<br>H -1.119879 -1.047731 1.735694<br>H -0.980843 -2.696265 1.113457<br>C -1.537452 -1.259418 -0.394043<br>H -1.361314 -2.028142 -1.152733<br>H -2.618404 -1.180348 -0.216371<br>C -1.522249 1.277738 -0.393923<br>H -1.337056 2.044329 -1.152613<br>H -2.604043 1.211554 -0.216121<br>C -0.744332 1.651834 0.879951<br>H -1.106913 1.061027 1.735752<br>H -0.948353 2.707805 1.113527<br>O 0.606048 -1.473961 0.657059<br>O 0.623793 1.466700 0.656852<br>N -0.991029 0.005952 -0.944842<br>Ge 1.177133 -0.007079 -0.335384<br>H -1.083453 0.006556 -1.957225	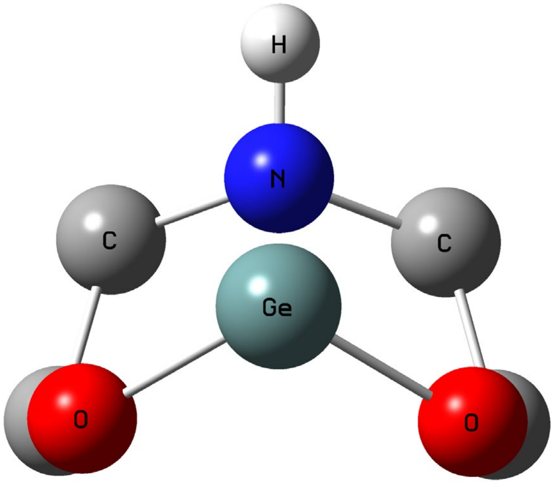

File name: OH_boat-boat_Ge_N.log
Optimized Absolute E: -2513.23145662
Optimized XYZ coordinates:
C 0.175616 -1.631155 -1.260249
H 0.165139 -1.036706 -2.186288
H 0.283409 -2.684247 -1.561499
C 1.394274 -1.262972 -0.392392
H 1.504702 -2.016933 0.389917
H 2.325582 -1.197742 -0.974676
C 1.394120 1.263061 -0.392552
H 1.504563 2.017112 0.389666
H 2.325381 1.197834 -0.974912
C 0.175358 1.631075 -1.260339
H 0.164822 1.036521 -2.186308
H 0.283060 2.684140 -1.561716
O -0.991208 -1.462555 -0.509857
O -0.991382 1.462491 -0.509813
N 1.125591 0.000072 0.329742
Ge -1.142993 -0.000055 0.633428
O 1.801030 0.000189 1.587754
H 2.756246 0.000229 1.376453

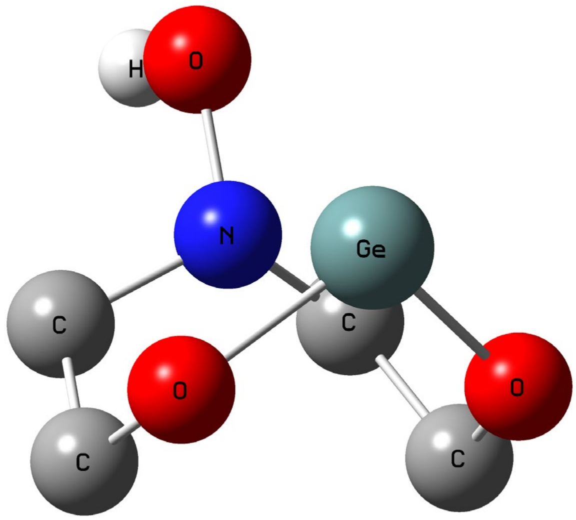

File name: CN_boat-boat_Ge_N.log
Optimized Absolute E: -2530.29222986
Optimized XYZ coordinates:
C -0.334251 -1.618412 -1.330134
H -0.681390 -1.012605 -2.177584
H -0.355654 -2.666861 -1.660482
C 1.123024 -1.278224 -0.973230
H 1.515733 -2.056207 -0.315096
H 1.754341 -1.227140 -1.870346
C 1.123090 1.279833 -0.971160
H 1.515470 2.056787 -0.311614
H 1.754699 1.230325 -1.868161
C -0.334117 1.620366 -1.327963
H -0.680881 1.015873 -2.176509
H -0.355580 2.669341 -1.656634
O -1.158994 -1.468464 -0.208095
O -1.159197 1.468493 -0.206435
N 1.215632 0.000191 -0.212210
Ge -1.178433 -0.000617 0.898902
C 2.170410 -0.000605 0.750170
N 2.970043 -0.001301 1.598166

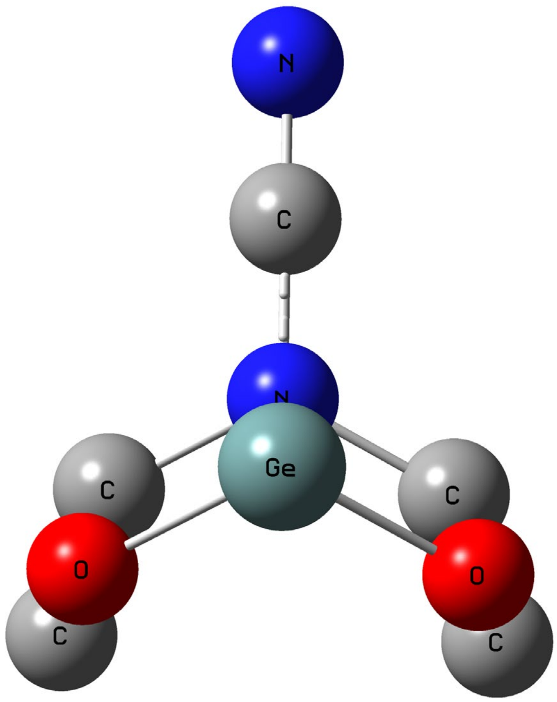

File name: tBut_boat-boat_Ge_N.log<br>Optimized Absolute E: -2595.31973231<br>Optimized XYZ coordinates:<br>C 1.220812 -0.802255 1.793427<br>H 1.970676 -0.017011 1.956623<br>H 1.266263 -1.480823 2.658513<br>C -0.203779 -0.196549 1.729099<br>H -0.915058 -0.984227 1.969926<br>H -0.331984 0.600004 2.472477<br>C -0.246054 1.783565 0.267095<br>H -0.568658 2.120707 -0.717603<br>H -0.797687 2.347396 1.031960<br>C 1.266059 2.031246 0.363913<br>H 1.600397 1.965368 1.412673<br>H 1.461698 3.064957 0.038330<br>O 1.476964 -1.513052 0.614073<br>O 1.949951 1.128453 -0.457255<br>C -1.906429 -0.093784 -0.143745<br>N -0.521687 0.323788 0.359839<br>Ge 1.163775 -0.502713 -0.907584<br>C -3.009827 0.375776 0.825552<br>H -2.987959 1.462034 0.964655<br>H -3.992121 0.116375 0.416239<br>H -2.929459 -0.097610 1.809017<br>C -2.176136 0.514079 -1.535836<br>H -1.336716 0.349103 -2.220077<br>H -3.055086 0.023221 -1.965766<br>H -2.394878 1.585140 -1.497698<br>C -1.953900 -1.628363 -0.300251<br>H -2.970910 -1.924063 -0.578999<br>H -1.274968 -1.967469 -1.089039<br>H -1.692328 -2.168321 0.612517	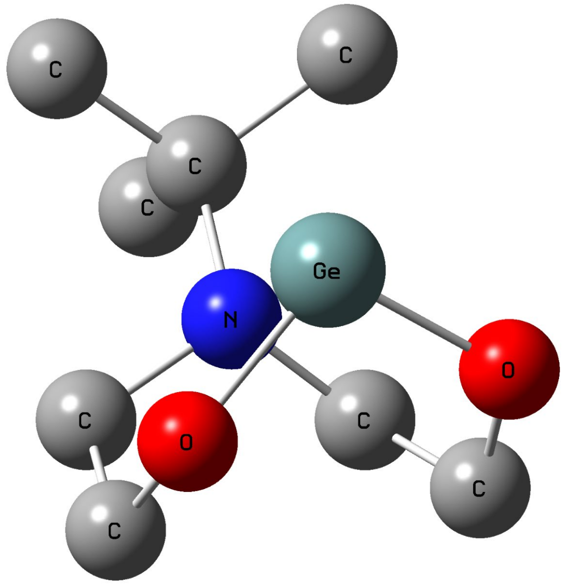

File name: H_boat-boat_Ge_P.log
Optimized Absolute E: -2724.66184351
Optimized XYZ coordinates:
C -0.500583 -1.704377 1.090882
H -0.696171 -1.059501 1.959997
H -0.542470 -2.742536 1.449677
C -1.621403 -1.498927 0.042669
H -1.631924 -2.341374 -0.659823
H -2.604899 -1.441941 0.522851
C -1.621412 1.498918 0.042667
H -1.631936 2.341365 -0.659826
H -2.604909 1.441928 0.522847
C -0.500595 1.704374 1.090882
H -0.696182 1.059499 1.959997
H -0.542486 2.742535 1.449676
O 0.778763 -1.472253 0.557802
O 0.778754 1.472255 0.557804
Ge 1.398162 0.000004 -0.386969
P -1.233819 -0.000004 -0.983419
H -2.279099 -0.000007 -1.938553

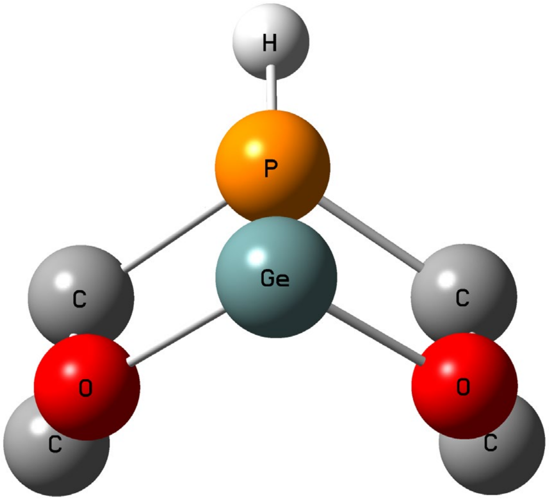

File name: OH_boat-boat_Ge_P.log
Optimized Absolute E: -2799.89369702
Optimized XYZ coordinates:
C -0.090700 -1.695371 -1.305779
H -0.183422 -1.053571 -2.193720
H -0.159479 -2.734012 -1.657835
C 1.301155 -1.481291 -0.662351
H 1.540543 -2.323779 -0.001628
H 2.083413 -1.412354 -1.429209
C 1.301161 1.481292 -0.662344
H 1.540548 2.323777 -0.001617
H 2.083422 1.412358 -1.429200
C -0.090692 1.695376 -1.305775
H -0.183411 1.053581 -2.193720
H -0.159469 2.734019 -1.657826
O -1.141787 -1.467182 -0.396903
O -1.141783 1.467182 -0.396904
Ge -1.434686 0.000001 0.705110
P 1.232285 -0.000002 0.446752
O 2.645904 -0.000007 1.314515
H 3.439333 -0.000007 0.751789

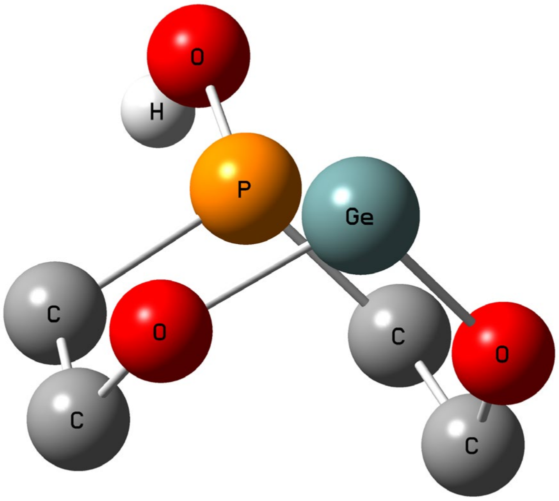

File name: CN_boat-boat_Ge_P.log
Optimized Absolute E: -2816.90348731
Optimized XYZ coordinates:
C 0.436768 -1.693491 1.297600
H 0.655051 -1.045639 2.156843
H 0.561687 -2.729852 1.638492
C -1.031801 -1.496599 0.857948
H -1.356054 -2.342222 0.240660
H -1.698516 -1.428948 1.724211
C -1.031804 1.496603 0.857942
H -1.356055 2.342223 0.240650
H -1.698519 1.428956 1.724204
C 0.436765 1.693496 1.297596
H 0.655046 1.045648 2.156843
H 0.561685 2.729859 1.638483
O 1.343150 -1.467842 0.242792
O 1.343149 1.467841 0.242792
Ge 1.627399 -0.000001 -0.831698
P -1.156877 -0.000000 -0.247721
C -2.893717 -0.000002 -0.653893
N -4.005084 -0.000004 -1.002581

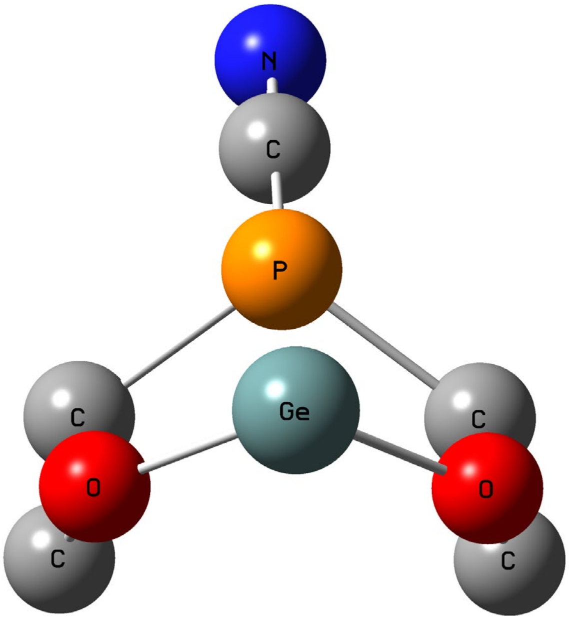

File name: tBut_boat-boat_Ge_P.log
Optimized Absolute E: -2881.92282075
Optimized XYZ coordinates:
C 1.300860 -1.692632 1.308660
H 1.705176 -1.041529 2.097837
H 1.491497 -2.728315 1.626781
C -0.235739 -1.488165 1.221915
H -0.682976 -2.344510 0.704534
H -0.680625 -1.422484 2.221845
C -0.235742 1.488238 1.221838
H -0.682958 2.344559 0.704400
H -0.680645 1.422619 2.221763
C 1.300859 1.692691 1.308599
H 1.705151 1.041628 2.097822
H 1.491503 2.728389 1.626664
O 1.943896 -1.476604 0.081403
O 1.943916 1.476584 0.081367
C -2.416736 -0.000007 -0.289028
Ge 1.725269 -0.000022 -1.042709
P -0.586322 0.000009 0.171832
C -3.329805 0.000049 0.950646

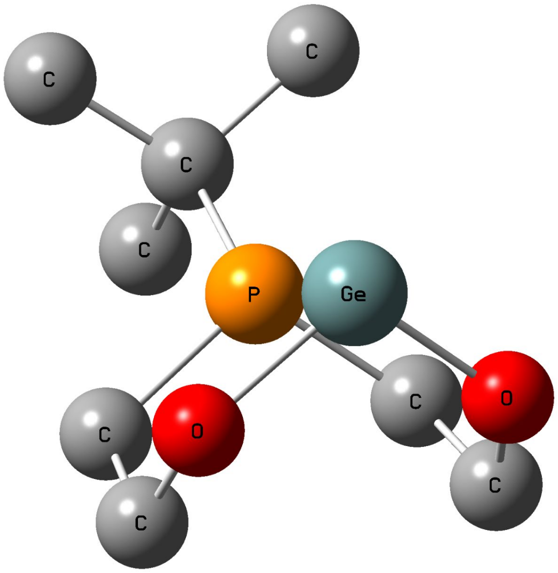

H -4.382256 0.000036 0.636767<br>H -3.171246 -0.887131 1.573406<br>H -3.171244 0.887285 1.573327<br>C -2.681881 1.255906 -1.144934<br>H -2.016969 1.299920 -2.014630<br>H -3.716022 1.236339 -1.511195<br>H -2.555633 2.180582 -0.572073<br>C -2.681883 -1.255997 -1.144820<br>H -3.716028 -1.236469 -1.511072<br>H -2.016980 -1.300085 -2.014518<br>H -2.555623 -2.180621 -0.571878	
File name: H_boat-boat_Ge_As.log<br>Optimized Absolute E: -4617.07447937<br>Optimized XYZ coordinates:<br>C 0.044088 1.704473 1.308450<br>H 0.042394 1.042776 2.185152<br>H -0.028953 2.733808 1.686298<br>C 1.367137 1.541119 0.538423<br>H 1.539886 2.403711 -0.113419<br>H 2.222521 1.431745 1.211528<br>C 1.367150 -1.541114 0.538416<br>H 1.539901 -2.403703 -0.113430<br>H 2.222536 -1.431738 1.211518<br>C 0.044105 -1.704477 1.308448<br>H 0.042413 -1.042784 2.185154<br>H -0.028932 -2.733814 1.686290<br>O -1.084953 1.468456 0.492743<br>O -1.084941 -1.468458 0.492748<br>Ge -1.587832 -0.000005 -0.500267<br>As 1.241964 0.000005 -0.690452<br>H 2.698301 0.000012 -1.191991	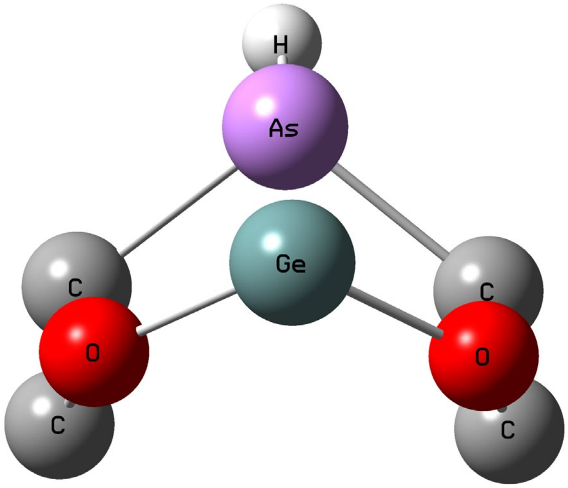

File name: OH_boat-boat_Ge_As.log
Optimized Absolute E: -4692.30581928
Optimized XYZ coordinates:
C 0.362601 -1.697467 1.354779
H 0.523468 -1.037855 2.217247
H 0.503439 -2.726676 1.711053
C -1.067806 -1.525528 0.820936
H -1.363058 -2.390778 0.218390
H -1.791758 -1.402445 1.634495
C -1.067822 1.525540 0.820913
H -1.363067 2.390781 0.218352
H -1.791782 1.402468 1.634466
C 0.362580 1.697482 1.354769
H 0.523435 1.037883 2.217249
H 0.503417 2.726697 1.711029
O 1.338545 -1.462290 0.352080
O 1.338534 1.462288 0.352085
Ge 1.746767 0.000002 -0.678714
As -1.151409 -0.000004 -0.436323
O -2.895572 -0.000017 -0.888433
H -3.433532 -0.000014 -0.079020

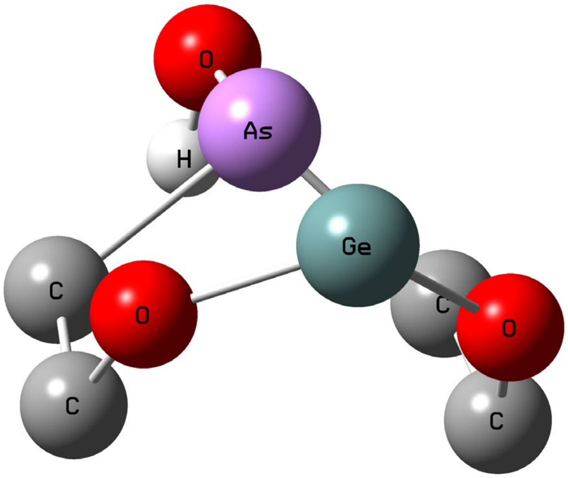

File name: CN_boat-boat_Ge_As.log
Optimized Absolute E: -4709.32734297
Optimized XYZ coordinates:
C 0.584793 -1.689027 1.330139
H 0.789720 -1.022561 2.176373
H 0.743689 -2.715464 1.686034
C -0.876429 -1.536925 0.876544
H -1.181860 -2.404384 0.284196
H -1.558326 -1.425280 1.723706
C -0.876436 1.536939 0.876526
H -1.181860 2.404391 0.284165
H -1.558340 1.425305 1.723683
C 0.584782 1.689040 1.330130
H 0.789699 1.022584 2.176375
H 0.743681 2.715481 1.686013
O 1.499597 -1.462234 0.272037
O 1.499595 1.462229 0.272040
Ge 1.959970 -0.000001 -0.725072
As -1.046710 -0.000001 -0.366414
C -2.933703 -0.000007 -0.506229
N -4.093655 -0.000010 -0.605980

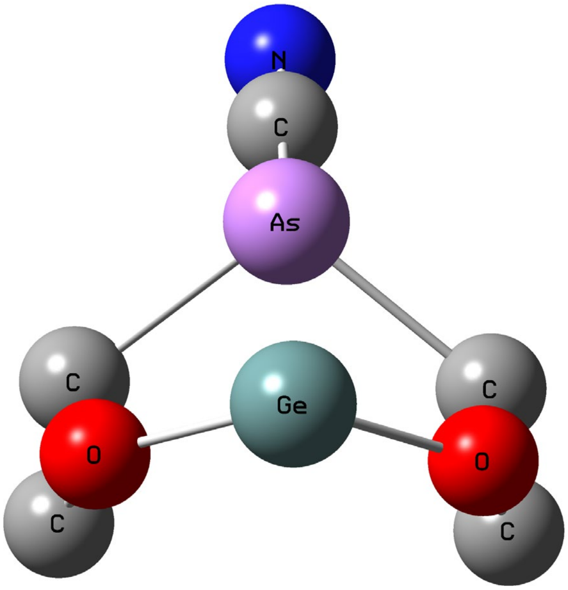

File name: tBut_boat-boat_Ge_As.log<br>Optimized Absolute E: -4774.34898728<br>Optimized XYZ coordinates:<br>C 1.371718 -1.691598 1.326988<br>H 1.735947 -1.025648 2.121914<br>H 1.585804 -2.719412 1.654936<br>C -0.158112 -1.528441 1.184502<br>H -0.574010 -2.403390 0.675422<br>H -0.645788 -1.411413 2.156975<br>C -0.158104 1.528601 1.184346<br>H -0.573931 2.403516 0.675151<br>H -0.645827 1.411710 2.156814<br>C 1.371727 1.691698 1.326872<br>H 1.735897 1.025800 2.121868<br>H 1.585851 2.719530 1.654738<br>O 2.061326 -1.470622 0.119055<br>O 2.061367 1.470587 0.118979<br>C -2.521585 -0.000016 -0.295433<br>Ge 1.968297 -0.000047 -1.016162<br>As -0.531647 0.000022 -0.003243<br>C -3.270440 0.000394 1.043175<br>H -4.356336 0.000372 0.871091<br>H -3.037916 -0.885587 1.643792<br>H -3.037870 0.886715 1.643274<br>C -2.856458 1.258219 -1.111907<br>H -2.297001 1.290314 -2.053748<br>H -3.926582 1.270268 -1.359515<br>H -2.640365 2.178773 -0.558349<br>C -2.856494 -1.258729 -1.111153<br>H -3.926640 -1.270954 -1.358662<br>H -2.297115 -1.291354 -2.053022<br>H -2.640322 -2.178954 -0.557077	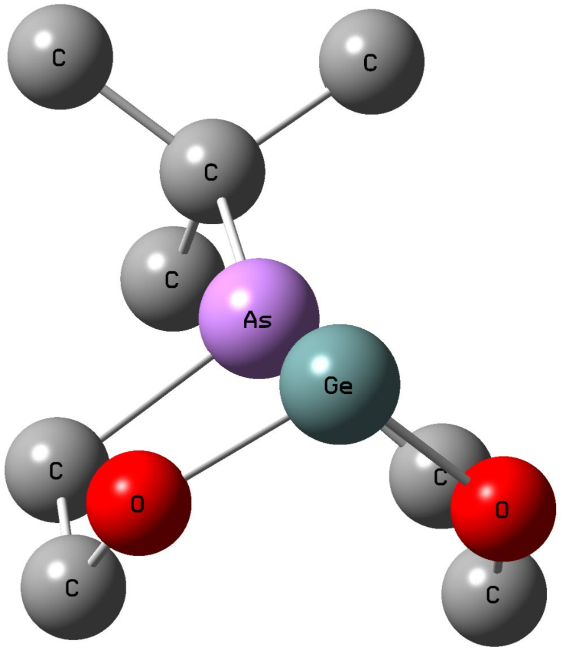

File name: H_boat-boat_Sn_N_ECP.log<br>Optimized Absolute E: -577.469829082<br>Optimized XYZ coordinates:<br>C 1.126698 1.675778 0.850343<br>H 1.527342 1.063201 1.674141<br>H 1.423319 2.715419 1.061125<br>C 1.791470 1.265843 -0.478303<br>H 1.579372 2.037227 -1.226321<br>H 2.882459 1.183852 -0.366269<br>C 1.791496 -1.265807 -0.478316<br>H 1.579397 -2.037191 -1.226333<br>H 2.882486 -1.183801 -0.366298<br>C 1.126752 -1.675755 0.850340<br>H 1.527409 -1.063180 1.674134<br>H 1.423384 -2.715396 1.061109<br>O -0.265795 1.582554 0.763365<br>O -0.265743 -1.582540 0.763392<br>Sn -1.108111 -0.000014 -0.198775<br>N 1.218389 0.000014 -1.001321<br>H 1.285490 0.000020 -2.015738	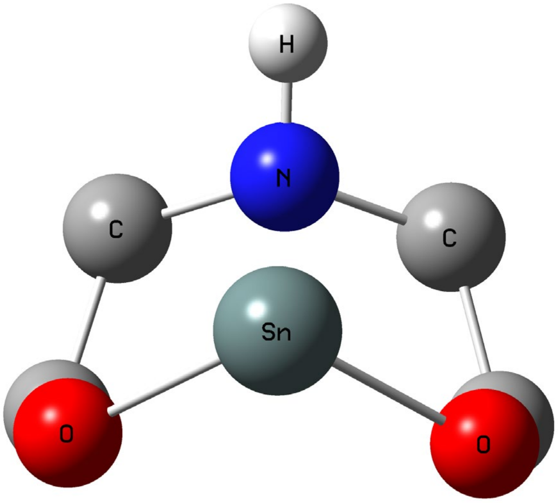
File name: OH_boat-boat_Sn_N_ECP.log<br>Optimized Absolute E: -652.627533720<br>Optimized XYZ coordinates:<br>C -0.718267 1.658647 -1.277428<br>H -0.878385 1.044762 -2.177372<br>H -0.952979 2.696267 -1.562878<br>C -1.720370 1.259837 -0.173092<br>H -1.692361 2.019038 0.612236<br>H -2.748154 1.182618 -0.562338<br>C -1.720315 -1.259871 -0.173208<br>H -1.692357 -2.019125 0.612071<br>H -2.748071 -1.182631 -0.562524<br>C -0.718128 -1.658611 -1.277496<br>H -0.878159 -1.044652 -2.177405<br>H -0.952831 -2.696204 -1.563046<br>O 0.595239 1.572008 -0.809482<br>O 0.595339 -1.572028 -0.809430<br>N -1.312497 -0.000037 0.486810<br>O -1.758288 -0.000105 1.845969<br>H -2.736373 -0.000121 1.809294<br>Sn 1.165426 0.000026 0.355104	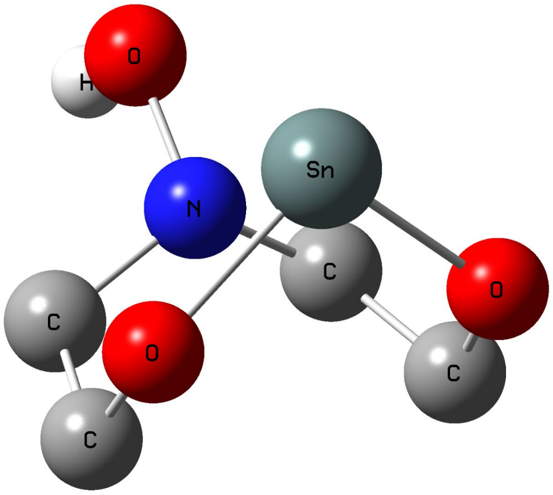

File name: CN_boat-boat_Sn_N_ECP.log
Optimized Absolute E: -669.686812999
Optimized XYZ coordinates:
C -0.285786 1.323042 1.800181
H -0.144067 2.277613 1.273373
H -0.402878 1.569609 2.866438
C -1.598999 0.653731 1.355923
H -1.834511 -0.156305 2.050171
H -2.431012 1.371178 1.364235
C -1.598665 0.920233 -1.191638
H -1.832039 0.271050 -2.038817
H -2.431849 1.622706 -1.052375
C -0.286091 1.668773 -1.485834
H -0.146224 2.493174 -0.771970
H -0.402848 2.131404 -2.477675
O 0.808406 0.467981 1.631780
O 0.809132 0.798669 -1.497798
Sn 1.233054 -0.547021 -0.057500
N -1.489440 0.031235 0.003086
C -2.165068 -1.136785 -0.119252
N -2.704836 -2.165027 -0.226871

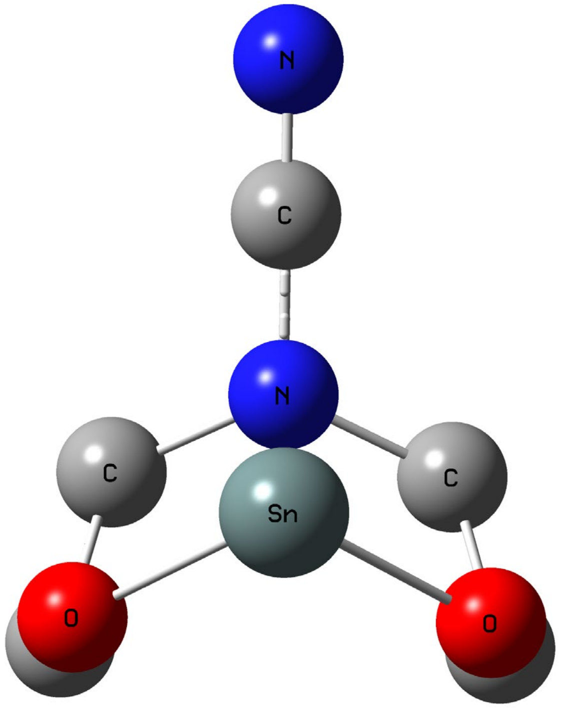

File name: tBut_boat-boat_Sn_N_ECP.log
Optimized Absolute E: -734.706657182
Optimized XYZ coordinates:
C 0.746866 0.979398 2.022940
H 1.299438 1.855034 1.654208
H 0.694335 1.083105 3.118431
C -0.712282 0.993798 1.502313
H -1.293684 0.347000 2.156979
H -1.131405 2.006442 1.586084
C -0.721199 1.605399 -0.877776
H -1.014999 1.232268 -1.858018
H -1.386443 2.442303 -0.617208
C 0.731188 2.101974 -1.000773
H 0.998145 2.727791 -0.135138
H 0.766849 2.764248 -1.880898
O 1.396725 -0.214941 1.689823
O 1.624227 1.037732 -1.169071
C -2.120534 -0.350728 -0.101187
Sn 1.307257 -0.753326 -0.261317
N -0.859747 0.491963 0.101457
C -2.035206 -1.628846 0.759525

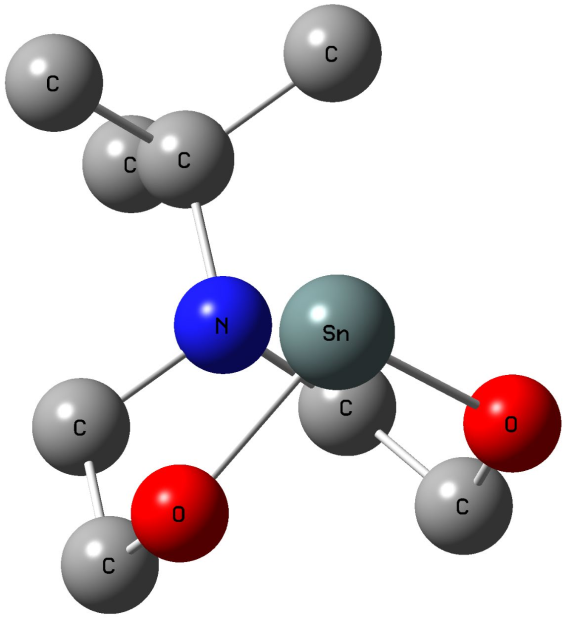

H -2.942668 -2.222399 0.606614<br>H -1.179242 -2.247519 0.467788<br>H -1.954337 -1.432072 1.831012<br>C -3.391079 0.443486 0.268460<br>H -3.479308 1.359414 -0.325524<br>H -4.279878 -0.165288 0.069303<br>H -3.411013 0.718305 1.327740<br>C -2.229314 -0.809930 -1.571231<br>H -1.282501 -1.224366 -1.936595<br>H -2.983821 -1.600007 -1.639546<br>H -2.542344 -0.011324 -2.249246	
File name: H_boat-boat_Sn_P_ECP.log<br>Optimized Absolute E: -864.059316043<br>Optimized XYZ coordinates:<br>C 0.914149 1.742912 1.092484<br>H 1.178698 1.080460 1.931500<br>H 1.056227 2.773540 1.450664<br>C 1.911897 1.502968 -0.069293<br>H 1.865291 2.340940 -0.776294<br>H 2.939686 1.433744 0.306905<br>C 1.911898 -1.502967 -0.069293<br>H 1.865292 -2.340939 -0.776294<br>H 2.939687 -1.433743 0.306905<br>C 0.914150 -1.742912 1.092484<br>H 1.178699 -1.080461 1.931500<br>H 1.056228 -2.773540 1.450664<br>O -0.425969 1.578636 0.712154<br>O -0.425968 -1.578636 0.712154<br>Sn -1.297097 -0.000000 -0.234214<br>P 1.420755 0.000000 -1.045926<br>H 2.366665 0.000000 -2.098687	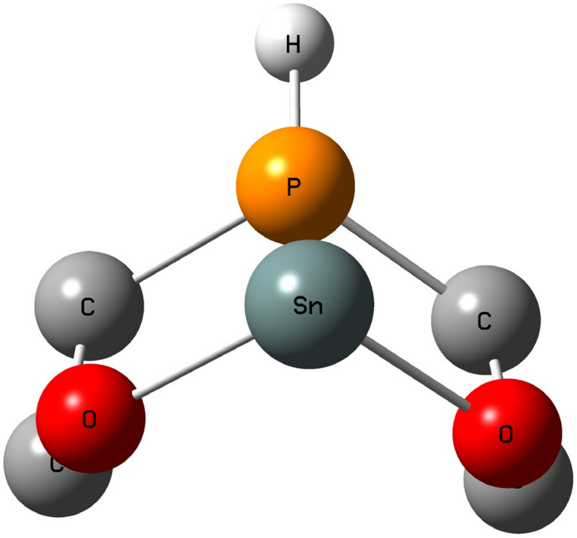

File name: OH_boat-boat_Sn_P_ECP.log<br>Optimized Absolute E: -939.290215513<br>Optimized XYZ coordinates:<br>C -0.406304 -1.731843 1.415067<br>H -0.439043 -1.069571 2.293820<br>H -0.455325 -2.761540 1.798626<br>C -1.670733 -1.484568 0.550272<br>H -1.815328 -2.322563 -0.143394<br>H -2.565862 -1.404469 1.181692<br>C -1.670739 1.484568 0.550265<br>H -1.815333 2.322559 -0.143405<br>H -2.565869 1.404470 1.181683<br>C -0.406313 1.731848 1.415062<br>H -0.439055 1.069582 2.293820<br>H -0.455334 2.761548 1.798615<br>O 0.791514 -1.573799 0.697932<br>O 0.791508 1.573798 0.697933<br>Sn 1.381730 0.000001 -0.459681<br>P -1.422422 -0.000002 -0.527095<br>O -2.673734 -0.000007 -1.616404<br>H -3.548763 -0.000007 -1.190658	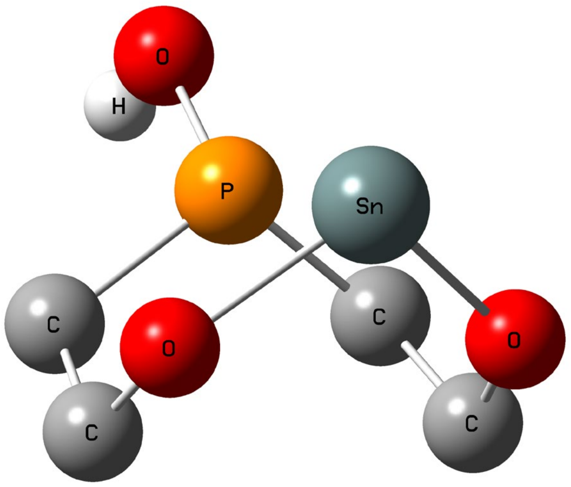
File name: CN_boat-boat_Sn_P_ECP.log<br>Optimized Absolute E: -956.297569393<br>Optimized XYZ coordinates:<br>C -0.006707 -1.734045 1.490425<br>H 0.124686 -1.068721 2.356306<br>H 0.024232 -2.763006 1.876323<br>C -1.411672 -1.504199 0.880316<br>H -1.675978 -2.341153 0.223399<br>H -2.175201 -1.428522 1.662646<br>C -1.411508 1.504494 0.880047<br>H -1.675731 2.341347 0.222970<br>H -2.175054 1.429033 1.662382<br>C -0.006533 1.734337 1.490135<br>H 0.124801 1.069138 2.356120<br>H 0.024500 2.763357 1.875870<br>O 1.029939 -1.572325 0.556489<br>O 1.030103 1.572365 0.556233<br>Sn 1.514540 -0.000097 -0.625671<br>P -1.391712 0.000040 -0.217041<br>C -3.044255 0.000078 -0.883059<br>N -4.091982 -0.000216 -1.392260	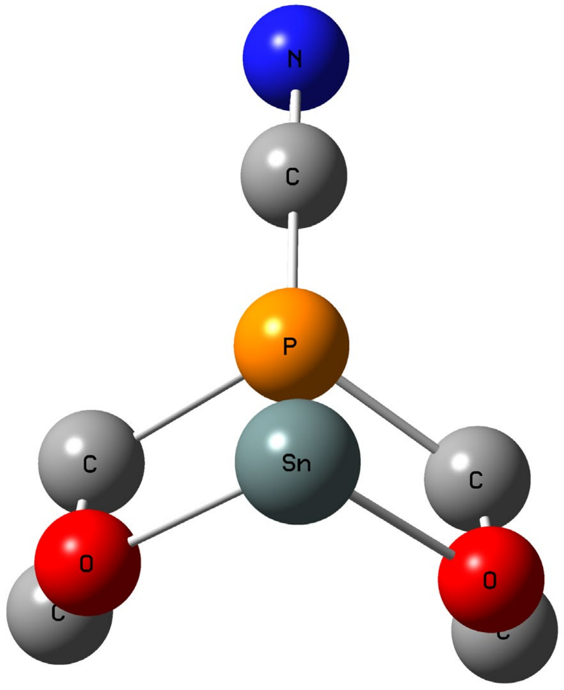

File name: tBut_boat-boat_Sn_P_ECP.log<br>Optimized Absolute E: -1021.31626677<br>Optimized XYZ coordinates:<br>C 0.841978 -1.725432 1.604845<br>H 1.142312 -1.053635 2.423942<br>H 0.939267 -2.751034 1.993060<br>C -0.660031 -1.488037 1.287065<br>H -1.044393 -2.344512 0.720938<br>H -1.240491 -1.407818 2.214639<br>C -0.660087 1.489035 1.285949<br>H -1.044334 2.345098 0.719119<br>H -1.240648 1.409564 2.213524<br>C 0.841906 1.726569 1.603698<br>H 1.142107 1.055364 2.423330<br>H 0.939225 2.752454 1.991157<br>O 1.677226 -1.582781 0.489141<br>O 1.677260 1.583026 0.488189<br>C -2.663401 -0.000178 -0.426477<br>Sn 1.726627 -0.000273 -0.804682<br>P -0.874358 0.000090 0.193567<br>C -2.863930 -1.255270 -1.300758<br>H -3.871854 -1.240075 -1.734752<br>H -2.144600 -1.293094 -2.126639<br>H -2.770558 -2.181703 -0.724608<br>C -3.676876 -0.000004 0.733545<br>H -3.571973 0.887384 1.367081<br>H -4.699161 -0.000143 0.332018<br>H -3.571866 -0.887133 1.367425<br>C -2.864103 1.254533 -1.301261<br>H -3.872016 1.239014 -1.735270<br>H -2.770881 2.181203 -0.725465<br>H -2.144761 1.292141 -2.127142	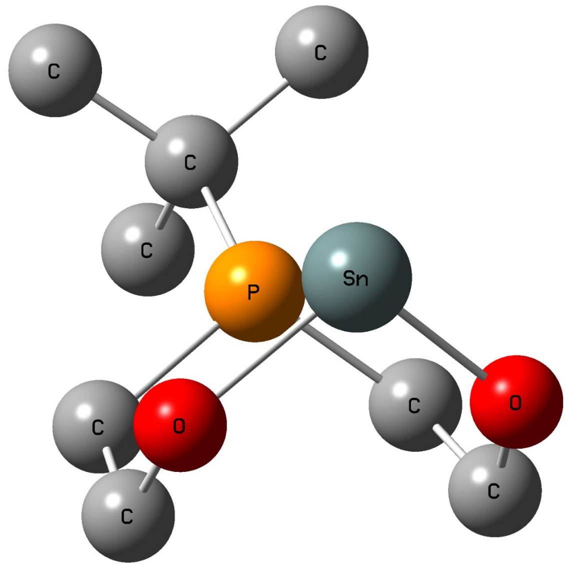

File name: H_boat-boat_Sn_As_ECP.log<br>Optimized Absolute E: -2756.11583982<br>Optimized XYZ coordinates:<br>C 0.484960 1.752406 1.358434<br>H 0.581115 1.071963 2.217810<br>H 0.516747 2.774854 1.763665<br>C 1.699727 1.565418 0.426789<br>H 1.800785 2.423259 -0.247595<br>H 2.626213 1.467799 1.002516<br>C 1.699735 -1.565414 0.426784<br>H 1.800793 -2.423253 -0.247603<br>H 2.626223 -1.467794 1.002508<br>C 0.484972 -1.752407 1.358432<br>H 0.581131 -1.071969 2.217811<br>H 0.516761 -2.774858 1.763658<br>O -0.750903 1.576881 0.707192<br>O -0.750894 -1.576879 0.707197<br>Sn -1.507910 -0.000003 -0.310396<br>As 1.435219 0.000003 -0.764155<br>H 2.781508 0.000008 -1.473580	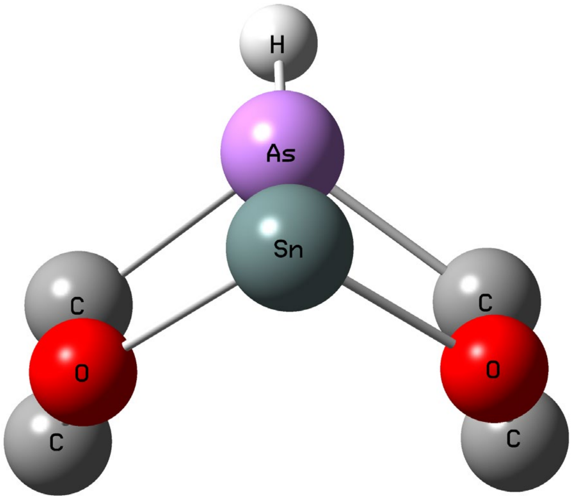
File name: OH_boat-boat_Sn_As_ECP.log<br>Optimized Absolute E: -2831.34017865<br>Optimized XYZ coordinates:<br>C -0.088556 -1.744538 1.484167<br>H -0.030051 -1.065783 2.347398<br>H -0.045412 -2.767017 1.886369<br>C -1.439275 -1.548973 0.771287<br>H -1.669506 -2.408646 0.131768<br>H -2.253192 -1.432375 1.497501<br>C -1.439316 1.548996 0.771229<br>H -1.669535 2.408648 0.131676<br>H -2.253249 1.432420 1.497427<br>C -0.088613 1.744584 1.484133<br>H -0.030138 1.065872 2.347400<br>H -0.045467 2.767083 1.886284<br>O 1.020488 -1.567140 0.626695<br>O 1.020452 1.567130 0.626700<br>Sn 1.661705 0.000005 -0.470301<br>As -1.366672 -0.000010 -0.469704<br>O -3.035939 -0.000049 -1.168683<br>H -3.693995 -0.000053 -0.453116	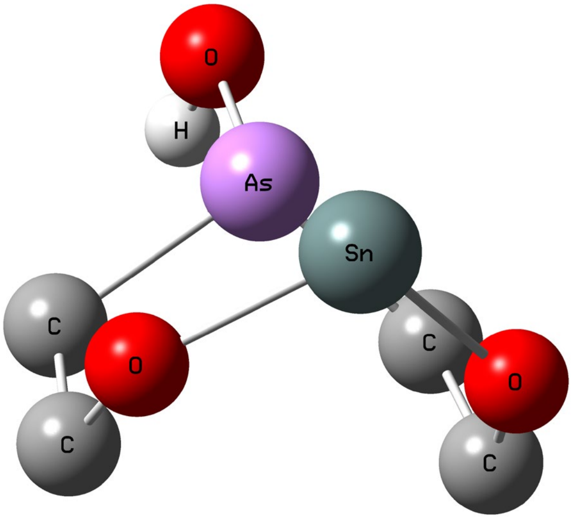

File name: CN_boat-boat_Sn_As_ECP.log<br>Optimized Absolute E: -2848.35550933<br>Optimized XYZ coordinates:<br>C -0.157345 1.739654 1.474194<br>H -0.287948 1.055298 2.323747<br>H -0.238593 2.759669 1.876015<br>C 1.253001 1.564828 0.884356<br>H 1.520344 2.423352 0.259509<br>H 2.003660 1.462842 1.674030<br>C 1.252994 -1.564882 0.884306<br>H 1.520301 -2.423395 0.259429<br>H 2.003674 -1.462943 1.673967<br>C -0.157348 -1.739672 1.474166<br>H -0.287918 -1.055335 2.323741<br>H -0.238608 -2.759695 1.875962<br>O -1.181805 1.564534 0.518577<br>O -1.181828 -1.564522 0.518574<br>As 1.316564 -0.000011 -0.350138<br>C 3.199679 -0.000009 -0.661734<br>N 4.333186 0.000063 -0.926872<br>Sn -1.864213 0.000010 -0.537054	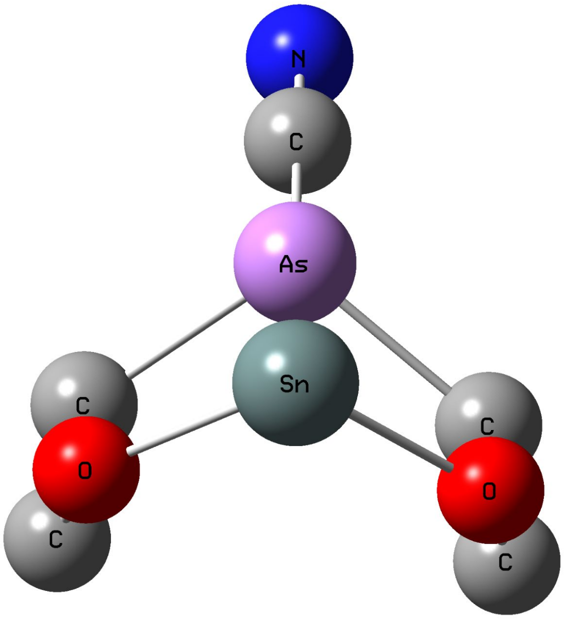
File name: tBut_boat-boat_Sn_As_ECP.log<br>Optimized Absolute E: -2913.37447125<br>Optimized XYZ coordinates:<br>C 0.931936 -1.743209 1.586457<br>H 1.188593 -1.055726 2.406980<br>H 1.062346 -2.762003 1.983148<br>C -0.560723 -1.562054 1.229303<br>H -0.917104 -2.428860 0.661531<br>H -1.168071 -1.470548 2.136612<br>C -0.560728 1.562290 1.229024<br>H -0.917035 2.428998 0.661053<br>H -1.168131 1.470997 2.136317<br>C 0.931915 1.743452 1.586222<br>H 1.188507 1.056108 2.406882<br>H 1.062339 2.762312 1.982737<br>O 1.800207 -1.580170 0.494063<br>O 1.800242 1.580187 0.493904<br>C -2.779706 -0.000046 -0.428508<br>Sn 1.975321 -0.000058 -0.777385<br>As -0.809033 0.000012 0.031248<br>C -3.052346 -1.257928 -1.269380	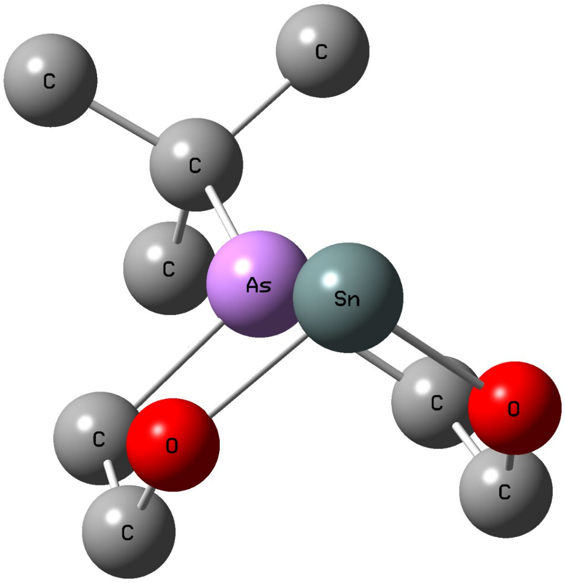

H -2.873562 -2.179071 -0.703940<br>H -4.103236 -1.268958 -1.589474<br>H -2.429908 -1.286363 -2.170979<br>C -3.052556 1.258131 -1.268859<br>H -4.103468 1.269155 -1.588884<br>H -2.873848 2.179064 -0.703051<br>H -2.430179 1.287013 -2.170486<br>C -3.647057 -0.000368 0.838146<br>H -3.471423 0.886510 1.456339<br>H -4.710540 -0.000396 0.559122<br>H -3.471280 -0.887464 1.455983	
File name: H_boat-boat_Pb_N_ECP.log<br>Optimized Absolute E: -556.021439258<br>Optimized XYZ coordinates:<br>C 1.478395 1.689785 0.834179<br>H 1.894946 1.057853 1.636483<br>H 1.835247 2.714810 1.033261<br>C 2.079337 1.265535 -0.521749<br>H 1.839055 2.037855 -1.260901<br>H 3.174970 1.185830 -0.451332<br>C 2.079346 -1.265514 -0.521771<br>H 1.839041 -2.037829 -1.260920<br>H 3.174982 -1.185814 -0.451384<br>C 1.478443 -1.689765 0.834174<br>H 1.895030 -1.057845 1.636469<br>H 1.835290 -2.714796 1.033238<br>O 0.084456 1.662131 0.817015<br>O 0.084505 -1.662094 0.817059<br>Pb -0.896712 -0.000009 -0.115843<br>N 1.493884 0.000012 -1.027744<br>H 1.539852 0.000021 -2.043147	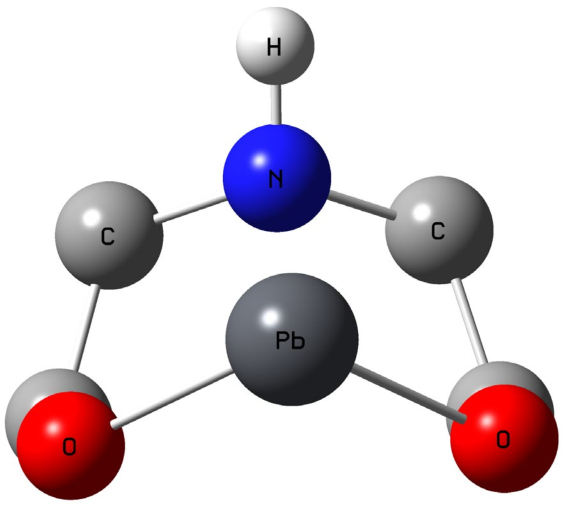

File name: OH_boat-boat_Pb_N_ECP.log<br>Optimized Absolute E: -631.180590728<br>Optimized XYZ coordinates:<br>C 1.155001 -1.673445 -1.262844<br>H 1.389355 -1.041426 -2.134798<br>H 1.466806 -2.696704 -1.533694<br>C 2.033786 -1.259296 -0.060773<br>H 1.929865 -2.018953 0.718165<br>H 3.096161 -1.183268 -0.346695<br>C 2.033762 1.259307 -0.060962<br>H 1.929961 2.019048 0.717909<br>H 3.096098 1.183207 -0.347006<br>C 1.154840 1.673377 -1.262963<br>H 1.389019 1.041237 -2.134875<br>H 1.466679 2.696581 -1.533977<br>O -0.199959 -1.649952 -0.941077<br>O -0.200076 1.650021 -0.940992<br>Pb -0.973174 -0.000020 0.195412<br>N 1.568261 0.000045 0.556542<br>O 1.890730 0.000147 1.951762<br>H 2.868591 0.000194 2.003070	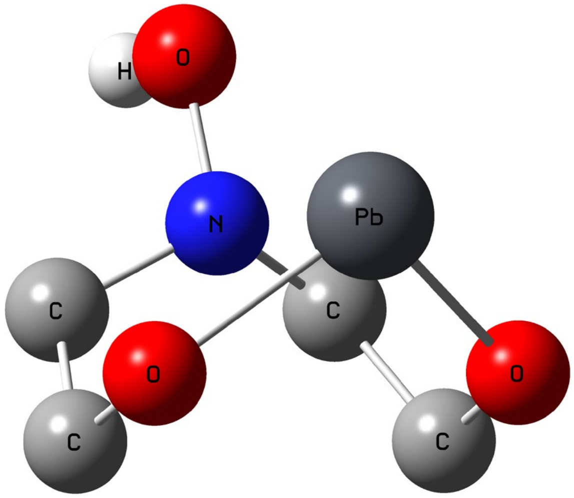
File name: CN_boat-boat_Pb_N_ECP.log<br>Optimized Absolute E: -648.241152024<br>Optimized XYZ coordinates:<br>C 0.854413 -1.528412 1.679675<br>H 0.849079 -2.425515 1.041652<br>H 1.074554 -1.878656 2.701661<br>C 2.023186 -0.605690 1.283771<br>H 2.140663 0.161338 2.053129<br>H 2.963178 -1.170061 1.209896<br>C 2.023193 -0.612842 -1.280349<br>H 2.139752 0.149872 -2.054130<br>H 2.963648 -1.176072 -1.203689<br>C 0.854977 -1.538713 -1.670434<br>H 0.850654 -2.432126 -1.027252<br>H 1.074946 -1.894665 -2.690486<br>O -0.370107 -0.861357 1.660570<br>O -0.370063 -0.872568 -1.654626<br>N 1.779592 0.116335 -0.000313<br>C 2.197930 1.402678 -0.003896<br>N 2.503692 2.528887 -0.007389<br>Pb -1.046833 0.284373 -0.000939	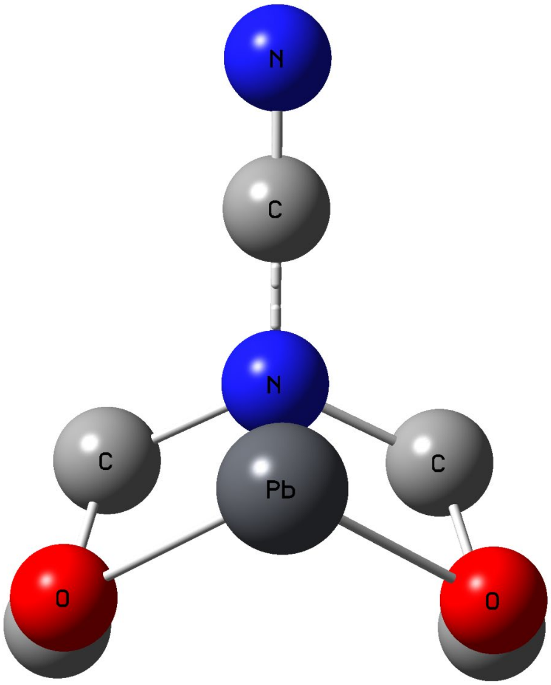

File name: tBut_boat-boat_Pb_N_ECP.log<br>Optimized Absolute E: -713.258270538<br>Optimized XYZ coordinates:<br>C 0.214156 1.474103 1.917313<br>H 0.678533 2.334647 1.413556<br>H 0.071173 1.773469 2.969707<br>C -1.204587 1.214649 1.346907<br>H -1.729685 0.596118 2.073341<br>H -1.752897 2.165300 1.271989<br>C -1.191503 1.479199 -1.095721<br>H -1.373820 0.932180 -2.020197<br>H -1.984720 2.235608 -0.985680<br>C 0.173330 2.178476 -1.253775<br>H 0.303492 2.936602 -0.464584<br>H 0.120963 2.737787 -2.204152<br>O 1.023377 0.338563 1.850534<br>O 1.233628 1.272199 -1.292386<br>C -2.347098 -0.520985 -0.076114<br>Pb 1.182441 -0.508758 -0.102549<br>N -1.226841 0.508951 0.030967<br>C -2.109942 -1.652566 0.946380<br>H -2.933529 -2.371823 0.884252<br>H -1.181150 -2.193700 0.730721<br>H -2.056861 -1.305260 1.980483<br>C -3.730492 0.120589 0.167707<br>H -3.921630 0.937099 -0.536756<br>H -4.518547 -0.627651 0.027984<br>H -3.827150 0.515423 1.183783<br>C -2.346585 -1.177671 -1.473478<br>H -1.339317 -1.490246 -1.774233<br>H -2.975480 -2.073271 -1.444649<br>H -2.751368 -0.527671 -2.253828	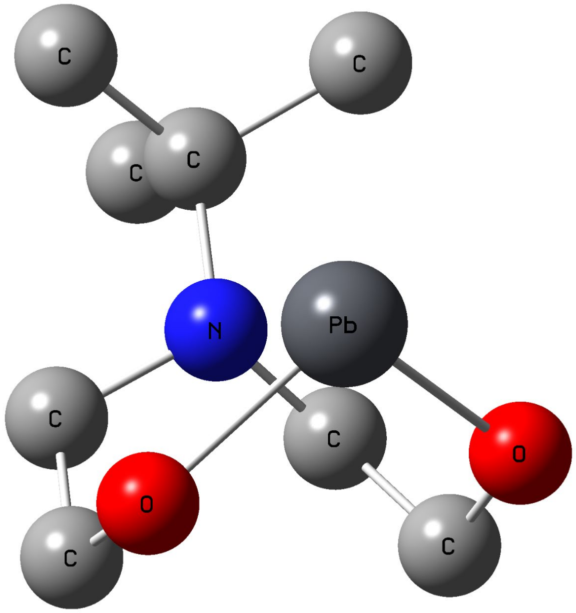

File name: H_boat-boat_Pb_P_ECP.log<br>Optimized Absolute E: -842.613866878<br>Optimized XYZ coordinates:<br>C -1.305790 -1.764001 1.089355<br>H -1.603315 -1.081649 1.902629<br>H -1.520347 -2.783801 1.448280<br>C -2.224518 -1.505688 -0.134239<br>H -2.137283 -2.340623 -0.841215<br>H -3.274281 -1.432008 0.175666<br>C -2.224517 1.505689 -0.134239<br>H -2.137282 2.340624 -0.841214<br>H -3.274279 1.432009 0.175667<br>C -1.305788 1.764001 1.089355<br>H -1.603312 1.081650 1.902629<br>H -1.520345 2.783802 1.448281<br>O 0.057250 -1.661972 0.797947<br>O 0.057252 1.661973 0.797945<br>Pb 1.051536 -0.000000 -0.137407<br>P -1.676402 0.000000 -1.076720<br>H -2.561832 0.000001 -2.181035	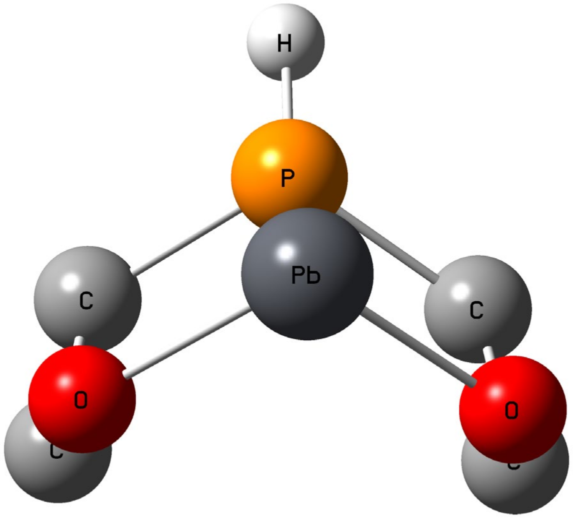
File name: OH_boat-boat_Pb_P_ECP.log<br>Optimized Absolute E: -917.845091920<br>Optimized XYZ coordinates:<br>C -0.878352 -1.751083 1.458273<br>H -0.986075 -1.068907 2.317225<br>H -1.014040 -2.769568 1.856403<br>C -2.041795 -1.485804 0.463583<br>H -2.118401 -2.321881 -0.243371<br>H -2.998631 -1.400983 0.997347<br>C -2.041800 1.485806 0.463570<br>H -2.118402 2.321878 -0.243390<br>H -2.998640 1.400989 0.997329<br>C -0.878363 1.751089 1.458267<br>H -0.986095 1.068921 2.317224<br>H -1.014050 2.769579 1.856386<br>O 0.389364 -1.655449 0.873651<br>O 0.389357 1.655445 0.873654<br>Pb 1.155938 0.000001 -0.274001<br>P -1.687695 -0.000003 -0.582019<br>O -2.835097 -0.000010 -1.782706<br>H -3.744307 -0.000011 -1.435737	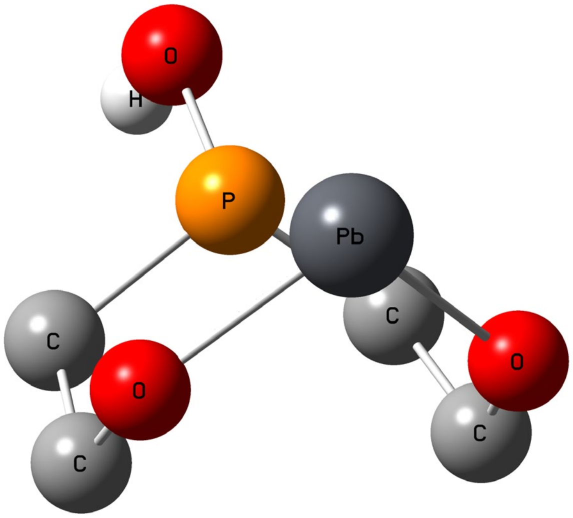

File name: CN_boat-boat_Pb_P_ECP.log<br>Optimized Absolute E: -934.852726352<br>Optimized XYZ coordinates:<br>C 0.492488 1.758093 1.617765<br>H 0.434283 1.075449 2.480070<br>H 0.542162 2.777387 2.031833<br>C 1.827803 1.508094 0.868733<br>H 2.031140 2.340355 0.184593<br>H 2.666711 1.426785 1.569490<br>C 1.827771 -1.508335 0.868458<br>H 2.031104 -2.340460 0.184156<br>H 2.666669 -1.427173 1.569243<br>C 0.492432 -1.758452 1.617407<br>H 0.434223 -1.075998 2.479864<br>H 0.542077 -2.777837 2.031253<br>O -0.633732 1.655906 0.794544<br>O -0.633762 -1.656071 0.794175<br>Pb -1.263267 0.000057 -0.409862<br>P 1.690450 -0.000017 -0.213661<br>C 3.254277 -0.000112 -1.067165<br>N 4.236295 0.000383 -1.693972	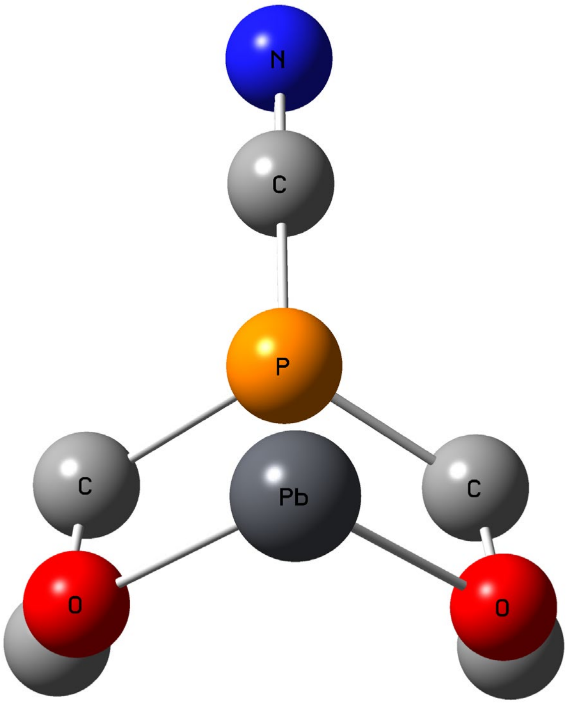
File name: tBut_boat-boat_Pb_P_ECP.log<br>Optimized Absolute E: -999.870298956<br>Optimized XYZ coordinates:<br>C 0.324091 -1.819460 -1.746373<br>H 0.544609 -2.649429 -1.055312<br>H 0.343639 -2.252515 -2.760523<br>C -1.131837 -1.336042 -1.491922<br>H -1.456086 -0.730127 -2.346231<br>H -1.809945 -2.195264 -1.410225<br>C -1.132080 -1.340515 1.487937<br>H -1.455889 -0.737021 2.344125<br>H -1.810536 -2.199226 1.403826<br>C 0.323705 -1.825235 1.740613<br>H 0.543743 -2.653228 1.047024<br>H 0.343372 -2.261325 2.753459<br>O 1.279320 -0.804991 -1.665277<br>O 1.279277 -0.810873 1.662291<br>C -2.964072 0.548338 0.000775<br>Pb 1.508908 0.546919 0.000912<br>P -1.234931 -0.229345 -0.000327<br>C -3.088192 1.438969 -1.252427	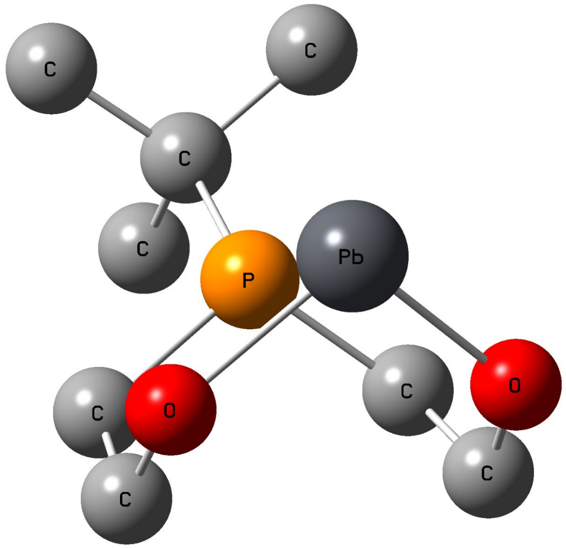

H -4.054387 1.959457 -1.236628<br>H -2.299572 2.199229 -1.288750<br>H -3.044756 0.858815 -2.179953<br>C -4.077474 -0.516137 -0.000518<br>H -4.029607 -1.157830 0.885799<br>H -5.059860 -0.024811 0.000105<br>H -4.029618 -1.155607 -0.888447<br>C -3.087927 1.435667 1.256364<br>H -4.054164 1.956123 1.242208<br>H -3.044189 0.853075 2.182350<br>H -2.299348 2.195878 1.294457	
File name: H_boat-boat_Pb_As_ECP.log<br>Optimized Absolute E: -2734.67063426<br>Optimized XYZ coordinates:<br>C -0.920952 -1.776957 1.378878<br>H -1.071725 -1.077512 2.216845<br>H -1.034797 -2.790016 1.798283<br>C -2.051271 -1.570148 0.347533<br>H -2.101239 -2.422904 -0.339123<br>H -3.023418 -1.466381 0.841773<br>C -2.051270 1.570149 0.347534<br>H -2.101239 2.422905 -0.339122<br>H -3.023417 1.466382 0.841774<br>C -0.920951 1.776957 1.378878<br>H -1.071724 1.077512 2.216845<br>H -1.034796 2.790016 1.798283<br>O 0.366670 -1.662626 0.836925<br>O 0.366671 1.662626 0.836924<br>Pb 1.252188 -0.000000 -0.181222<br>As -1.680881 0.000000 -0.807117<br>H -2.948066 0.000001 -1.648270	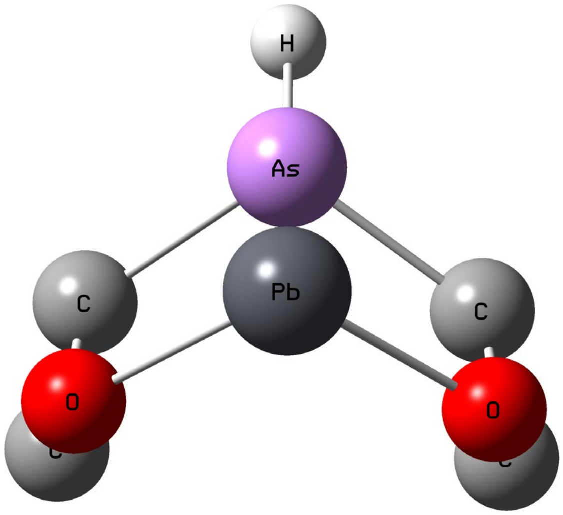

File name: OH_boat-boat_Pb_As_ECP.log
Optimized Absolute E: -2809.89500634
Optimized XYZ coordinates:
C 0.563727 1.772257 1.566540
H 0.575900 1.077885 2.421397
H 0.604822 2.787012 1.993299
C 1.837724 1.555220 0.724133
H 2.009811 2.407309 0.056597
H 2.719928 1.434057 1.365222
C 1.837723 -1.555219 0.724134
H 2.009811 -2.407309 0.056599
H 2.719927 -1.434056 1.365224
C 0.563726 -1.772256 1.566541
H 0.575898 -1.077883 2.421397
H 0.604821 -2.787011 1.993301
O -0.625195 1.652769 0.824836
O -0.625196 -1.652769 0.824836
Pb -1.386838 0.000000 -0.296468
As 1.625827 -0.000000 -0.487005
O 3.187589 -0.000001 -1.400188
H 3.932520 -0.000001 -0.775444

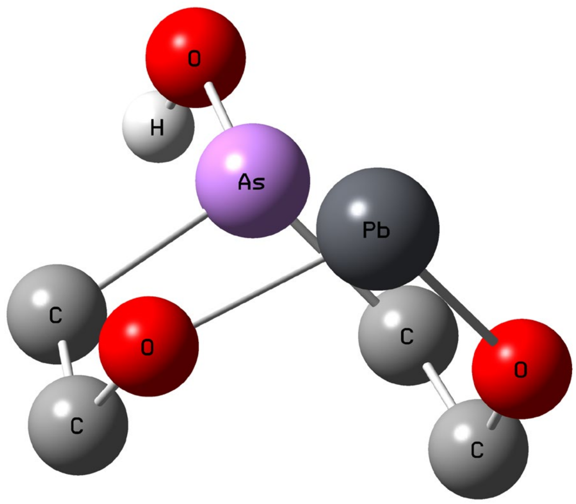

File name: CN_boat-boat_Pb_As_ECP.log
Optimized Absolute E: -2826.91032287
Optimized XYZ coordinates:
C -0.299122 -1.769479 1.618071
H -0.219318 -1.069383 2.463384
H -0.291194 -2.781842 2.051376
C -1.659990 -1.570870 0.921857
H -1.888812 -2.421442 0.271290
H -2.471316 -1.457253 1.647738
C -1.660064 1.570553 0.922176
H -1.888965 2.421243 0.271793
H -2.471358 1.456731 1.648061
C -0.299197 1.769136 1.618402
H -0.219325 1.068884 2.463578
H -0.291328 2.781418 2.051899
O 0.795792 -1.652504 0.745090
O 0.795706 1.652399 0.745377
Pb 1.524787 0.000064 -0.381233
As -1.592874 -0.000036 -0.297909
C -3.410697 -0.000087 -0.866700
N -4.497666 0.000419 -1.283370

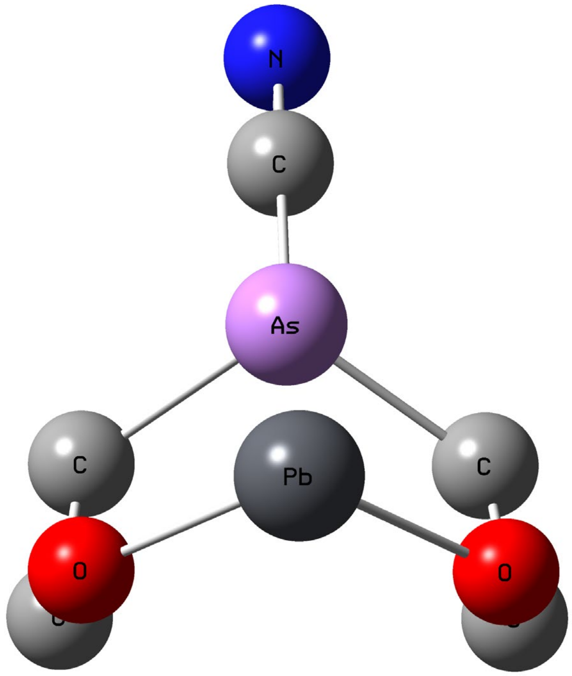

File name: tBut_boat-boat_Pb_As_ECP.log<br>Optimized Absolute E: -2891.92907089<br>Optimized XYZ coordinates:<br>C -0.435388 1.766345 1.791002<br>H -0.627551 1.059679 2.614759<br>H -0.492106 2.774932 2.234145<br>C 1.016572 1.565883 1.295713<br>H 1.326311 2.428896 0.695480<br>H 1.705021 1.469729 2.143142<br>C 1.016572 -1.565880 1.295717<br>H 1.326312 -2.428894 0.695487<br>H 1.705021 -1.469723 2.143146<br>C -0.435389 -1.766342 1.791006<br>H -0.627552 -1.059673 2.614761<br>H -0.492107 -2.774928 2.234151<br>O -1.403563 1.665423 0.785858<br>O -1.403563 -1.665421 0.785861<br>C 3.072174 -0.000001 -0.553743<br>Pb -1.722809 -0.000001 -0.538650<br>As 1.152258 0.000000 0.084620<br>C 3.268639 1.257801 -1.415687<br>H 4.286224 1.268699 -1.829898<br>H 2.567020 1.286717 -2.257294<br>H 3.141749 2.179029 -0.836515<br>C 3.268638 -1.257802 -1.415687<br>H 4.286222 -1.268701 -1.829899<br>H 3.141749 -2.179031 -0.836515<br>H 2.567018 -1.286719 -2.257294<br>C 4.051446 -0.000001 0.628577<br>H 5.085187 0.000000 0.253959<br>H 3.932380 0.886916 1.260059<br>H 3.932381 -0.886919 1.260057	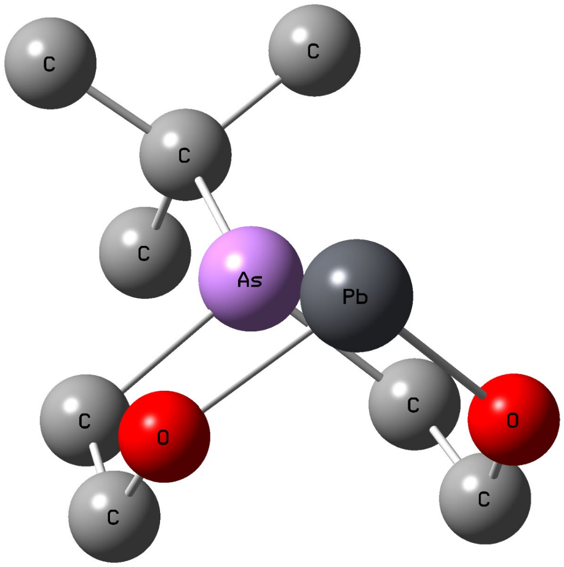

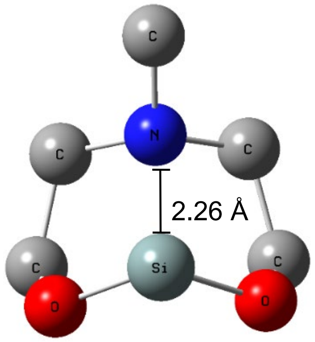

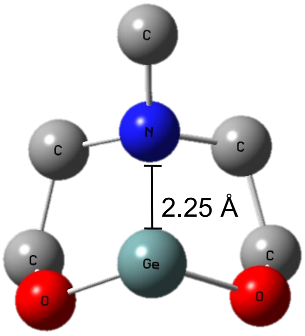

(a) (b)

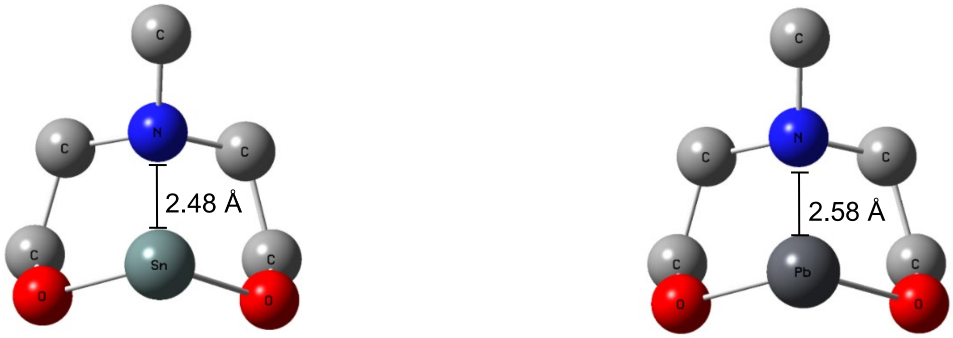

(c) (d)

Figure S1. The boat-boat optimized geometries for quasimetallatranes compounds containing a nitrogen atom and a metal atom are shown: (a) silicon, (b) germanium, (c) tin, and (d) lead. The 6-31G* level of theory was used for the germanium- and silicon-containing compounds and the MDF ECP was used for the tin- and lead-containing compounds

Table S2. Dative bond distances (Å) of the boat-boat congener with various R groups. The 6-31G* level of theory was used for the germanium- and silicon-containing compounds and the MDF ECP was used for the tin- and lead-containing compounds.

	R Groups				
Bond	H	Me	tBut	OH	CN
Si←N	2.24	2.26	2.31	2.31	3.03
Si←P	3.21	3.10	2.76	3.33	3.32
Si←As	3.25	3.18	2.78	3.27	3.25
Ge←N	2.25	2.25	2.27	2.29	2.64
Ge←P	2.70	2.66	2.61	2.68	2.84
Ge←As	2.84	2.75	2.70	2.91	3.03
Sn←N	2.47	2.48	2.53	3.32	2.78
Sn←P	2.84	2.80	2.79	2.80	2.93
Sn←As	2.98	2.93	2.90	3.03	4.07
Pb←N	2.56	2.58	2.62	2.57	3.38
Pb←P	2.89	2.86	2.85	2.86	2.96
Pb←As	3.24	2.96	2.94	3.02	3.12

Table S3. Molecular orbital numbers and energies for the HOMO, LUMO, and dative bond-containing MOs for each compound studied. The ΔE values were calculated by the difference in energy between the LUMO and the HOMO (i.e. L – H). Calculations were performed using B3LYP/6-31G* for silicon- and germanium-containing compounds. For tin- and lead-containing compounds the MDF ECPs were used along with the corresponding basis sets.

	HOMO		LUMO		Dative Bond		
Compound	MO	Energy (au)	MO	Energy (au)	MO	Energy (au)	Δ Energy (au)
Si←N	39	-0.201	40	0.014	37	-0.283	0.215
					35	-0.301	
					34	-0.330	
Si←P	43	-0.230	44	-0.023	42	-0.239	0.207
					39	-0.313	
					37	-0.326	
Si←As	52	-0.234	53	-0.028	51	-0.240	0.206
					47	-0.314	
Ge←N	48	-0.217	49	-0.002	44	-0.288	0.219
					43	-0.332	
Ge←P	52	-0.229	53	-0.008	50	-0.243	0.221
					48	-0.281	
					47	-0.329	
Ge←As	61	-0.236	62	-0.024	59	-0.244	0.212
					57	-0.285	
					55	-0.325	
					54	0.334	
Sn←N	43	-0.230	44	-0.033	39	-0.280	0.197
					38	-0.324	
Sn←P	47	-0.233	48	-0.026	45	-0.241	0.206
					43	-0.267	
					42	-0.322	
Sn←As	56	-0.236	57	-0.038	55	-0.238	0.198
					52	-0.270	
					50	-0.318	
					49	-0.332	
Pb←N	43	-0.223	44	-0.041	39	-0.272	0.182
					26	-0.513	
Pb←P	47	-0.221	48	-0.033	46	-0.229	0.189
					43	-0.256	
					41	-0.333	
Pb←As	56	-0.224	57	-0.042	55	-0.229	0.182
					52	-0.258	
					49	-0.334	

Table S4. Electronic composition of dative bonds obtained by **Natural Bond Orbital (NBO)** analysis. The silicon- and germanium-containing compounds are calculated at B3LYP/6-31G* level of theory. Tin and lead-containing compounds are calculated with the MDF ECP. The covalency ratio (χ) is a dimensionless ratio measuring the degree of electronic overlap in a chemical bond.

		**M hybrid orbital composition**					**Y hybrid orbital composition**				
**Metal**	**Geometry**	**%M**	**%s**	**%p**	**%d**	**%f**	**%Donor**	**%s**	**%p**	**%d**	**χ**
Si←N	Boat-Boat	7.33	3.18	94.89	1.93		92.67	13.96	86.04	0.00	0.76
	Boat-Chair	7.48	3.01	95.29	1.70		92.52	13.86	86.13	0.00	0.75
	Chair-Chair	7.70	3.13	95.17	1.70		92.30	13.81	86.19	0.00	0.77
	Crown	-	-	-	-		-	-	-	-	-
Si←P	Boat-Boat	-	-	-	-		-	-	-	-	0.47
	Boat-Chair	3.71	8.27	61.01	30.71		96.29	23.37	76.63	0.00	0.53
	Chair-Chair	-	-	-	-		-	-	-	-	0.20
	Crown	-	-	-	-		-	-	-	-	-0.25
Si←As	Boat-Boat	-	-	-	-		-	-	-	-	0.46
	Boat-Chair	-	-	-	-		-	-	-	-	0.51
	Chair-Chair	-	-	-	-		-	-	-	-	0.39
	Crown	-	-	-	-		-	-	-	-	-
Ge←N	Boat-Boat	8.09	2.42	97.37	0.12		91.91	13.78	86.22	0.00	0.79
	Boat-Chair	8.22	2.30	97.52	0.18		91.78	13.77	86.23	0.00	0.78
	Chair-Chair	8.37	2.41	97.40	0.19		91.63	13.57	86.43	0.00	0.79
	Crown	-	-	-	-		-	-	-	-	0.00
Ge←P	Boat-Boat	13.53	2.04	97.29	0.67		86.47	26.03	73.93	0.04	0.74
	Boat-Chair	13.21	1.72	97.72	0.56		86.79	25.99	73.98	0.03	0.72
	Chair-Chair	13.54	1.67	97.78	0.55		86.46	26.15	73.82	0.03	0.74
	Crown	-	-	-	-		-	-	-	-	-0.44
Ge←As	Boat-Boat	12.13	1.53	97.76	0.70		87.87	29.98	70.01	0.01	0.75
	Boat-Chair	11.98	1.31	98.10	0.59		88.02	29.53	70.46	0.01	0.73
	Chair-Chair	12.20	1.26	98.15	0.59		87.80	29.55	70.44	0.01	0.74
	Crown	-	-	-	-		-	-	-	-	-0.50
Sn←N	Boat-Boat	7.03	1.75	97.47	0.47	0.31	92.97	11.00	89.00	0.00	0.77
	Boat-Chair	7.14	1.73	97.56	0.40	0.30	92.86	11.21	88.78	0.00	0.76
	Chair-Chair	7.27	1.79	97.46	0.43	0.31	92.73	11.01	88.99	0.00	0.77
Sn←P	Boat-Boat	12.05	1.90	97.08	0.71	0.31	87.95	23.51	76.45	0.04	0.77
	Boat-Chair	11.75	1.63	97.43	0.67	0.27	88.25	23.42	76.54	0.04	0.75
	Chair-Chair	11.97	1.54	97.42	0.75	0.29	88.03	23.53	76.43	0.04	0.77
Sn←As	Boat-Boat	6.33	8.34	59.43	25.58	6.58	93.67	26.5	73.49	0.01	0.76
	Boat-Chair	7.80	3.41	69.77	23.36	3.46	92.20	26.06	73.93	0.01	0.74
	Chair-Chair	7.49	6.22	76.81	11.06	5.84	92.51	26.01	73.98	0.01	0.75
Pb←N	Boat-Boat	4.61	4.05	74.08	14.53	7.22	95.39	9.87	90.13	0.01	0.71
	Boat-Chair	5.44	2.85	81.27	10.26	5.62	94.56	10.12	89.88	0.01	0.71

	Chair-Chair	6.93	1.1	98.24	0.27	0.38	93.07	10.55	89.45	0.01	0.72
Pb←P	Boat-Boat	11.75	1.28	97.84	0.43	0.45	88.25	22.61	77.35	0.04	0.74
	Boat-Chair	11.51	1.10	98.07	0.43	0.40	88.49	22.58	77.39	0.03	0.72
	Chair-Chair	11.79	1.07	98.01	0.49	0.42	88.21	22.66	77.3	0.04	0.74
Pb←As	Boat-Boat	6.68	6.62	61.37	22.57	9.24	93.32	25.62	74.37	0.01	0.74
	Boat-Chair	8.05	2.67	71.56	20.78	4.99	91.95	25.22	74.77	0.01	0.72
	Chair-Chair	8.56	4.07	82.89	6.39	6.51	91.44	25.18	74.81	0.01	0.74

Table S5. The table below summarizes the electronic composition of dative bonds in various conformations of compounds containing silicon (Si), germanium (Ge), tin (Sn), and lead (Pb) based on **IBO (Intrinsic Bond Orbital)** analysis performed in the NWChem software package. The percentage contributions of the metal (M) and the donor atom (Y) to the dative bond are presented along with the bond energies. The silicon- and germanium-containing compounds are calculated at the B3LYP/6-31G* level of theory and STO-6G basis were used for the IAO, while tin and lead-containing compounds are calculated with the Stuttgart MDF ECP and it is associated basis set.

M←Y	Geometry	%M	%Donor	Energy (Au)
Si←N	Boat-Boat	13	83	-0.444557
	Boat-Chair	13	83	-0.437388
	Chair-Chair	14	83	-0.437635
	Crown	-	-	-
Si←P	Boat-Boat	3	96	-0.3852
	Boat-Chair	4	94	-0.393666
	Chair-Chair	-	-	-
	Crown	-	-	-
Si←As	Boat-Boat	-	-	-
	Boat-Chair	1	98	-0.438416
	Chair-Chair	-	-	-
	Crown	-	-	-
Ge←N	Boat-Boat	14	82	-0.441195
	Boat-Chair	14	82	-0.435225
	Chair-Chair	15	81	-0.433849
	Crown	-	-	-
Ge←P	Boat-Boat	13	84	-0.440458
	Boat-Chair	12	86	-0.432133
	Chair-Chair	13	85	-0.438162
	Crown	-	-	-
Ge←As	Boat-Boat	5	94	-0.462182
	Boat-Chair	6	93	-0.462499
	Chair-Chair	5	94	-0.457267
	Crown	-	-	-
Sn←N	Boat-Boat	16	80	-0.429237
	Boat-Chair	16	80	-0.42688
	Chair-Chair	17	80	-0.431095
	Crown	-	-	-
Sn←P	Boat-Boat	20	78	-0.457979
	Boat-Chair	20	78	-0.45522
	Chair-Chair	20	78	-0.458928
	Crown	-	-	-
Sn←As	Boat-Boat	13	85	-0.517876
	Boat-Chair	13	85	-0.515784
	Chair-Chair	14	85	-0.519194
	Crown	-	-	-
Pb←N	Boat-Boat	18	78	-0.402079
	Boat-Chair	19	77	-0.401609
	Chair-Chair	19	77	-0.403162

	Crown	-	-	-
Pb←P	Boat-Boat	24	74	-0.448663
	Boat-Chair	23	75	-0.447803
	Chair-Chair	24	74	-0.451177
	Crown	-	-	-
Pb←As	Boat-Boat	15	83	-0.518064
	Boat-Chair	15	83	-0.517407
	Chair-Chair	16	82	-0.521075
	Crown	-	-	-

Table S6. Second Order Perturbation Theory analysis of the Fock matrix in the NBO basis highlighting interactions between Y lone pairs and virtual M natural orbitals. LP = lone pair orbital, LP* = lone pair virtual orbital and BD* = bonding virtual orbital. The silicon- and germanium-containing compounds are calculated at the B3LYP/6-31G* level of theory and STO-6G basis were used for the IAO, while tin and lead-containing compounds are calculated with the Stuttgart MDF ECP and it is associated basis set.

M←Y	Geometry	Donor orbital	Donor orbital occupation	Acceptor orbital	Acceptor orbital occupation	Energy (kcal/mol)
Si←N	Boat-Boat					
	Boat-Chair					
	Chair-Chair					
Si←P	Boat-Boat					
	Boat-Chair					
	Chair-Chair	LP	1	LP*	2	0.85
Si←As	Boat-Boat	LP	1	LP*	2	3.85
	Boat-Chair	LP	1	BD*	2	3.16
	Chair-Chair	LP	1	LP*	2	3.04
Ge←N	Boat-Boat					
	Boat-Chair					
	Chair-Chair					
Ge←P	Boat-Boat					
	Boat-Chair					
	Chair-Chair					
Ge←As	Boat-Boat	LP	1	LP*	2	19.03
	Boat-Chair					
	Chair-Chair					
Sn←N	Boat-Boat					
	Boat-Chair					
	Chair-Chair					
Sn←P	Boat-Boat					
	Boat-Chair					
	Chair-Chair					
Sn←As	Boat-Boat					
	Boat-Chair	LP	1	LP*	2	23.69
	Chair-Chair					
Pb←N	Boat-Boat					
	Boat-Chair	LP	1	LP*	2	22.43
	Chair-Chair					
Pb←P	Boat-Boat					
	Boat-Chair					
	Chair-Chair					
Pb←As	Boat-Boat					
	Boat-Chair	LP	1	LP*	2	25.01
	Chair-Chair					

Table S7: The following table shows the M←Y distance in Angstroms and the relative energy in kcal/mol for each conformation. The B3LYP/6-31G* level of theory was used for silicon- and germanium-containing systems while B3LYP/LanL2DZ was used for tin- and lead-containing systems. The following ECPs were used for each type of metal: ECP10MWB for Silicon, ECP10MDF for Germanium, ECP28MDF for Tin, and ECP60MDF for Lead. A dash (-) indicates the conformation was unobtainable using this method. Please note that LanL2DZ already incorporates an ECP treatment.

		With 6-31G*/LanL2DZ Basis Sets		With ECPs and their Corresponding Basis Sets	
Bond	Conformation	M←Y	Rel E	M←Y	Rel E
Si←N	Boat-Boat	2.260	0.00	2.279	0.00
	Boat-Chair	2.270	0.45	2.290	0.59
	Crown	3.605	10.12	3.636	12.39
	Chair-Chair	2.240	6.19	2.261	6.97
Si←P	Boat-Boat	3.096	0.73	2.870	1.13
	Boat-Chair	2.982	0.00	2.880	0.00
	Crown	4.322	2.48	4.415	3.94
	Chair-Chair	3.559	3.14	3.370	4.45
Si←As	Boat-Boat	3.185	0.64	3.304	1.18
	Boat-Chair	3.109	0.00	3.157	0.00
	Crown	4.439	4.75	4.513	3.77
	Chair-Chair	3.305	2.91	3.507	3.03

Ge←N	Boat-Boat	2.248	0.19	2.338	0.00
	Boat-Chair	2.268	0.00	2.346	0.50
	Crown	3.552	17.02	3.547	13.32
	Chair-Chair	2.251	5.47	2.321	6.18
Ge←P	Boat-Boat	2.661	1.36	2.694	0.69
	Boat-Chair	2.694	0.00	2.753	0.00
	Crown	4.479	8.38	4.494	6.94
	Chair-Chair	2.663	4.60	2.715	4.42
Ge←As	Boat-Boat	2.755	1.77	2.935	1.40
	Boat-Chair	2.789	0.00	2.955	0.00
	Crown	4.579	10.23	4.598	6.40
	Chair-Chair	2.763	4.26	2.980	4.06
Sn←N	Boat-Boat	2.471	0.00	2.483	0.00
	Boat-Chair	2.467	1.37	2.480	0.75
	Crown	3.718	10.52	-	-
	Chair-Chair	-	-	2.457	6.28
Sn←P	Boat-Boat	2.996	0.00	2.804	0.00

	Boat-Chair	3.058	0.51	2.836	0.23
	Crown	4.218	1.75	4.226	10.16
	Chair-Chair	3.108	4.20	2.812	4.07
Sn←As	Boat-Boat	3.221	0.45	2.932	0.37
	Boat-Chair	3.600	0.32	2.964	0.00
	Crown	4.236	0.00	4.121	7.92
	Chair-Chair	-	-	2.943	3.54
Pb←N	Boat-Boat	2.560	0.00	2.583	0.00
	Boat-Chair	2.555	1.15	2.578	0.53
	Crown	3.556	9.91	-	-
	Chair-Chair	2.556	1.16	2.565	5.81
Pb←P	Boat-Boat	3.006	0.00	2.862	0.00
	Boat-Chair	3.057	0.68	2.892	0.09
	Crown	-	-	-	-
	Chair-Chair	3.077	4.44	2.866	3.69
Pb←As	Boat-Boat	3.179	0.00	2.963	0.44
	Boat-Chair	3.249	0.34	2.991	0.00

	Crown	4.624	5.08	-	-
	Chair-Chair	-	-	2.967	3.33

Table S3: The following table shows the frequency data obtained for each one of the boat-boat systems. The B3LYP/6-31G* level of theory was used for silicon- and germanium-containing systems while B3LYP/LanL2DZ was used for tin- and lead-containing systems. Bolded data is shown in Figure 5 in the original manuscript. Please note that LanL2DZ already incorporates an ECP treatment.

Bond	Frequency	Freq Scaled	M displacement	Y displacement	IR intensity
Si ← N	161.7756	155.97	**0.25**	**-0.11**	4.9718
	233.2515	224.88	**0.22**	-0.04	**15.9169**
	289.6056	279.21	0.05	-0.08	16.5773
	377.1773	363.64	0.00	**-0.17**	12.9233
	439.5076	423.73	0.01	**-0.11**	16.6905
	487.023	469.54	-0.05	**-0.23**	**12.2819**
	626.1758	603.70	-0.02	0.06	10.3958
	676.7756	652.48	0.05	0.02	**65.6774**
	760.0369	732.75	-0.01	-0.08	10.3877
	911.4668	878.75	-0.01	-0.05	30.6172
	1004.3938	968.34	0.01	0.14	38.9816
	1081.884	1043.04	0.00	0.15	21.3219
Si ← P	44.011	42.43	**0.34**	**-0.16**	**11.2286**
	186.2149	179.53	**0.14**	-0.02	**8.0998**
	233.8387	225.44	0.01	-0.07	0.4639
	264.8195	255.31	0.03	**-0.13**	1.3051
	329.1073	317.29	-0.04	-0.08	17.8558
	412.2242	397.43	**-0.12**	-0.06	**19.7587**
	624.4508	602.03	-0.06	-0.04	**6.8895**
	681.594	657.12	-0.08	-0.07	28.0171

	705.4072	680.08	0.05	-0.23	9.9021
Si ← As	69.286	66.80	**0.36**	**-0.13**	**5.4668**
	174.4131	168.15	**0.19**	-0.01	**6.8566**
	223.7649	215.73	-0.01	**0.1**	1.7798
	303.3819	292.49	-0.05	-0.05	17.6846
	403.1937	388.72	**-0.1**	-0.03	**12.6062**
	559.7189	539.62	-0.01	**-0.1**	**8.4671**
	587.9773	566.87	-0.05	0.01	5.8833
	682.4138	657.92	-0.09	0.01	29.5679
Ge ← N	147.971	142.66	**-0.14**	**0.11**	**4.1191**
	184.3454	177.73	0.05	-0.03	7.653
	265.3632	255.84	0.05	-0.06	9.0207
	287.5361	277.21	-0.02	0.05	4.471
	336.8281	324.74	-0.03	0.08	10.453
	425.3024	410.03	-0.02	**-0.1**	**5.9501**
	428.7403	413.35	0.01	**0.2**	3.1779
	442.6588	426.77	-0.01	**0.15**	**13.7075**
	576.2254	555.54	-0.01	0.08	43.3892
	586.6791	565.62	0	-0.05	71.8231
	756.6085	729.45	0	0.11	10.867
	998.8627	963.00	0	0.12	34.3086
	1044.0421	1006.56	0	-0.06	8.7919
	1080.3969	1041.61	0	0.1	22.8125
Ge ← P	97.7568	94.25	**0.13**	**-0.09**	**9.7665**
	192.8148	185.89	**0.08**	-0.01	**12.7328**
	209.8246	202.29	-0.05	0.05	5.2709

	282.9947	272.84	-0.06	**0.21**	**0.917**
	322.6891	311.10	-0.05	-0.04	14.2778
	331.9727	320.05	-0.04	-0.08	16.2453
	639.682	616.72	0	-0.05	1.5947
	728.7946	702.63	0	-0.22	9.0436
Ge ← As	75.537	72.83	**0.15**	**-0.09**	**10.7959**
	178.2202	171.82	**0.14**	-0.07	**12.7038**
	204.5871	197.24	-0.03	0.08	0.94
	223.1742	215.16	-0.02	0.09	5.0725
	291.3585	280.90	-0.07	-0.07	18.3271
	575.8939	555.22	0.01	**-0.11**	**9.9907**
Sn ← N	133.6017	128.3912337	**-0.12**	**0.16**	9.5938
	238.3491	229.0534851	0.06	**-0.1**	**21.3823**
	312.1477	299.9739397	0.02	-0.06	13.8497
	402.8341	387.1235701	0.03	**0.24**	6.865
	437.0591	420.0137951	0	**0.17**	**16.1153**
	486.1953	467.2336833	0.04	**-0.18**	**33.4406**
	737.9036	709.1253596	0	**0.13**	13.7912
	897.7722	862.7590842	0	-0.07	28.2746
	999.9368	960.9392648	0	-0.14	42.0535
	1161.277	1115.987197	0	-0.08	6.3055
Sn ← P	73.0555	70.2063355	**0.11**	**-0.13**	**16.1818**
	184.5181	177.3218941	0.05	-0.06	9.4904
	256.4309	246.4300949	-0.04	**0.16**	4.1228
	284.7232	273.6189952	0.03	**0.11**	**18.3833**
	312.3967	300.2132287	0.02	**0.09**	**20.9893**

	438.6406	421.5336166	-0.05	0.01	29.4391
	627.5764	603.1009204	0	-0.05	2.0625
	696.6672	669.4971792	0	-0.24	3.5026
Sn ← As	49.495	47.564695	**0.15**	**-0.16**	**14.1113**
	146.5709	140.8546349	-0.06	0.03	11.0517
	171.2698	164.5902778	-0.03	0.06	2.5234
	206.4593	198.4073873	-0.01	0.08	6.4131
	262.8373	252.5866453	0.05	**0.09**	**31.3107**
	576.0535	553.5874135	0.01	**-0.11**	**6.4239**
Pb ← N	114.8436	110.3646996	**-0.08**	**0.17**	**10.6176**
	164.3261	157.9173821	0	0.05	13.9281
	215.4419	207.0396659	0.05	**-0.11**	**17.4962**
	381.6564	366.7718004	0.02	**0.23**	**9.6633**
	431.2762	414.4564282	0	**0.16**	**14.1427**
	452.1248	434.4919328	0.02	**-0.24**	**26.4459**
	735.5793	706.8917073	0	0.14	13.4656
	892.8184	857.9984824	0	-0.06	28.7394
	999.9427	960.9449347	0	-0.13	40.159
	1158.0977	1112.93189	0	-0.09	7.2092
Pb ← P	74.307	71.409027	-0.07	**0.14**	**15.3909**
	174.9117	168.0901437	-0.04	0.07	9.8132
	246.7187	237.0966707	0.03	**-0.11**	**9.1086**
	275.2823	264.5462903	0.01	**0.18**	**10.4036**
	311.5561	299.4054121	0.01	0.05	16.4988
	503.379	483.747219	-0.01	-0.05	24.9142

	622.0997	597.8378117	0	-0.06	1.5323
	695.7904	668.6545744	0	-0.22	3.4698
	53.0525	50.9834525	0.1	**-0.17**	**13.316**
Pb ← As	162.183	155.857863	0.03	-0.06	4.2327
	205.0741	197.0762101	-0.01	0.08	7.7673
	244.0821	234.5628981	0.03	**0.1**	**23.7203**
	577.0318	554.5275598	0	**-0.1**	**5.7874**
